# Prostate lineage-specific metabolism governs luminal differentiation and response to antiandrogen treatment

**DOI:** 10.1038/s41556-023-01274-x

**Published:** 2023-12-04

**Authors:** Jenna M. Giafaglione, Preston D. Crowell, Amelie M. L. Delcourt, Takao Hashimoto, Sung Min Ha, Aishwarya Atmakuri, Nicholas M. Nunley, Rachel M. A. Dang, Mao Tian, Johnny A. Diaz, Elisavet Tika, Marie C. Payne, Deborah L. Burkhart, Dapei Li, Nora M. Navone, Eva Corey, Peter S. Nelson, Neil Y. C. Lin, Cedric Blanpain, Leigh Ellis, Paul C. Boutros, Andrew S. Goldstein

**Affiliations:** 1 Molecular Biology Interdepartmental Program, University of California, Los Angeles, Los Angeles, CA USA; 2 Department of Molecular, Cell, and Developmental Biology, University of California, Los Angeles, Los Angeles, CA USA; 3 Department of Integrative Biology and Physiology, University of California, Los Angeles, Los Angeles, CA USA; 4 Department of Urology, David Geffen School of Medicine, University of California, Los Angeles, Los Angeles, CA USA; 5 Jonsson Comprehensive Cancer Center, University of California, Los Angeles, Los Angeles, CA USA; 6 Laboratory of Stem Cells and Cancer, WEL Research Institute, Université Libre de Bruxelles (ULB), Brussels, Belgium; 7 Department of Mechanical & Aerospace Engineering, University of California, Los Angeles, Los Angeles, CA USA; 8 Department of Cancer Biology, Dana-Farber Cancer Institute, Boston, MA USA; 9 Fred Hutchinson Cancer Center, Seattle, WA USA; 10 Department of GU Medical Oncology, MD Anderson Cancer Center, Houston, TX USA; 11 University of Washington, Seattle, WA USA; 12 Department of Bioengineering, University of California, Los Angeles, Los Angeles, CA USA; 13 Institute for Quantitative and Computational Biosciences, University of California, Los Angeles, Los Angeles, CA USA; 14 Department of Medicine, Cedars-Sinai Medical Center, Los Angeles, CA USA; 15 Cedars-Sinai Samuel Oschin Comprehensive Cancer Institute, Los Angeles, CA USA; 16 Department of Biomedical Sciences, Cedars-Sinai Medical Center, Los Angeles, CA USA; 17 Center for Bioinformatics and Functional Genomics, Cedars-Sinai Medical Center, Los Angeles, CA USA; 18 Department of Human Genetics, University of California, Los Angeles, Los Angeles, CA USA; 19 Department of Medical Biophysics, University of Toronto, Toronto, Ontario Canada; 20 Vector Institute, Toronto, Ontario Canada; 21 Institute for Precision Health, University of California, Los Angeles, Los Angeles, CA USA; 22 Eli and Edythe Broad Stem Cell Research Center, University of California, Los Angeles, Los Angeles, CA USA; 23 Molecular Biology Institute, University of California, Los Angeles, Los Angeles, CA USA

**Keywords:** Prostate cancer, Differentiation

## Abstract

Lineage transitions are a central feature of prostate development, tumourigenesis and treatment resistance. While epigenetic changes are well known to drive prostate lineage transitions, it remains unclear how upstream metabolic signalling contributes to the regulation of prostate epithelial identity. To fill this gap, we developed an approach to perform metabolomics on primary prostate epithelial cells. Using this approach, we discovered that the basal and luminal cells of the prostate exhibit distinct metabolomes and nutrient utilization patterns. Furthermore, basal-to-luminal differentiation is accompanied by increased pyruvate oxidation. We establish the mitochondrial pyruvate carrier and subsequent lactate accumulation as regulators of prostate luminal identity. Inhibition of the mitochondrial pyruvate carrier or supplementation with exogenous lactate results in large-scale chromatin remodelling, influencing both lineage-specific transcription factors and response to antiandrogen treatment. These results establish reciprocal regulation of metabolism and prostate epithelial lineage identity.

## Abstract (web summary)

Giafaglione et al. define metabolic regulation of prostate epithelial lineage identity and show that modulation of lactate metabolism alters response to antiandrogen therapy.

## Main

Prostate epithelium contains basal and luminal cells as well as rare neuroendocrine cells^1^. Adult mouse prostate basal and luminal cells are predominantly self-sustained under physiological conditions^2^. Luminal differentiation from basal progenitors occurs during development^3,4^, tissue regeneration^5^, inflammation^6^ and prostate cancer initiation^2,7^. Epigenetic changes facilitate the establishment and maintenance of prostate epithelial identity^8–11^. How upstream signalling contributes to the downstream epigenetic regulation of prostate lineage identity remains poorly understood. Metabolism is a key upstream regulator of the epigenome. Most chromatin-modifying enzymes require intermediates of cellular metabolism as substrates or cofactors^12,13^. While metabolic rewiring can modulate differentiation in a wide variety of tissue systems^14–16^, the interplay between metabolic signalling and lineage identity in the prostate remains to be elucidated.

To fill this gap, we sought to understand prostate epithelial cell-type-specific metabolic features. We developed an approach to perform metabolic profiling and heavy isotope nutrient tracing on primary prostate epithelial cells, finding that basal and luminal cells have distinct metabolic signatures. We demonstrate that basal-to-luminal differentiation is associated with increased pyruvate oxidation. Pharmacological inhibition of mitochondrial pyruvate transport, or genetic deletion of *Mpc1*, antagonizes luminal features. Both lactate supplementation and inhibition of lactate efflux block luminal differentiation, suggesting that intracellular lactate accumulation mediates the effect on lineage identity. Inhibition of the mitochondrial pyruvate carrier (MPC) and supplementation with exogenous lactate reprogramme the chromatin landscape of key lineage-specific transcription factors and modulate response to antiandrogen treatment. Our results indicate that prostate epithelial cells have lineage-rooted metabolic features and that modulation of metabolism can govern prostate lineage transitions through epigenetic mechanisms.

## Results

### Basal and luminal cells have distinct metabolic features

**Fig. 1:**
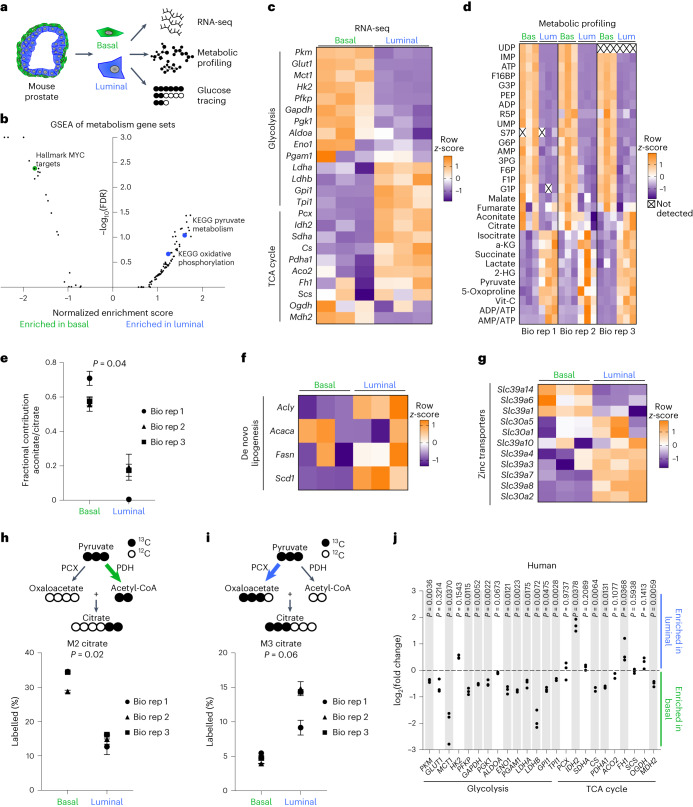
Primary basal and luminal prostate cells have distinct metabolic features. **a**, Schematic of RNA-seq, metabolic profiling and glucose tracing performed on primary basal and luminal cells isolated from mouse prostate. **b**, GSEA of all KEGG, Hallmark and Reactome metabolism gene sets in basal and luminal cells. **c**, Heatmap of glycolytic and TCA cycle enzymes from RNA-seq of three biological replicates of basal and luminal cells. **d**, Heatmap of metabolite abundance in primary basal and luminal mouse prostate cells with three technical replicates for each of the three biological replicates. **e**, Aconitate-to-citrate fractional contribution ratio in primary basal and luminal mouse prostate cells fed [U-^13^C]glucose tracer for 16 h. **f**,**g**, Heatmaps of genes involved in de novo lipogenesis (**f**) and zinc transport (**g**) from RNA-seq of primary basal and luminal mouse prostate cells. **h**,**i**, Percentage M2 citrate (**h**) and percentage M3 citrate (**i**) from [U-^13^C]glucose in basal and luminal cells (*n* = 3 technical replicates for each of the 3 biological replicates). **j**, Fold change in glycolytic and TCA cycle enzymes from RNA-seq of basal and luminal cells from three human prostates. Shaded grey rectangles indicate genes that have statistically significant (*P* < 0.05) differential abundance. For all panels, data are shown as mean ± s.e.m. *P* values were calculated using a paired two-tailed *t*-test. Bas, basal; Bio rep, biological replicate; Lum, luminal. Source data

**Extended Data Fig. 1:**
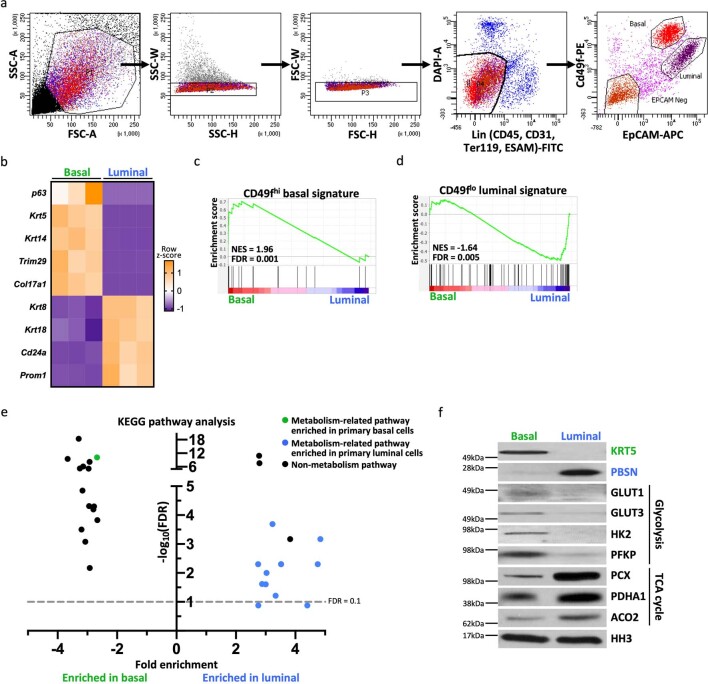
Validation of basal and luminal mouse prostate cell isolation and evaluation of metabolic pathways. (a) Gating scheme for isolating primary basal and luminal cells from mouse prostate. (b) Heatmap of select canonical basal and luminal markers from RNA sequencing of primary basal and luminal mouse prostate cells with three biological replicates. (c) Gene set enrichment analysis (GSEA) showing positive enrichment of CD49f^high^ signature in basal cells relative to luminal cells. (d) GSEA showing positive enrichment of CD49f^low^ signature^18^ in luminal cells relative to basal cells. (e) 30 pathways most enriched in differentially abundant genes (log_2_(fold change) ≥ 1, FDR < 0.2) in basal and luminal cells identified by KEGG pathway analysis. Metabolism-related pathways highlighted in green (basal-enriched) and blue (luminal-enriched). (f) Western blot analysis of select glycolytic and TCA cycle enzymes in basal and luminal cells. Source data

**Extended Data Fig. 2:**
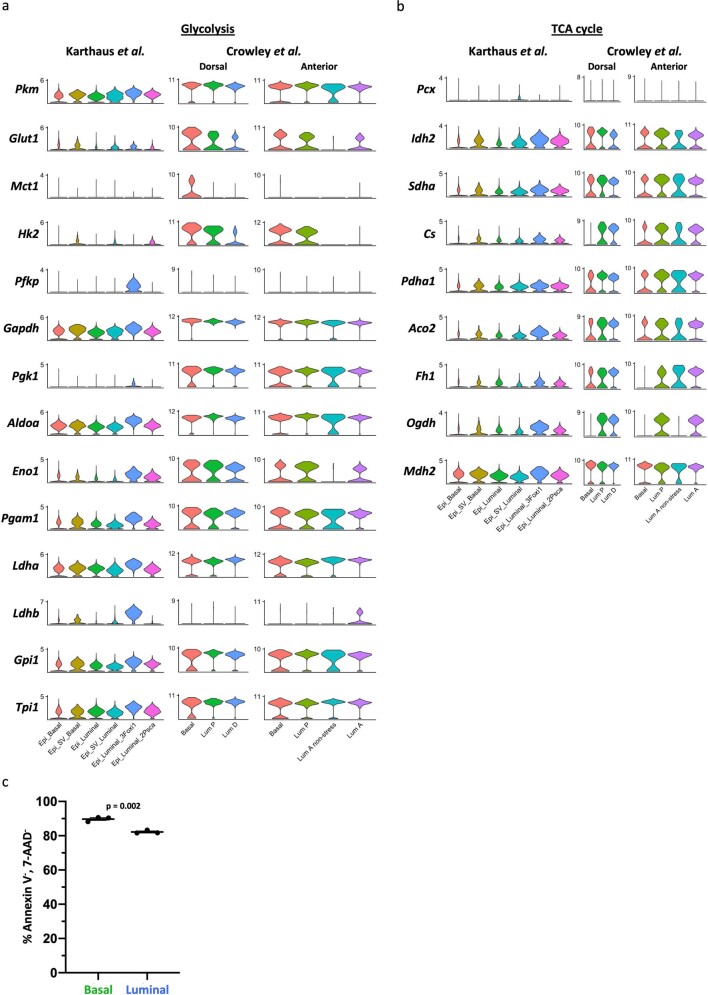
Basal and luminal prostate epithelial cells have distinct metabolic features. (a-b) Analysis of glycolytic (a) and TCA cycle (b) enzymes in Karthaus et al^25^. and Crowley et al^20^. mouse single cell RNA sequencing data. (c) Percentage of Annexin V^−^, 7-AAD^−^ primary basal and luminal cells after overnight culture (n = 3 independent biological replicates). Error bars represent SEM. p-values were calculated using an unpaired two-tailed t-test with Welch’s correction. Source data

We first sought to investigate the relationship between prostate epithelial cell type and metabolic identity. We analysed adult murine prostates using fluorescence-activated cell sorting (FACS) to isolate primary basal (EpCAM^+^ CD49f^high^) and luminal (EpCAM^+^ CD49f^low^) cells (Extended Data Fig. 1a). We first interrogated previous RNA-sequencing (RNA-seq) results^17^ and then performed metabolic profiling and glucose tracing (Fig. 1a). Transcriptional analysis of canonical lineage markers validated isolation of epithelial cell populations (Extended Data Fig. 1b). Gene set enrichment analysis (GSEA) demonstrated appropriate enrichment of the Smith et al.^18^ basal and luminal signatures (Extended Data Fig. 1c,d and Supplementary Table 1). Of the 30 pathways most statistically enriched in differentially abundant genes, 12 were metabolism-related (Extended Data Fig. 1e and Supplementary Table 2). We performed GSEA on all Hallmark, Reactome and Kyoto Encyclopedia of Genes and Genomes (KEGG) metabolism gene sets and identified enrichment of MYC targets in basal cells and enrichment of pyruvate metabolism and oxidative phosphorylation in luminal cells (Fig. 1b and Supplementary Table 3). We also found that basal cells exhibit elevated RNA and protein abundance of several glycolytic enzymes and transporters, while luminal cells exhibit elevated levels of many key tricarboxylic acid (TCA) cycle enzymes (Fig. 1c and Extended Data Fig. 1f). Analysis of mouse prostate single-cell RNA-sequencing (scRNA-seq) data^19,20^ corroborated differential expression of metabolic enzymes in distinct epithelial subsets (Extended Data Fig. 2a,b).

After identifying candidate cell-type-specific metabolic features, we established an approach that enabled us to perform metabolic characterization of distinct prostate epithelial cell types using metabolic profiling and nutrient tracing. Primary cells isolated by FACS were cultured overnight to enhance cell attachment and enable equilibration before metabolite extraction. Annexin V and 7-Aminoactinomycin D (7-AAD) analysis illustrated that adherent basal and luminal cells both exhibit greater than 80% viability after overnight culture (Extended Data Fig. 2c), validating that metabolomics was performed on healthy cell populations. Basal cells have elevated levels of key glycolytic metabolites including PEP, 3PG and F6P, while luminal cells have elevated levels of TCA cycle intermediates including isocitrate, aKG and succinate (Fig. 1d and Supplementary Table 4).

[U-^13^C]glucose tracing revealed a reduction in incorporation of glucose-derived carbon from citrate to aconitate specifically in luminal cells, but not in basal cells (Fig. 1e and Supplementary Table 4). This metabolic wiring may enable luminal cells to secrete high levels of citrate found in seminal fluid^21^ or to utilize citrate for lipid synthesis. Consistent with this hypothesis, we observed increased RNA abundance of genes involved in de novo lipogenesis in luminal cells relative to basal cells (Fig. 1f). Previous studies have reported that zinc accumulation in the prostate epithelium inhibits aconitase activity to prevent citrate oxidation and promote citrate secretion^22^. We evaluated expression of zinc transporters and found that several are elevated in luminal cells relative to basal cells (Fig. 1g). [U-^13^C]glucose tracer analysis also illustrated that basal cells preferentially generate M2 citrate through pyruvate dehydrogenase activity (Fig. 1h and Supplementary Table 4), while luminal cells preferentially generate M3 citrate through pyruvate carboxylase activity (Fig. 1i and Supplementary Table 4). These data indicate that basal and luminal cells have distinct metabolite abundance profiles and nutrient utilization patterns.

We next asked whether cell-type-specific metabolic features are conserved across species. We used a dataset of RNA-seq of benign prostatic basal and luminal epithelial populations from three human prostates^23^. All glycolytic enzymes and transporters evaluated, except *HK2*, were enriched in basal cells, while many TCA cycle enzymes were enriched in luminal cells (Fig. 1j). Our data provide the most comprehensive evidence to date that distinct prostate epithelial cell types contain unique metabolic features.

### Increased pyruvate oxidation with luminal differentiation

**Fig. 2:**
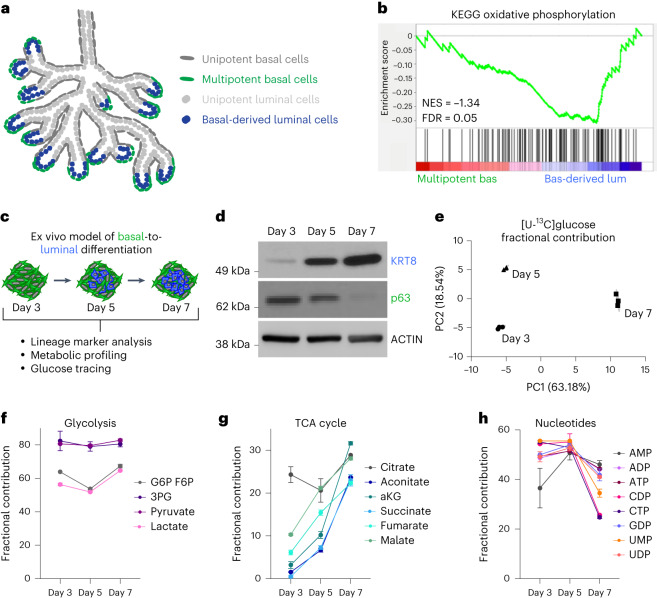
Basal-to-luminal differentiation is accompanied by increased pyruvate oxidation. **a**, Schematic of in vivo model of basal-to-luminal differentiation in P10–P12 murine prostate. **b**, GSEA showing enrichment of KEGG oxidative phosphorylation in basal-derived luminal cells relative to multipotent basal cells in vivo. **c**, Schematic of lineage marker analysis, metabolic profiling and glucose tracing performed on primary basal-derived mouse organoids 3, 5 and 7 d after plating into organoid culture. **d**, Western blot analysis of the luminal marker KRT8 and the basal marker p63 in basal-derived organoids. **e**, PCA of fractional contribution from [U-^13^C]glucose metabolic tracing data of basal-derived organoids with three technical replicates per timepoint. Organoids were cultured with [U-^13^C]glucose 48 h before collecting metabolites at each timepoint. **f**–**h**, Fractional contribution from [U-^13^C]glucose to glycolytic (**f**), TCA cycle (**g**) and nucleotide intermediates (**h**) in basal-derived organoids with three technical replicates per timepoint. For all panels, data are shown as mean ± s.e.m. NES, normalized enrichment score; PC1, principal component 1; PC2, principal component 2. Source data

**Extended Data Fig. 3:**
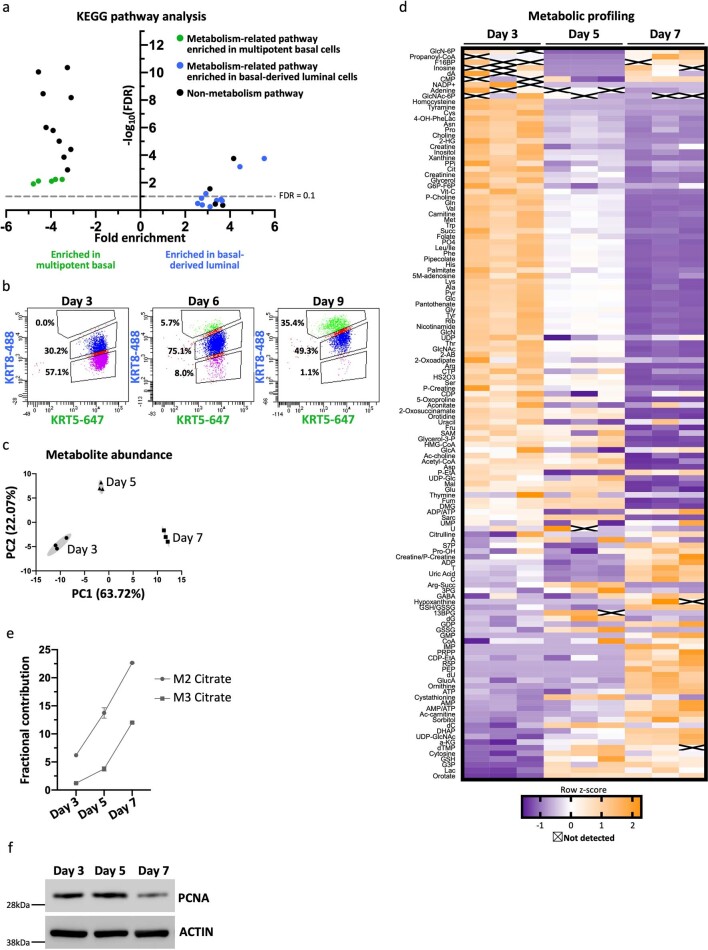
Basal to luminal differentiation is associated with metabolic reprogramming. (a) 30 pathways most enriched in differentially abundant genes (log_2_(fold change) ≥ 1) in multipotent basal cells and basal-derived luminal cells identified by KEGG pathway analysis. Metabolism-related pathways highlighted in green (enriched in multipotent basal-enriched) and blue (enriched in basal-derived luminal-enriched). (b) Intracellular flow cytometry analysis of the basal marker cytokeratin 5 (KRT5) and the luminal marker cytokeratin 8 (KRT8) in primary basal-derived mouse organoids three, six and nine days after plating into organoid culture. (c) Principal component analysis of metabolic profiling data for basal-derived organoids with three technical replicates per timepoint. (d) Heatmap of metabolite abundance in primary basal-derived mouse organoids (n = 3 independent biological replicates per timepoint). (e) Fractional contribution from [U-^13^C]glucose to M2 and M3 citrate in basal-derived organoids (n = 3 independent biological replicates per timepoint). Error bars represent SEM. (f) Western blot analysis of proliferation marker PCNA in basal-derived organoids. Source data

We next sought to investigate whether there is in vivo evidence of metabolic reprogramming during basal-to-luminal differentiation. We took advantage of the spatial restriction of multipotent basal cells at the distal region (tip to 100 μm from the distal tip) of the developing prostate at postnatal day (P)10 (ref. ^24^) (Fig. 2a). Comparing RNA expression in multipotent basal cells and basal-derived luminal cells isolated by FACS, we found 15 of the 30 most enriched pathways identified by KEGG pathway analysis are metabolism-related (Extended Data Fig. 3a and Supplementary Table 5). GSEA revealed negative enrichment of genes in KEGG oxidative phosphorylation in multipotent basal cells relative to basal-derived luminal cells (Fig. 2b).

Features of basal-to-luminal differentiation have been reported in prostate organoid culture; however, the induction kinetics of luminal marker expression were previously poorly defined^25^. Western blot analysis revealed that basal-derived organoids initially express high levels of the basal marker Trp63 (p63) but low levels of the luminal marker cytokeratin 8 (KRT8) (Fig. 2c,d). By day 5 in ex vivo culture, KRT8 is elevated and p63 is reduced (Fig. 2d). Using intracellular flow cytometry, we established that there is gradual upregulation of KRT8 that continues between days 6 and 9 (Extended Data Fig. 3b).

We performed metabolic profiling and [U-^13^C]glucose tracer analysis 3, 5 and 7 d after plating into organoid culture. Principal component analysis (PCA) of both metabolic profiling data and glucose tracer analysis data illustrates that each timepoint clusters independently (Fig. 2e and Extended Data Fig. 3c). Heatmap visualization also demonstrates that primary basal-derived organoids have differences in their metabolite abundance profiles at each timepoint (Extended Data Fig. 3d and Supplementary Table 4). Incorporation of glucose-derived carbon into glycolytic metabolites does not substantially change from day 3 to day 7 (Fig. 2f and Supplementary Table 4). In contrast, fractional contribution to TCA cycle intermediates increases as basal-derived organoids acquire luminal features (Fig. 2g, Extended Data Fig. 3e and Supplementary Table 4). Both fractional contribution to nucleotide intermediates and expression of the proliferation marker PCNA decrease between days 5 and 7 (Fig. 2h, Extended Data Fig. 3f and Supplementary Table 4). These data suggest that increased pyruvate oxidation is unlikely to be driven predominantly by organoid growth, but rather represents a shift in metabolism with luminal differentiation. Collectively, our data indicate basal-to-luminal differentiation is associated with metabolic rewiring, which includes a shift towards increased glucose oxidation.

### The MPC regulates cell fate

**Fig. 3:**
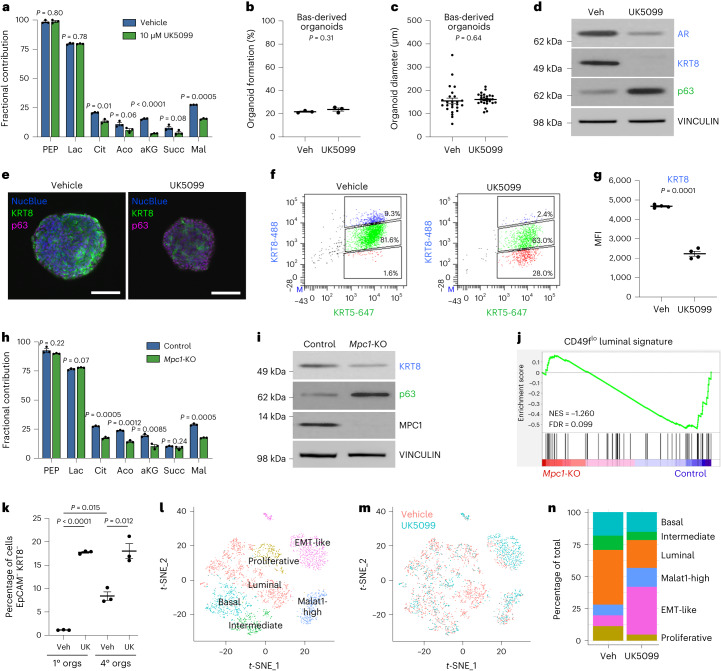
Inhibition or knockout of the MPC prevents basal-to-luminal differentiation. **a**, [U-^13^C]glucose tracer analysis of vehicle- and 10 μM UK5099-treated basal-derived organoids 7 d after plating (*n* = 3 independent biological replicates). Data are shown as mean ± s.e.m. **b**,**c**, Percentage organoid formation (*n* = 3 independent biological replicates) (**b**) and organoid diameter (*n* = 25 independent biological samples) (**c**) of vehicle- and 10 μM UK5099-treated basal-derived organoids 7 d after plating. **d**, Western blot analysis of luminal markers androgen receptor (AR) and KRT8 and basal marker p63 in vehicle- and 10 μM UK5099-treated basal-derived organoids 7 d after plating. **e**, Immunofluorescence of luminal marker KRT8 and basal marker p63 in representative vehicle- and 10 μM UK5099-treated basal-derived organoids 7 d after plating. Scale bars, 100 μm. **f**, Intracellular flow cytometry of KRT8 and basal marker cytokeratin 5 (KRT5) in vehicle- and 10 μM UK5099-treated basal-derived organoids 7 d after plating. **g**, Quantification of mean fluorescence intensity (MFI) of KRT8 from panel **f** (*n* = 4 independent biological replicates). **h**, [U-^13^C]glucose tracer analysis of control and *Mpc1*-KO basal-derived organoids (*n* = 3 independent biological replicates). Data are shown as mean ± s.e.m. **i**, Western blot analysis of basal and luminal markers in control and *Mpc1*-KO basal-derived organoids. **j**, GSEA showing negative enrichment of CD49f^low^ luminal signature^18^ in *Mpc1*-KO relative to control basal-derived organoids. **k**, Flow cytometry quantification of percentage of EpCAM^−^KRT8^−^ cells in vehicle- and 10 μM UK5099-treated primary and quaternary basal-derived organoids (*n* = 3 independent biological replicates). **l**, *t*-Distributed stochastic neighbour embedding (*t*-SNE) plot of scRNA-seq data on quaternary prostate organoids illustrating distinct cell populations. **m**, *t*-SNE plot of vehicle- and 10 μM UK5099-treated cells from scRNA-seq data. **n**, Quantification of percentage of vehicle- and 10 μM UK5099-treated cells in each cluster from scRNA-seq data. For all panels, error bars represent s.e.m. *P* values were calculated using an unpaired two-tailed *t*-test with Welch’s correction. Aco, aconitate; Cit, citrate; EMT, epithelial–mesenchymal transition; Lac, lactate; Mal, malate; orgs, organoids; Succ, succinate; UK, UK5099; Veh, vehicle. Source data

The MPC transports pyruvate from the cytosol to the mitochondria, where it can be oxidized to fuel the TCA cycle^26^. As basal-to-luminal differentiation is associated with increased pyruvate oxidation, we investigated the effects of inhibiting the MPC with the small-molecule MPC inhibitor UK5099. [U-^13^C]glucose tracer analysis confirmed that UK5099 significantly reduces incorporation of glucose-derived carbon into TCA cycle intermediates in mouse basal-derived organoids, consistent with its on-target effect (Fig. 3a and Supplementary Table 4). UK5099 does not significantly influence organoid-formation rate (Fig. 3b) or organoid size (Fig. 3c) of basal-derived organoids.

**Extended Data Fig. 4:**
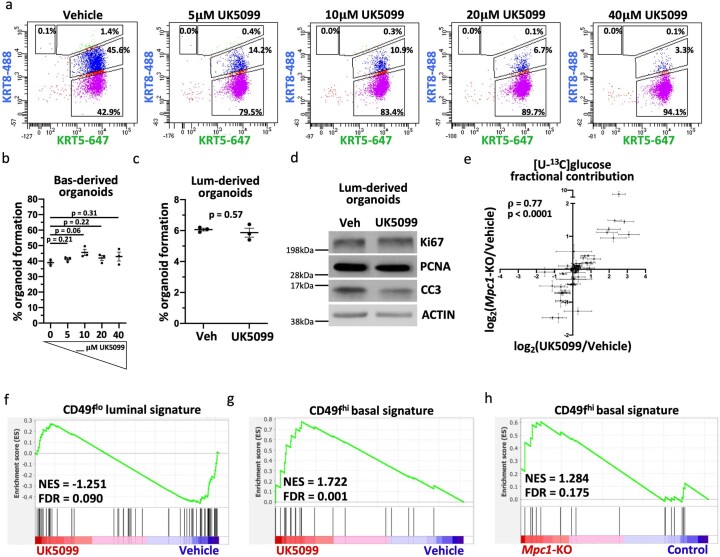
Inhibition of the mitochondrial pyruvate carrier antagonizes luminal lineage identity. (a) Intracellular flow cytometry of KRT8 and KRT5 in basal-derived organoids treated with 0-40 μM UK5099 for seven days. (b) Percent organoid formation of basal-derived organoids treated with 0-40 μM UK5099 (n = 3 independent biological replicates). (c) Percent organoid formation of vehicle- and 10 μM UK5099-treated luminal-derived organoids (n = 3 independent biological replicates). (d) Western blot analysis of proliferation markers (Ki67 and PCNA) and apoptosis marker (CC3, cleaved caspase-3) in vehicle- and 10 μM UK5099-treated luminal-derived organoids seven days after plating. (e) Correlation analysis of [U-^13^C]glucose fractional contribution comparing *Mpc1*-KO and 10 μM UK5099-treated basal-derived organoids (n = 3 independent biological replicates). (f) GSEA showing negative enrichment of CD49f^low^ luminal signature^18^ in 10 μM UK5099-treated relative to vehicle-treated basal-derived organoids. (g) GSEA showing enrichment of CD49f^high^ basal signature^18^ in vehicle-treated relative to 10 μM UK5099-treated basal-derived organoids. (h) GSEA showing enrichment of CD49f^high^ basal signature^18^ in control relative to *Mpc1*-KO basal-derived organoids. For all panels, error bars represent SEM. p-values were calculated using an unpaired two-tailed t-test with Welch’s correction. Correlation analysis was performed using Spearman’s correlation. Source data

Western blot analysis and immunofluorescence illustrate that UK5099 treatment reduces the expression of KRT8 and increases the expression of p63 (Fig. 3d,e). We evaluated KRT8 expression at single-cell resolution using intracellular flow cytometry and found downregulation of KRT8 with UK5099 treatment (Fig. 3f,g). UK5099 treatment reduces KRT8 protein abundance in a dose-dependent manner without altering the rate of organoid formation (Extended Data Fig. 4a,b). UK5099 does not modulate the organoid-forming rate (Extended Data Fig. 4c) or the expression of proliferation and death markers (Extended Data Fig. 4d) in luminal-derived organoids, and thus does not appear to be toxic to them. These data indicate that MPC inhibition is antagonizing luminal differentiation rather than selectively killing cells with a luminal identity.

To complement small-molecule-mediated MPC inhibition, we used a genetics approach to block pyruvate oxidation. [U-^13^C]glucose tracer analysis revealed that *Mpc1* knockout (*Mpc1*-KO) basal-derived organoids have reduced incorporation of glucose-derived carbon into TCA cycle intermediates (Fig. 3h and Supplementary Table 4). We also performed correlation analysis on [U-^13^C]glucose fractional contribution data from 10 μM UK5099-treated and *Mpc1*-KO organoids, which illustrates that MPC inhibition and *Mpc1* knockout have a similar effect on glucose utilization (Extended Data Fig. 4e). Western blot analysis demonstrated that *Mpc1*-KO, as observed with UK5099, reduces luminal lineage markers (Fig. 3i). RNA-seq and GSEA revealed negative enrichment of the luminal signature and positive enrichment of the basal signature in UK5099-treated (Extended Data Fig. 4f,g) and *Mpc1*-KO organoids (Fig. 3j and Extended Data Fig. 4h) relative to control organoids.

**Extended Data Fig. 5:**
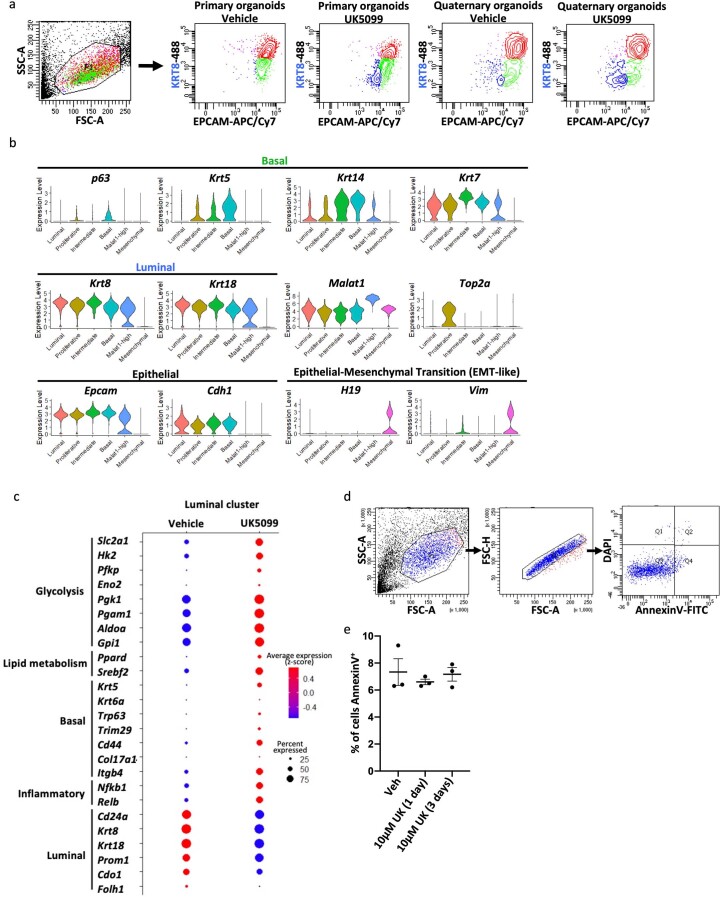
Single cell RNA-sequencing illustrates that inhibition of the mitochondrial pyruvate carrier reduces luminal marker expression and increases expression of basal markers, glycolytic enzymes, and inflammatory signaling genes in the luminal subpopulation in mouse prostate organoids. (a) Representative flow cytometry plots illustrating EPCAM^+^ KRT8^+^ (red), EPCAM^+^ KRT8^−^(green), and EPCAM^−^ KRT8^−^ (blue) populations in primary and quaternary organoids. (b) Expression level (log_2_(read count)) of canonical basal, luminal, epithelial and epithelial-mesenchymal transition (EMT-like) markers in distinct cell populations from scRNA-seq data to validate cluster identification. (c) Dot plot of glycolytic enzymes, lipid metabolism genes, canonical basal markers, inflammatory genes and canonical luminal markers with vehicle or UK5099 treatment within the phenotypic luminal cluster. (d) Flow cytometry gating scheme for apoptosis analysis in panel (e). (e) Quantification of percent of AnnexinV^+^ cells in quaternary organoids treated with vehicle, 10 μM UK5099 for one day, or 10 μM UK5099 for three days (n = 3 independent biological replicates). Error bars in represent SEM. Source data

To further evaluate the role of MPC inhibition in governing prostate lineage identity, we performed scRNA-seq on basal-derived organoids that were passaged weekly for 1 month (quaternary organoids). Only 1% of cells in primary basal-derived organoids are *Epcam*^*−*^*Krt8*^*−*^ (Fig. 3k and Extended Data Fig. 5a). In contrast, quaternary organoids that are maintained in three-dimensional culture for 1 month display features of epithelial–mesenchymal transition, illustrated by an increase in the percentage of *Epcam*^*−*^*Krt8*^*−*^ cells (Fig. 3k and Extended Data Fig. 5a). To understand how MPC inhibition alters lineage identity in a context with greater cellular heterogeneity, we performed scRNA-seq on quaternary organoids treated with vehicle or UK5099 for 3 d. Clustering analysis and annotation of canonical lineage marker expression were used to classify cells into six different cell types (Fig. 3l,m and Extended Data Fig. 5b). The percentage of cells in the phenotypic luminal population (*Krt8*^*+*^ *Krt18*^*+*^ *Krt5*^*−*^ *Trp63*^*−*^) decreases with MPC inhibition, while the percentage of cells in the epithelial–mesenchymal transition-like population (*Epcam*^*−*^ *Cdh1*^*−*^ *Vim*^*+*^) increases with MPC inhibition (Fig. 3n). UK5099 treatment altered gene expression of cells in the luminal population, reducing luminal marker expression while increasing expression of basal markers, glycolytic enzymes and inflammatory signalling genes (Extended Data Fig. 5c). Apoptosis analysis illustrates that UK5099 treatment of quaternary organoids does not increase the percentage of Annexin V^+^ cells (Extended Data Fig. 5d,e), suggesting that MPC inhibition alters lineage identity rather than selects against specific phenotypic populations. Taken together, these data illustrate that modulating metabolism can alter prostate epithelial identity and that the MPC is a key regulator of lineage identity in benign prostate epithelial cells.

### MPC is a regulator of lineage identity in prostate cancer

**Fig. 4:**
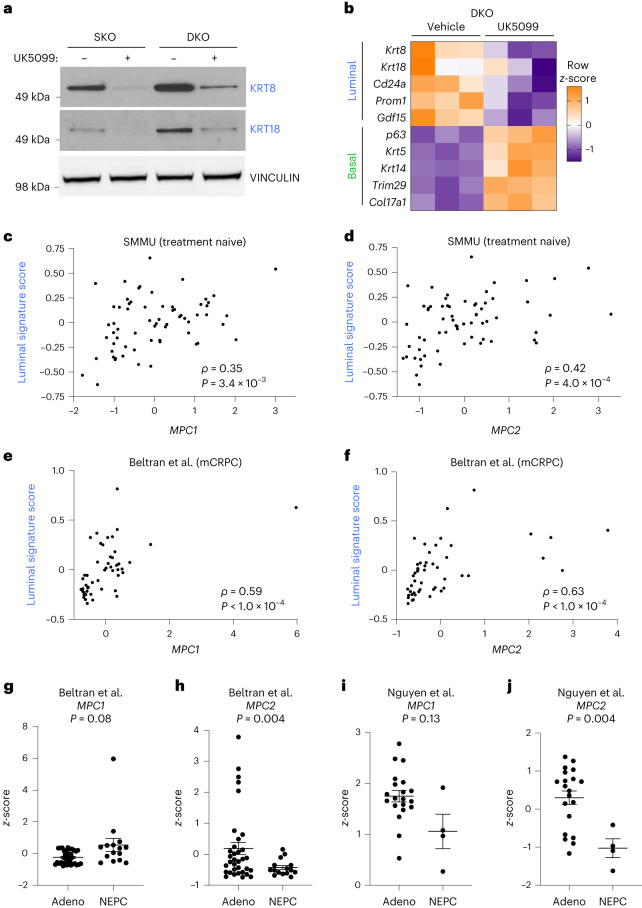
MPC is a regulator of luminal lineage identity in prostate cancer. **a**, Western blot analysis of luminal markers KRT8 and KRT18 in SKO and DKO mouse prostate organoids treated with vehicle or 10 μM UK5099 for 5 d. **b**, Heatmap of canonical basal and luminal markers from RNA-seq of vehicle- and 10 μM UK5099-treated DKO organoids. **c**,**d**, Correlation analysis of luminal signature score and *MPC1* (**c**) or *MPC2* (**d**) *z*-scores in treatment-naive prostate cancer samples from the SMMU dataset^29^. **e**,**f**, Correlation analysis of luminal signature score and *MPC1* (**e**) or *MPC2* (**f**) *z*-scores in metastatic castration-resistant prostate cancer samples from the Beltran et al. dataset^30^. **g**,**h**, RNA abundance of *MPC1* (**g**) or *MPC2* (**h**) in adenocarcinoma (adeno) or neuroendocrine prostate cancer (NEPC) samples from the Beltran et al. dataset^30^. **i**,**j**, RNA abundance of *MPC1* (**i**) or *MPC2* (**j**) in adeno or NEPC samples from the Nguyen et al. PDX dataset^31^. Correlation analysis was performed using Spearman’s correlation with a two-tailed *P* value. For all panels, error bars represent s.e.m. *P* values in **g**–**j** were calculated using an unpaired two-tailed *t*-test with Welch’s correction. mCRPC, metastatic castration-resistant prostate cancer. Source data

**Extended Data Fig. 6:**
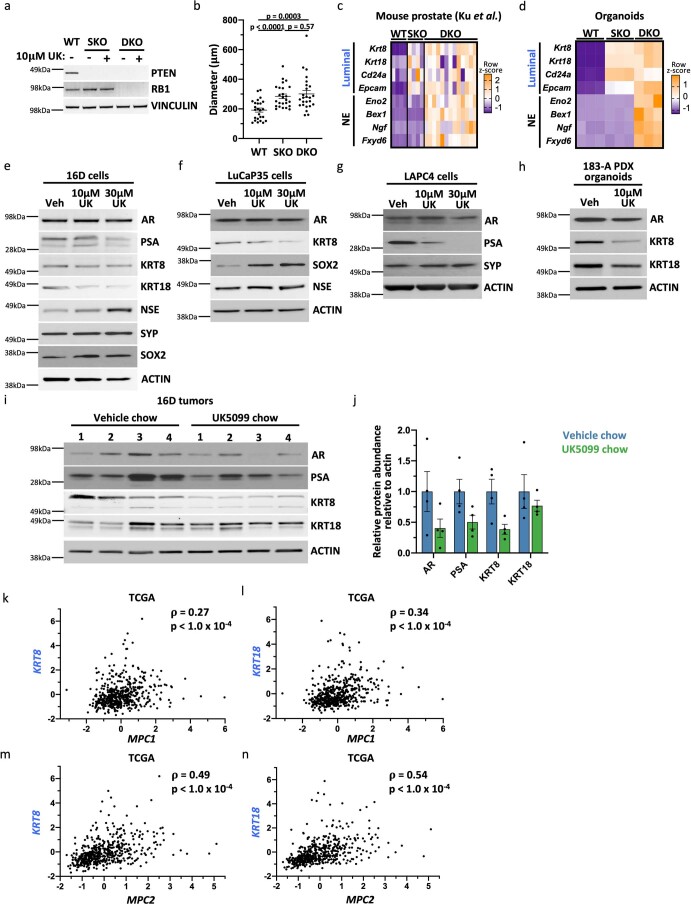
Inhibition of the mitochondrial pyruvate carrier antagonizes luminal lineage identity in models of prostate cancer. (a) Western blot validation of knockout of *Pten* in single knockout (SKO) and double knockout (DKO) mouse prostate organoids and validation of knockout of *Rb1* in DKO organoids. (b) Organoid diameter (micrometers) of wildtype (WT), SKO and DKO organoids (n = 25 independent biological samples). Error bars represent SEM. (c) Heatmap of canonical luminal and neuroendocrine markers in WT, SKO and DKO mouse prostates from RNA sequencing data published in Ku et al^27^. (d) Heatmap of canonical luminal and neuroendocrine markers in WT, SKO and DKO mouse organoids from RNA sequencing data. (e-i) Western blot analysis of lineage markers AR, PSA, KRT8, KRT18, NSE, SYP, and SOX2 in human 16D cell line (e), LuCaP35 cell line (f), LAPC4 cell line (g), 183-A PDX organoids (h), and 16D subcutaneous tumours (i). (j) Quantification of Western blot in panel I (n = 4 independent tumors). Data are shown as mean ± SEM. (k-l) Correlation analysis of z-score expression of the luminal marker *KRT8* (k) or *KRT18* (l) with *MPC1* in 499 primary prostate carcinomas from The Cancer Genome Atlas (TCGA)^28^. (m-n) Correlation analysis of z-score expression of the luminal marker *KRT8* (m) or *KRT18* (n) with *MPC2* in 499 primary prostate carcinomas from TCGA^28^. Correlation analysis was performed using Spearman’s correlation with a two-tailed p-value. p-values in (b) were calculated using an unpaired two-tailed t-test with Welch’s correction. Source data

Loss of tumour suppressor genes *Pten* and *Rb1* is common in prostate cancer^10^, and genetically engineered mouse models of *Pten* loss and combined *Pten*;*Rb1* loss recapitulate key features of prostate adenocarcinoma^27^. Western blot analysis validated tumour suppressor loss in *Pten* single knockout (SKO) and *Pten*;*Rb1* double knockout (DKO) basal-derived organoids (Extended Data Fig. 6a). Both SKO and DKO organoids had significantly larger diameters than benign control organoids (Extended Data Fig. 6b), consistent with a transformed phenotype. RNA-seq data from Ku et al.^27^ illustrate that SKO and DKO mouse prostates have increased expression of canonical luminal markers whereas only DKO prostates have increased expression of neuroendocrine markers relative to wild-type prostates (Extended Data Fig. 6c). We found that both SKO and DKO organoids retain the lineage features of their respective primary tissues (Extended Data Fig. 6d). UK5099 treatment of SKO and DKO organoids reduces the expression of luminal markers KRT8 and KRT18 (Fig. 4a). RNA-seq analysis confirmed reduced expression of canonical luminal markers and increased basal marker expression in UK5099-treated DKO organoids (Fig. 4b). We discovered that UK5099 treatment reduces expression of canonical luminal markers and increases expression of stem-like and neuroendocrine markers in several human prostate cancer models, including 16D cells, LuCaP35 cells, LAPC4 cells and MDA PCa 183-A patient-derived xenograft (PDX) organoids (Extended Data Fig. 6e–h). We also discovered that MPC inhibition antagonizes luminal lineage identity in subcutaneous 16D tumours in vivo (Extended Data Fig. 6i,j). Collectively, these data establish the MPC as a regulator of lineage identity in transformed mouse prostate organoids and human prostate cancer models.

Next, we investigated the relationship between MPC expression and lineage identity in samples from patients with prostate cancer. Correlation analysis of RNA-seq of 499 primary prostate carcinomas from The Cancer Genome Atlas (TCGA)^28^ revealed that abundance levels of *MPC1* and *MPC2* RNA are positively correlated with RNA abundance of the luminal markers *KRT8* and *KRT18* (Extended Data Fig. 6k–n). Furthermore, we calculated luminal signature scores using the Second Military Medical University (SMMU) dataset^29^, which contains RNA-seq of treatment-naive adenocarcinoma prostate tumours, and the Beltran et al. dataset^30^, which contains metastatic castration-resistant adenocarcinoma and neuroendocrine prostate tumours. Abundance levels of *MPC1* and *MPC2* transcripts are positively correlated with luminal signature score in both datasets (Fig. 4c–f). Furthermore, *MPC1* RNA abundance is not significantly different in adenocarcinoma compared with neuroendocrine prostate cancer samples in the Beltran et al. dataset (Fig. 4g). However, *MPC2* RNA abundance is significantly decreased in neuroendocrine tumours compared with adenocarcinoma tumours (Fig. 4h). Similarly, in the Nguyen et al. dataset^31^, *MPC2* is decreased in neuroendocrine compared with adenocarcinoma PDX models (Fig. 4i,j). Importantly, since the MPC complex functions as a heterodimer, loss of MPC2 would yield the complex nonfunctional^32^. These data illustrate that MPC RNA abundance positively correlates with luminal lineage identity across disease states.

### Lactate accumulation results in chromatin remodelling

**Fig. 5:**
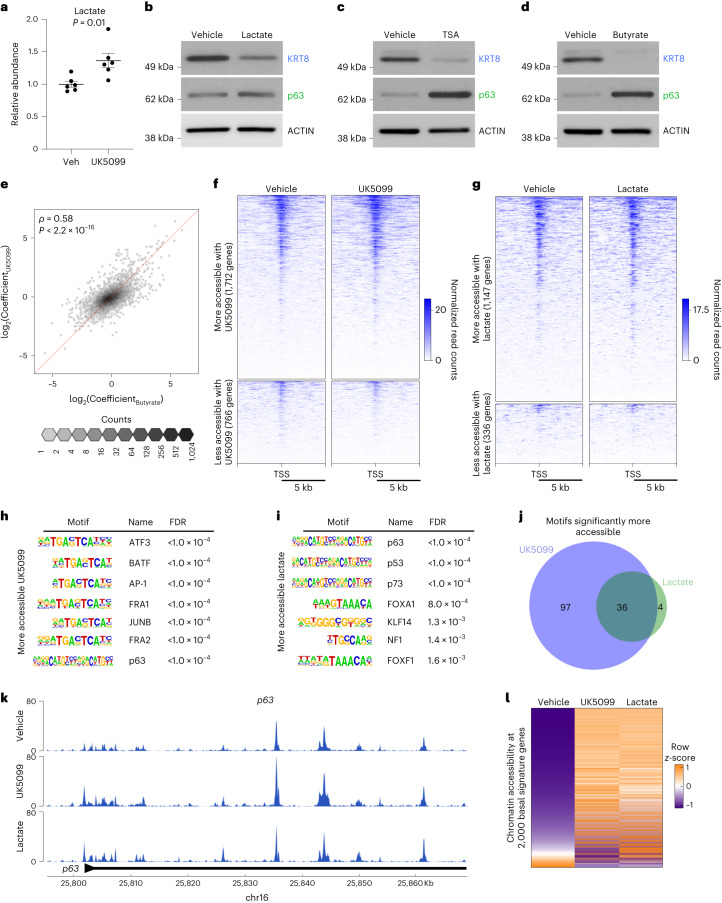
Intracellular lactate accumulation results in large-scale chromatin remodelling of key lineage-specific transcription factors. **a**, Extracellular lactate abundance in primary basal-derived mouse organoids treated with vehicle or 10 μM UK5099 for 7 d (*n* = 6 independent biological replicates). Error bars represent s.e.m. *P* value was calculated using an unpaired two-tailed *t*-test with Welch’s correction. **b**–**d**, Western blot analysis of the luminal marker KRT8 and the basal marker p63 in basal-derived organoids treated with vehicle or 20 mM sodium lactate (**b**), 10 nM Trichostatin A (TSA) (**c**) or 1 mM sodium butyrate (**d**) for 7 d. **e**, Spearman’s correlation between log_2_Coefficients of UK5099 and Butyrate effects for each gene (*r* = 0.58, *P* < 2.2 × 10^−16^). Each hexagonal bin represents a region of the plot with the colour denoting the number of genes that fall within that region. The red dotted line represents *x* = *y*. **f**, Heatmap of 1,712 hyper-accessible genes and 766 hypo-accessible genes (fold change ≥ 1.5 or fold change ≤ 0.5) in basal-derived mouse organoids treated with vehicle or 10 μM UK5099 for 7 d. **g**, Heatmap of 1,147 hyper-accessible genes and 336 hypo-accessible genes (fold change ≥ 1.5 or fold change ≤ 0.5) in basal-derived mouse organoids treated with vehicle or 20 mM sodium lactate for 7 d. **h**,**i**, Seven most significantly enriched transcription factor binding motifs in more accessible regions in organoids treated with 10 μM UK5099 (**h**) or 20 mM sodium lactate (**i**). The FDR was controlled using the Benjamini–Hochberg method. **j**, Venn diagram depicting overlap in significantly enriched transcription factor binding motifs in more accessible regions in UK5099-treated and lactate-supplemented organoids. **k**, Browser track depicting ATAC-seq peaks in *p63* gene in vehicle-treated, UK5099-treated and lactate-supplemented organoids. **l**, Heatmap of chromatin accessibility of 2,000 basal signature genes in vehicle-treated, UK5099-treated and lactate-supplemented organoids. TSS, transcription start site. Source data

**Extended Data Fig. 7:**
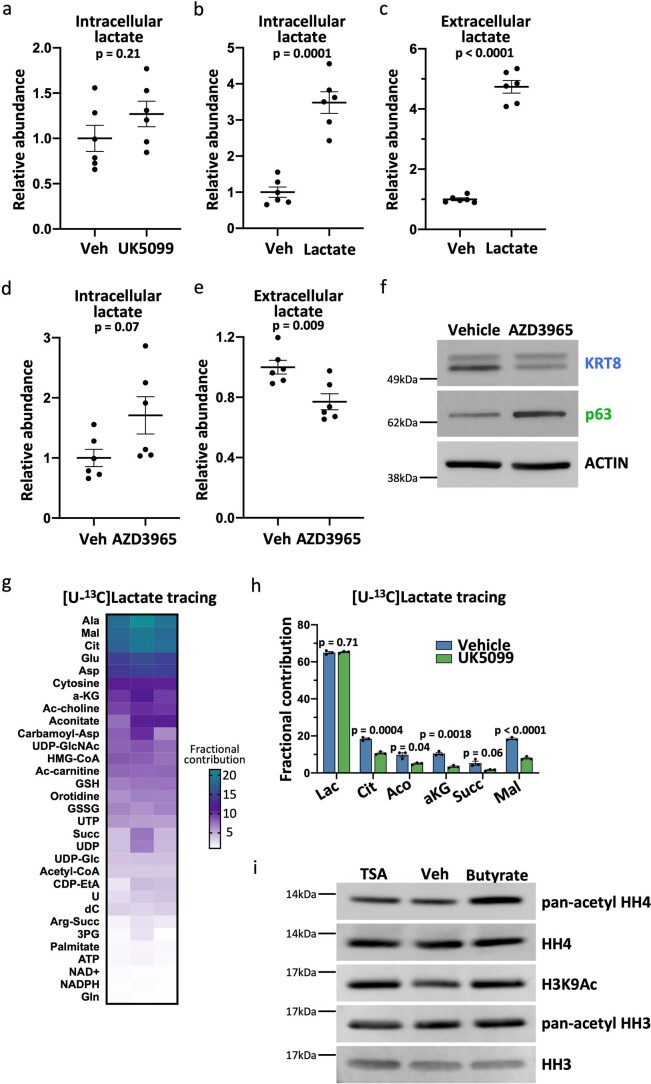
Intracellular lactate accumulation antagonizes luminal lineage identity in mouse prostate organoids. (a-b) Intracellular lactate abundance in primary basal-derived mouse organoids treated with vehicle or 10 μM UK5099 (a) or 20 mM sodium lactate (b) for seven days (n = 6 independent biological replicates). (c) Extracellular lactate abundance in organoids treated with vehicle or 20 mM sodium lactate (n = 6 independent biological replicates). (d-e) Intracellular (d) and extracellular (e) lactate abundance in organoids treated with vehicle or 10 μM AZD3965 (n = 6 independent biological replicates). Error bars in panels (a-e) represent SEM. (f) Western blot analysis of the luminal marker KRT8 and the basal marker p63 in basal-derived organoids treated with vehicle or 10 μM AZD3965 for seven days. (g) Heatmap of fractional contribution of [U-^13^C]lactate tracing data from organoids treated with 20 mM sodium lactate for six days followed by treatment with 20 mM [U-^13^C]lactate for 24 hours. (h) Fractional contribution from [U-^13^C]lactate to TCA cycle intermediates in organoids treated with 20 mM sodium lactate for six days followed by treatment with 20 mM [U-^13^C]lactate and vehicle or 10 μM UK5099 for 24 hours (n = 3 independent biological replicates). Data are shown as mean ± SEM. p-values were calculated using an unpaired two-tailed t-test with Welch’s correction. (i) Western blot analysis of pan-acetyl histone H4 (pan-acetyl HH4), total histone H4 (HH4), H3K9Ac, pan-acetyl histone H3 (pan-acetyl HH3) and total histone H3 (HH3) in histone extracts from basal-derived organoids treated with vehicle, 1 mM sodium butyrate or 10 nM TSA for seven days. Source data

We next sought to elucidate the mechanism by which MPC inhibition antagonizes luminal lineage identity. We hypothesized that MPC inhibition may result in lactate accumulation due to increased availability of pyruvate in the cytosol. Metabolic footprinting and metabolic profiling revealed that both extracellular and intracellular lactate abundance levels are increased with UK5099 treatment (Fig. 5a, Extended Data Fig. 7a and Supplementary Table 4). Therefore, we asked whether lactate supplementation would be sufficient to reduce luminal features in basal-derived prostate organoids. We first validated that 20 mM sodium lactate supplementation increased extracellular and intracellular lactate abundance levels (Extended Data Fig. 7b,c and Supplementary Table 4). Lactate supplementation reduces the protein abundance of KRT8 and increases the protein abundance of p63 (Fig. 5b). To uncouple the effects of extracellular and intracellular lactate accumulation, we used a monocarboxylate transporter 1 (MCT1) inhibitor, AZD3965. As expected, AZD3965 treatment reduces extracellular lactate abundance and results in intracellular lactate accumulation (Extended Data Fig. 7d,e and Supplementary Table 4). Western blot analysis revealed that AZD3965 treatment reduces luminal features (Extended Data Fig. 7f), suggesting that intracellular lactate accumulation drives the effect on lineage identity.

To understand how prostate organoid cells are utilizing supplemented lactate, we performed heavy isotope nutrient tracing on organoids cultured with 20 mM [U-^13^C]lactate. Heavy isotope carbons were detected in metabolites representing various pathways including choline metabolism, pyrimidine synthesis and glutathione metabolism (Extended Data Fig. 7g and Supplementary Table 4). Supplemented lactate fuels the TCA cycle through conversion to pyruvate and entry into the mitochondria (Extended Data Fig. 7h and Supplementary Table 4). Since MPC inhibition and lactate supplementation antagonize luminal identity but have largely opposing effects on metabolism, we hypothesized that the mechanism may be epigenetic in nature. Lactate has been reported to inhibit histone deacetylase (HDAC) activity^33^. We validated the on-target effect of two HDAC inhibitors, Trichostatin A and sodium butyrate (Extended Data Fig. 7i), and found that inhibition of HDAC activity antagonizes luminal identity and enhances basal features in prostate organoids (Fig. 5c,d).

**Extended Data Fig. 8:**
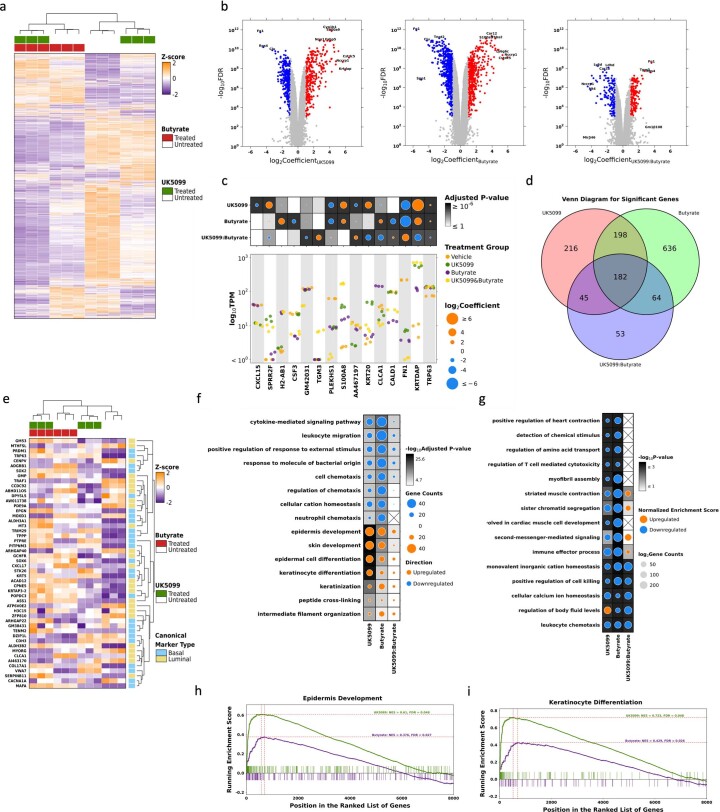
Histone deacetylase inhibition and mitochondrial pyruvate carrier inhibition have similar effects on gene expression in mouse prostate organoids. (a) The distribution of gene abundance (n = 13687) among 12 samples. The primary heatmap color represents the Z-score normalized TPM value of each gene. Missing values were omitted. Hierarchical clustering has been applied to both samples (columns) and genes (rows). The top covariate heatmap indicates the treatment status of each sample. (b) Genes with significant differential RNA abundance were identified based on coefficient and adjusted p-value values (|log_2_(Coefficient)| > 1, adjusted p-value < 0.01). Genes with downregulated RNA abundance are denoted by blue dots, while genes with upregulated RNA abundance are represented by red dots. Grey dots represent genes without a significant difference. Labeled genes have either the least adjusted p-values or the greatest |log_2_(Coefficient)| values. (c) Significant genes (adjusted p-value < 0.01) were selected to show univariate effects under different treatments. The primary strip plot shows raw TPM distribution among selected genes with dot colors indicating treatment types. The dotmap above presents the effect sizes and significance of each gene under different treatments. The dot size represents log_2_Coefficient values, while background color is indicative of the adjusted p-value. (d) The Venn diagram illustrates the overlap between effects, as reflected by the number of significant genes (n = 1,394; |log_2_(Coefficient)| > 1, adjusted p-value < 0.01). The model was adjusted using empirical Bayes moderation for standard error, and the false discovery rate (FDR) was controlled using the Benjamini-Hochberg method. (e) The distribution of top basal and luminal gene abundance (n = 50) among 12 samples. The color in the primary heatmap signifies the Z-score normalized TPM value for each gene. Missing values were omitted. Hierarchical clustering has been applied to both samples (columns) and genes (rows). The top covariate heatmap indicates the treatment status for each sample, while the right covariate heatmap identifies the canonical marker type for each gene. (f) Top 15 enriched gene ontology terms in UK5099, Butyrate, and UK5099:Butyrate effects. The direction of regulation is calculated by the normalized enrichment score and is denoted by different colors: orange indicates upregulation, while blue represents downregulation. The dot size corresponds to the number of genes enriched in each gene set, while the background shading indicates the -log_10_adjusted p-value. (g) Top 15 enriched GSEA gene sets in UK5099, Butyrate, and UK5099:Butyrate effects. The direction of regulation is calculated by the normalized enrichment score (NES) and denoted by different colors: orange (upregulation), and blue (downregulation). The dot size corresponds to the number of genes enriched in each gene set, while the background shading indicates the -log_10_p-value. (h) Epidermis development gene set enrichment results. Genes were ranked from high to low based on log_2_Coefficient of UK5099 or Butyrate effect in the general linear model. (i) Keratinocyte differentiation gene set enrichment results. Genes were ranked from high to low based on log_2_Coefficient of UK5099 or Butyrate effect in the general linear model. Source data

To determine whether HDAC inhibition and MPC inhibition have similar effects on gene expression, we performed RNA-seq on primary mouse prostate organoids treated with vehicle, UK5099, sodium butyrate, or UK5099 and sodium butyrate in combination (Extended Data Fig. 8a). Using two-factor, two-level general linear models, we identified genes influenced by each treatment alone and evaluated potential synergy between them. More genes were influenced by sodium butyrate than UK5099 (1,120 versus 674), and there were few interactions, mostly of small effect size (Extended Data Fig. 8b) and reflecting saturation effects rather than synergy (Extended Data Fig. 8c). We found that 60% of genes affected by UK5099 were also affected by sodium butyrate (Extended Data Fig. 8d) and with very similar effect sizes (Fig. 5e). The 25 genes most associated with basal phenotypes and the 25 most associated with luminal phenotypes strongly distinguished the groups, with basal genes such as *p63* and *Krt5* being upregulated after both treatments (Extended Data Fig. 8e). Genes upregulated by either treatment were preferentially involved in development and differentiation (Extended Data Fig. 8f,h,i) while those downregulated tended be involved in immune pathways (Extended Data Fig. 8g). Taken together, these data suggest that MPC inhibition and the subsequent accumulation of lactate may modulate lineage identity through alterations to histone acetylation. Therefore, we performed the assay for transposase-accessible chromatin using sequencing (ATAC-seq) on organoids treated with UK5099 or lactate to elucidate how these metabolic manipulations alter chromatin accessibility. We identified 1,712 genes with increased accessibility and 766 genes with decreased accessibility in UK5099-treated organoids (Fig. 5f and Supplementary Table 6). Lactate-supplemented organoids contain 1,147 hyper-accessible genes and 336 hypo-accessible genes (Fig. 5g and Supplementary Table 7). The global increase in chromatin accessibility observed with UK5099 treatment and lactate supplementation is consistent with the phenotype being mediated by inhibition of HDAC activity.

**Extended Data Fig. 9:**
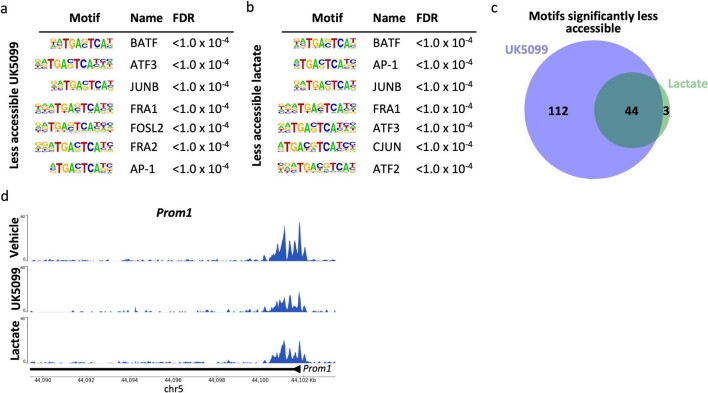
Analysis of less accessible transcription factor binding motifs in mouse prostate organoids treated with UK5099 or lactate supplementation. (a-b) Seven most significantly enriched transcription factor binding motifs in less accessible regions in organoids treated with 10 μM UK5099 (a) or 20 mM sodium lactate (b). The false discovery rate (FDR) was controlled using the Benjamini-Hochberg method. (c) Venn diagram depicting overlap in significantly enriched transcription factor binding motifs in less accessible regions in UK5099-treated and lactate-supplemented organoids. (d) Browser track depicting ATAC-seq peaks in *Prom1* gene in vehicle-treated, UK5099-treated, and lactate-supplemented organoids.

To identify potential regulators of the shift in lineage identity following lactate accumulation, we performed HOMER transcription factor motif analysis on differentially accessible regions in organoids treated with UK5099 or supplemented with lactate. Of the 47 transcription factor motifs significantly less accessible after lactate supplementation, 44 were also significantly less accessible with UK5099 treatment (Extended Data Fig. 9a–c and Supplementary Table 8). HOXB13, a master regulator of prostate luminal identity^34^, is one of the most significantly less accessible binding motifs in both UK5099-treated and lactate-supplemented organoids (Supplementary Table 8). Furthermore, we found that the promoter of the luminal marker *Prom1* is hypo-accessible in both UK5099-treated and lactate-supplemented organoids (Extended Data Fig. 9d). We also identified transcription factor binding motifs in regions that become hyper-accessible after UK5099 treatment and lactate supplementation (Fig. 5h,i). We found that 36 of the 40 transcription factor motifs significantly more accessible with lactate supplementation are also significantly more accessible with UK5099 treatment (Fig. 5j and Supplementary Table 9). One such motif is p63, a master regulator of basal identity^35^. The *p63* promoter itself is also more accessible in organoids treated with UK5099 and exogenous lactate (Fig. 5k). From RNA-seq data, we generated a set of 2,000 genes that are most enriched in primary mouse prostate basal cells relative to luminal cells (Supplementary Table 10). We found that 1,507 of the 2,000 basal-cell-enriched genes are more accessible in both UK5099-treated and lactate-supplemented prostate organoids (Fig. 5l). Collectively, these data suggest that MPC inhibition and lactate supplementation facilitate large-scale chromatin remodelling of key lineage-specific transcription factors and genes.

### Lactate metabolism modulation alters antiandrogen response

**Fig. 6:**
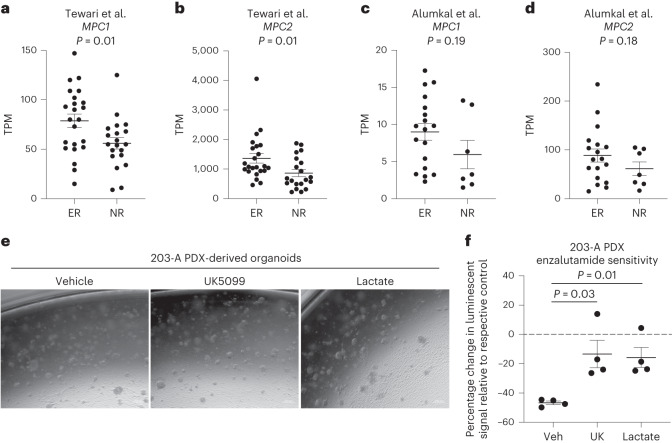
Modulation of lactate metabolism alters antiandrogen response in prostate cancer. **a**,**b**, RNA abundance of *MPC1* (**a**) or *MPC2* (**b**) in nonresponders (NR) or exceptional responders (ER) from the Tewari et al. dataset^36^, which contains RNA-seq of pre-treatment localized prostate cancer biopsies from 43 patients enrolled in neoadjuvant trials of androgen pathway inhibition. **c**,**d**, mRNA abundance of *MPC1* (**c**) or *MPC2* (**d**) in NR or ER from the Alumkal et al. dataset^37^, which contains RNA-seq of metastatic castration-resistant prostate cancer biopsies from 25 patients enrolled in neoadjuvant trials of androgen pathway inhibition. **e**, Representative phase contrast images of MDA PCa 203-A PDX-derived organoids treated with vehicle, 10 μM UK5099 or 20 mM sodium lactate for 7 d. **f**, Percentage change in luminescence signal with 10 μM enzalutamide treatment from CellTiter-Glo assay in castration-resistant MDA PCa 203-A PDX-derived organoids treated with vehicle, 10 μM UK5099 or 20 mM sodium lactate for 7 d before beginning 10 μM enzalutamide treatment (*n* = 4 independent biological replicates). For all panels, error bars represent s.e.m. *P* values were calculated using an unpaired two-tailed *t*-test with Welch’s correction. Source data

**Extended Data Fig. 10:**
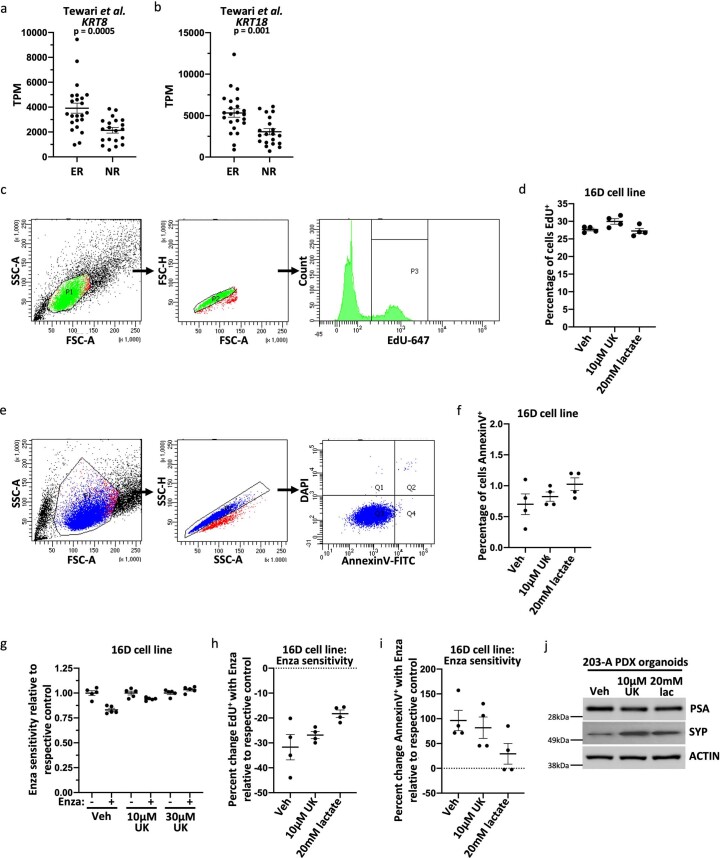
Modulation of lactate metabolism alters antiandrogen response and lineage identity in human models of prostate cancer. (a-b) RNA abundance of KRT8 (a) or KRT18 (b) in non-responders (NR) or exceptional responders (ER) from the Tewari et al. dataset^36^, which contains RNA sequencing of pre-treatment localized prostate cancer biopsies from 43 patients enrolled in neoadjuvant trials of androgen pathway inhibition. p-values were calculated using an unpaired two-tailed t-test with Welch’s correction. (c) Flow cytometry gating scheme for data in panels (d,h). (d) Percentage of cells EdU+ in 16D cells treated with vehicle, 10 μM UK5099, or 20 mM lactate for seven days (n = 4 independent biological replicates). (e) Flow cytometry gating scheme for data in panels (f,i). (f) Percentage of cells AnnexinV+ in 16D cells treated with vehicle, 10 μM UK5099, or 20 mM lactate for seven days (n = 4 independent biological replicates). (g) Luminescence signal relative to respective control from CellTiter-Glo assay in 16D cell line treated with vehicle, 10 μM UK5099, or 30 μM UK5099 for seven days before beginning 48 hours of 10 μM Enzalutamide treatment (n = 5 independent biological replicates). (h-i) Percent change EdU+ (h) and AnnexinV+ (i) with 10 μM Enzalutamide treatment relative to respective control in 16D cell line (n = 4 independent biological replicates). (j) Western blot analysis of PSA and Synaptophysin (SYP) in 203-A PDX-derived organoids. For all panels, error bars represent SEM. Source data

Given that plasticity from a luminal lineage to a cell state with stem-like, basal and/or neuroendocrine features is associated with resistance to androgen pathway inhibitors^27^, we evaluated whether MPC expression is also associated with response to therapy. We used the Tewari et al. dataset^36^, which contains RNA-seq of pre-treatment localized prostate cancer biopsies from 43 patients enrolled in neoadjuvant trials of androgen pathway inhibition, and the Alumkal et al. dataset^37^, which contains RNA-seq of metastatic castration-resistant prostate cancer biopsies from 25 patients enrolled in a clinical trial of androgen pathway inhibition. Exceptional responders to therapy exhibit increased RNA abundance of *KRT8* and *KRT18* relative to nonresponders to therapy (Extended Data Fig. 10a,b), consistent with exceptional responders to therapy having tumours with luminal features. Exceptional responders exhibit increased RNA abundance of both *MPC1* and *MPC2* relative to nonresponders (Fig. 6a–d). Taken together, these data illustrate that high MPC RNA abundance is positively correlated with increased luminal features and better response to androgen pathway inhibition.

Since lactate accumulation antagonizes luminal lineage identity, we hypothesized that metabolic manipulations that increase lactate abundance would lead to increased resistance to the antiandrogen enzalutamide. We first confirmed that MPC inhibition or lactate supplementation does not alter proliferation or apoptosis in the castration-resistant 16D prostate cancer cell line. (Extended Data Fig. 10c–f). We discovered that vehicle-treated cells exhibit sensitivity to enzalutamide, but pre-treatment with UK5099 significantly reduces enzalutamide sensitivity (Extended Data Fig. 10g). Furthermore, the reduction in proliferation and increase in apoptosis induced by enzalutamide is dampened with lactate accumulation (Extended Data Fig. 10h,i). In organoids derived from the castration-resistant MDA PCa 203-A PDX model (Fig. 6e), treatment with UK5099 and treatment with lactate reduce androgen receptor signalling, increase expression of neuroendocrine-like markers (Extended Data Fig. 10j) and reduce enzalutamide sensitivity (Fig. 6f). These data suggest that increasing intracellular lactate abundance can modulate response to antiandrogen treatment.

## Discussion

Recent work has implicated the epigenome in the establishment and maintenance of prostate epithelial cell fate^8–11,27^. Metabolism is a key upstream regulator of the epigenome^13–16^; however, the interplay between metabolic signalling and lineage identity in the prostate was previously poorly understood. Previous studies have demonstrated that modulation of pyruvate and lactate metabolism mediates differentiation phenotypes through a metabolic–epigenetic axis^38,39^. We discovered that both MPC inhibition and exogenous lactate supplementation promote increased chromatin accessibility and global chromatin remodelling of lineage-specific transcription factors in prostate epithelial cells (Fig. 5). We also illustrated that inhibition of HDAC activity antagonizes luminal identity (Fig. 5). Future experiments will be necessary to elucidate which specific epigenetic modifications are responsible for antagonizing luminal differentiation.

At diagnosis, most prostate tumours rely on androgen receptor signalling to promote proliferation^40^. As a result, therapies targeting the androgen receptor signalling axis are initially effective and extend patient survival^41^. The loss of luminal identity is associated with resistance to androgen receptor inhibition^27,42^. We established that MPC inhibition and intracellular lactate accumulation antagonize luminal differentiation (Figs. 3–5). It remains unclear whether metabolic regulation of lineage identity can be exploited to promote the reacquisition of luminal features and restore sensitivity to androgen receptor inhibition in prostate cancer.

Low oxygen, or hypoxia, is a common feature of prostate tumours and is associated with poor outcome^43^. Furthermore, lactate accumulation in prostate tumours increases with Gleason grade^44^. Hypoxia has also been shown to induce prostate cancer plasticity and neuroendocrine differentiation^45^. Under hypoxic conditions, cellular metabolism is rewired towards a glycolytic programme with increased lactate production^46^. Therefore, our work may provide insight into one potential mechanism by which hypoxia and lactate accumulation could promote lineage plasticity.

Distinct cancer types have differing dependencies on the MPC and glycolytic metabolism^47,48^. In prostate cancer, high MPC activity is required for lipogenesis and oxidative phosphorylation, while MPC inactivation suppresses tumour growth^49^. Furthermore, disrupting lactate-dependent lipid rewiring in prostate cancer cells reduces growth and metastasis^50^. Our data suggest MPC inhibition and lactate accumulation may make prostate tumours more resistant to androgen receptor inhibition (Fig. 6). It will be critical to consider how targeting metabolic enzymes and transporters may influence prostate cancer plasticity and response to therapy.

## Methods

All experiments, including animal studies, were conducted in compliance with federal and state government guidelines and followed approved protocols by the Institutional Biosafety Committee and the Institutional Animal Care and Use Committee (IACUC) at the University of California, Los Angeles.

### Animal work

All mice are housed under 12 h:12 h light–dark cycle, with room temperature maintained at 23 °C and relative humidity level of 30–70%. Mouse cages include clean bedding and enrichment materials consistent with IACUC regulations. According to the Animal Research Committee policy on humane treatment and endpoints, mice must be killed if tumours become ulcerated or necrotic, and/or impair normal function. All experiments were terminated before tumours reached this stage.

For experiments described below, animals were housed under the care of the Division of Laboratory Animal Medicine at the University of California, Los Angeles, using protocols approved by the Animal Research Committee (ARC no. 2017-020). Prostates from 3–6-month-old immunocompetent male C57BL/6J mice from Jackson Laboratories were used for primary basal and luminal cell experiments. *Mpc1* floxed male mice were of mixed C57BI/6N and C57BI/6J genetic background^51^. For in vivo UK5099 experiments, 12 million 16D cells were subcutaneously implanted with 100 μl of Matrigel (Corning) into the right flank of NOD-scid-IL2Rg^null^ male mice through a 25-gauge needle under inhalation anaesthesia with 2–3% isoflurane. The mice were fed with either control chow or chow containing 0.08 mg kg^−1^ UK5099 (OpenStandard Diet with 15 kcal% Fat with Blue Dye Irradiated (10–20 kGy), Research Diets) until tumours were formed and collected. PDX MDA PCa 203-A and MDA PCa 183-A tumours were obtained from the MD Anderson Cancer Center^52^. Both 203-A and 183-A PDX models were derived from 58-yr-old males. When these PDX models were originally generated, written, informed consent was obtained from patients before sample acquisition, and all samples were processed according to a protocol approved by the Institutional Review Board of the University of Texas MD Anderson Cancer Center. The studies were conducted in accordance with the Belmont Report and the US Common Rule. Patients were not compensated, and they cannot be identified from data provided in this manuscript. A tumour tissue piece of 50–200 mg was implanted in the right flank of NOD-scid-IL2Rγ^null^ mice subcutaneously through a 5-mm skin incision under inhalation anaesthesia with 2–3% isoflurane. After closing the wound with a surgical clip, 100 ml of Matrigel (Corning 354234) was injected at the implantation site. Carprofen was administered subcutaneously at a dose of 5 mg kg^−1^ after surgery. The surgical clip was removed 1–2 weeks later. When the tumour had grown larger than 500 mm^3^, the mouse was euthanized and the tumour was excised and trimmed and then processed for the experiments, re-implanted or cryopreserved. Cryopreservation of the tissue was done in media with 50% FBS, 40% DMEM and 10% dimethylsulfoxide.

The experiments described below were conducted in compliance with European guidelines regarding animal research and ethical protocols (under protocol numbers 671N and 673N) and approved by the local ethical committee for animal welfare, Comité Ethique du Bien-Être Animal (CEBEA). All animals were housed under standard laboratory conditions in a certified animal facility, receiving food and water ad libitum. Prostates used for isolation of multipotent basal cells and basal-derived luminal cells were collected from CD1 mice purchased from the Jackson Laboratory. The experimental mice used were males of mixed background and at P10–P12 age.

*Pten* floxed and *Pten*;*Rb1* floxed 3-month-old male mice were of mixed C57BL/6:129/Sv:FVB genetic background^27^ and were housed at Harvard Medical School under IACUC-approved protocols.

### Mouse prostate dissociation to single cells

Using a razor blade, individual mouse prostates were mechanically dissociated in dissociation media composed of RPMI 1640 (Gibco) containing 10% FBS (Corning), 1 × penicillin-streptomycin (P/S) (Gibco), 1 mg ml^−1^ collagenase type I (Gibco), 1 mg ml^−1^ dispase (Gibco), 0.1 mg ml^−1^ deoxyribonuclease (Gibco) and 10 μM of the p160ROCK inhibitorY-27632 dihydrochloride (RI) (Tocris Bioscience). When chunks were no longer visible, the samples were incubated at 37 °C on a nutating platform for 1.5 h in 10 ml of dissociation media. After centrifugation at 800*g* for 5 min, the pellet was washed with 1 × PBS. The cell pellet was resuspended in 2.7 ml of 0.05% Trypsin-EDTA (Gibco) and incubated at 37 °C for 5 min. Trypsin was inactivated with 300 ml of dissociation media. Cells were further dissociated by pipetting with a P-1000 pipette and an 18 G syringe. Cells were passed through a 100-μm cell strainer (Corning).

### Staining and sorting cells from mouse prostate for isolation of primary basal and luminal cells

Dissociated cells were stained with directly conjugated primary antibodies rat anti-CD49f-PE (BioLegend 313612, 1:100), rat anti-CD326 (EpCAM)-APC (BioLegend 324207, 1:100), rat anti-CD31-FITC (BioLegend 102405, 1:100), rat anti-CD45-FITC (BioLegend 103108, 1:100), rat anti-Ter119-FITC (BioLegend 116205, 1:100) and rat anti-ESAM-FITC (BioLegend 136205, 1:100) for 20 min on ice. Cells were stained in media containing RPMI 1640 (Gibco), 10% FBS (Corning), 1 × P/S and 10 μM RI. Sorting was performed on a BD FACSAria II (BD Biosciences).

### Bulk RNA-seq

These methods apply to Figs. 1b,c,f,g, 3j and 4b and Extended Data Figs. 1b–e, 4f–h, 6d and 8. RNA was extracted from the cells using the RNeasy Mini Kit (QIAGEN) following the manufacturer’s instructions. Libraries for RNA-seq were prepared with the KAPA Stranded mRNA-Seq Kit (Roche). The workflow consists of messenger RNA enrichment, complementary DNA generation, end repair to generate blunt ends, A-tailing, adaptor ligation and PCR amplification. Different adaptors were used for multiplexing samples in one lane. Sequencing was performed on an Illumina HiSeq 3000 for single-end 1 × 50 runs (Figs. 1b,c, 3j and 4b and Extended Data Figs. 1b–e, 2d,e, 4f–h and 6d) and paired-end 2 × 50 runs (Extended Data Fig. 8).

### Bulk RNA-seq analysis

These methods apply to Figs. 1b,c,f,g, 3j and 4b and Extended Data Figs. 1b–e, 4f–h and 6d. Sequencing quality metrics were generated during sequencing runs using Illumina Sequencing Analysis Viewer. Demultiplexing was performed with Illumina Bcl2fastq (v.2.19.1.403) software. The reads were mapped by STAR 2.7.9a (ref. ^53^) and read counts per gene were quantified using the mouse Ensembl GRCm39.105 GTF file. In Partek Flow v.7.0, read counts were normalized by counts per million (CPM) 1.0 × 10^−4^. All results of differential expression analysis used the statistical analysis tool DESeq2 (v.1.40.2)^54^. KEGG pathway analysis was performed using DAVID Bioinformatics^55,56^. GSEA was performed as described previously using GSEA_4.0.3 software^57,58^.

### Significance testing of RNA-seq of UK5099- and sodium butyrate-treated organoids

These methods apply to Extended Data Fig. 8. To measure the RNA abundance, RNA-seq reads were trimmed using fastp (v.0.20.1)^59^ with default parameters, then mapped to the mouse Ensembl GRCm38-EBI102 using STAR (v.2.7.10a)^53^. STAR alignment was carried out using default settings with an additional argument to include the minimum length of 10 base pairs (bp) for the chimeric junction segment. Aligned reads were quantified using the rsem-calculate-expression program (v.1.3.3)^60^ for transcripts per million (TPM) calculation with default settings. We also assessed read-level quality control metrics using FastQC (v.0.11.8).

To test the combination effect of UK5099 and butyrate, we constructed the following two-factor, two-level linear model:

$$Y = {\alpha }_{0}+{\alpha }_{1}\times \mathrm{UK5099}+{\alpha }_{2}\times \mathrm{Butyrate}+{\alpha }_{3}\times \mathrm{UK5099:Butyrate}$$

Here, *Y* refers to the abundance level of a gene, which is log_2_ transformation of TPM values; ${\alpha }_{0}$ refers to the basal abundance level of that gene; ‘UK5099’ indicates ‘UK5099-dependent, Butyrate-independent’ abundance changes; ‘Butyrate’ indicates ‘Butyrate-dependent, UK5099-independent’ abundance changes; ‘UK5099:Butyrate’ captures ‘UK5099-dependent, Butyrate-dependent’ abundance changes.

We used R package limma (v.3.17)^61^ in R (v.4.2.2) to fit each gene in the RNA-seq to the model. The model was adjusted using empirical Bayes moderation for standard error, and the false discovery rate (FDR) was controlled using the Benjamini–Hochberg method^62^. Genes exhibiting significant changes were identified based on the adjusted *P* < 0.01 and |log_2_(Coefficient)| > 1 threshold. Venn diagrams representing the overall and directional effects were generated using the VennDiagram package in R (v.1.7.3)^63^. The hierarchical clustering heatmap of gene TPM was constructed using R package BoutrosLab.plotting.general (v.7.0.8)^64^.

### Functional enrichment analysis

These methods apply to Extended Data Fig. 8. For genes with differential mRNA abundance calculated based on the coefficient from the general linear model, we ranked the genes according to their log_2_(Coefficient) from high to low. GSEA was then performed using the R package clusterProfiler (v.3.17)^65^. Gene ontology enrichment analysis was conducted for both upregulated genes (log_2_(Coefficient) > 1, −log_10_(FDR) > 1) and downregulated genes (log_2_(Coefficient) < −1, −log_10_(FDR) > 1) using the R package clusterProfiler (v.3.17). The results of both GSEA and gene ontology enrichment analyses were visualized using BoutrosLab.plotting.general (v.7.0.8)^64^.

### scRNA-seq

Basal cells were isolated from *Pten*^fl/fl^;*Rb1*^fl/fl^ mouse prostates and infected with FU-CRW (red fluorescent protein, RFP) lentivirus. Lentiviral spinfections were done by culturing the cells with virus in 200 μl of RPMI 1640 (Gibco) containing 10% FBS (Corning), 1 × (P/S) (Gibco) and 10 μM RI (RPMI, 10% FBS, 1% P/S + RI) plus 8 μg ml^−1^ polybrene for 30 min at 37 °C, then spinning at 300*g* for 90 min. After spinfection, growth factor reduced Matrigel (Corning) was added to the cell suspension at a final concentration of 75% before plating into rings in 24-well plates. Organoids were cultured as previously described^66^ and passaged every 7–10 d. After >4 passages, organoids were treated with vehicle (dimethylsulfoxide) or 10 μM UK5099 for 3 d. Organoids were removed from Matrigel by incubating in Advanced DMEM/F-12 (Gibco) containing 1 mg ml^−1^ dispase (Gibco) and 10 μM RI for 1 h at 37 °C. After centrifugation at 800*g* for 5 min, the pellet was washed with 1 × PBS (Gibco). Organoids were resuspended in 800 μl of 0.05% Trypsin-EDTA (Gibco) and incubated at 37 °C for 5 min. The trypsin was quenched with 200 μl of RPMI, 10% FBS, 1% P/S + RI and organoids were pipetted up and down ten times to dissociate to single cells and passed through a 100-μm cell strainer (Corning). Samples were counted using a Countess II Automated Cell Counter (Thermo Fisher Scientific) and haemocytometer for cell concentration and viability using Trypan Blue stain 0.4% (Invitrogen). Cells were loaded to form gel beads in emulsion (GEMs) and barcode individual cells. GEMs were treated according to the manufacturer’s instructions. Single-cell gene expression libraries were created using Chromium Next GEM Single Cell 3′ (v.3.1 Chemistry) (10x Genomics), Chromium Next GEM Chip G Single Cell Kit (10x Genomics) and Single Index Kit T Set A (10x Genomics) according to the manufacturer’s instructions. Paired-end sequencing was done using an Illumina Novaseq 6000 at a sequencing depth of 492,915,641 and 555,876,242 read pairs for vehicle and UK5099 samples, respectively, with read length of 151 for both read 1 and read 2, and with an 8 bp index read for multiplexing. Basecalling was done using Illumina Casava (v.1.7) software. CellRanger (v.5.1) count was used to create an RNA abundance matrix with --expect-cells=1000 and Mus musculus (mm10) from the Ensembl database as a reference genome^67^. RNA abundance matrices from vehicle- and UK5099-treated samples were loaded into the Seurat (v.3.2.2) R package^68^. DoubletDcon (v.1.1.2) was used to remove potential doublets^69^. Additionally, cells were filtered based on the number of genes (≥250), unique molecular identifiers (≥500) and percentage of mitochondrial genes (<20%). After quality control, log_2_ normalization was performed within each sample using *NormalizeData* function with default parameters. The top 2,000 variable genes were selected using *FindVariableFeatures*. The two samples were integrated together with *FindIntegrationAnchors* and *IntegrateData* functions which incorporate canonical correlation analysis to align cells with similar transcriptomic patterns across samples. PCA was performed after the integration. The top 20 principal components were used to construct the *k*-nearest neighbour graph, followed by Louvain algorithm to cluster cells based on similar gene expression patterns. Cell clusters were visualized using *t*-distributed stochastic neighbour embedding. After, markers for each cluster were determined using FindAllMarkers with average log_2_ fold change > 0.25 and minimum percentage difference > 0.25. Cell types were determined by comparing canonical markers with cluster-specific markers. After cell type identification, cell type proportions were calculated with the number of cells in each cell type divided by the total number of cells in each sample. To see the effect of UK5099 in the luminal cluster, *DotPlot* in Seurat was used to visualize the expression of luminal markers, basal markers, glycolytic enzymes, lipid metabolism genes and inflammatory signalling genes.

### Cell lysis and western blot

Primary basal and luminal cells were sorted and immediately lysed in RIPA buffer (50 mM Tris-HCl pH 8.0, 150 mM NaCl, 1% NP-40, 0.5% sodium deoxycholate, 0.1% SDS, Fisher Scientific) containing a cOmplete protease inhibitor cocktail tablet (Roche) and Halt Phosphatase Inhibitor (Fisher Scientific). Organoids were removed from Matrigel by incubating in Advanced DMEM/F-12 (Gibco) containing 1 mg ml^−1^ dispase (Gibco) and 10 μM RI for 1 h at 37 °C. After centrifugation at 800*g* for 5 min, the pellet was washed with 1 × PBS and immediately lysed in RIPA buffer containing a cOmplete protease inhibitor cocktail tablet and Halt Phosphatase Inhibitor. For tumour lysis, tumour tissue was added to a bead tube (Fisher, 15-340-153) containing 1 ml of RIPA buffer containing a cOmplete protease inhibitor cocktail tablet and Halt Phosphatase Inhibitor on ice. Samples were homogenized for 1 min at maximum speed twice on a bead homogenizer (Fisher). Bead tubes were spun at 17,000*g* at 4 °C for 10 min. The supernatant was transferred to an Eppendorf tube and spun at 17,000*g* at 4 °C for 10 min. Each sample was sonicated for 40 s at 20 kHz with a sonic dismembrator (Fisher Scientific) to improve membranous and nuclear protein yield. Samples were run on NuPAGE 4–12% Bis-Tris Gel (Invitrogen) and transferred onto PVDF membranes (Millipore Sigma). Total protein was visualized using SYPRO RUBY protein blot stain (Fisher Scientific) and membranes were blocked in PBS + 0.1% Tween-20 (Fisher Scientific) + 5% milk (Fisher Scientific). Proteins were probed with primary antibodies followed by chromophore-conjugated anti-mouse (Invitrogen A21235, 1:1,000) or anti-rabbit secondary antibodies (Invitrogen A21244, 1:1,000) or HRP-conjugated anti-mouse (Thermo 31430, 1:10,000) or anti-rabbit secondary antibodies (Thermo 31463, 1:10,000) and detected by florescence or HRP chemiluminescence, respectively. Primary antibodies used were anti-Cytokeratin 5 (Biolegend 905504, 1:3,000), anti-Probasin (Santa Cruz sc-393830, 1:1,000), anti-Glut1 (Abcam ab115730, 1:10,000), anti-Glut3 (Abcam ab191071, 1:1,000), anti-Hexokinase 2 (Cell Signaling 28675, 1:1,000), anti-Phosphofructokinase (Abcam ab204131, 1:5000), anti-Pyruvate carboxylase (Abcam ab128952, 1:1,000), anti-Pyruvate dehydrogenase E1 component subunit alpha (Proteintech 18068-1-AP, 1:1,000), anti-Aconitase 2 (Abcam ab110321, 1:1,000), anti-Histone H3 (Cell Signaling 9717S, 1:1,000), anti-Cytokeratin 8 (Biolegend 904804, 1:1,000), anti-p63 (Biolegend 619002, 1:1,000), anti-beta Actin (Fisher MA1-140, 1:15,000), anti-Proliferating cell nuclear antigen (Fisher 13-3900, 1:1,000), anti-Androgen receptor (Abcam ab133273, 1:1,000), anti-Mitochondrial pyruvate carrier 1 (Cell Signaling 14462, 1:1,000), anti-Ki-67 (Abcam ab15580, 1:1,000), anti-cleaved caspase-3 (Cell Signaling 9661L, 1:500), anti-Cytokeratin 18 (Fisher MA5-12104, 1:100), anti-Vinculin (Abcam Ab129002, 1:1,000), anti-Phosphatase and tensin homologue (Cell Signaling 9559, 1:1,000), anti-Retinoblastoma protein 1 (Abcam ab181616, 1:1,000), anti-Acetyl-histone H3 (Lys9) (Cell Signaling 9649, 1:1,000), anti-Pan-acetyl histone H3 (Active Motif 61637, 1:1,000), anti-Histone H4 (Abcam ab10158, 1:1,000), anti-Pan-acetyl histone H4 (Abcam ab177790, 1:1,000), anti-prostate-specific antigen (Cell Signaling 5877, 1:1,000), anti-neuron-specific enolase (Proteintech 66150-1-Ig, 1:3,000), anti-synaptophysin (Cell Signaling 5461, 1:1,000) and anti-Sox2 (Cell Signaling 14962, 1:1,000).

### Apoptosis assay

Cell culture media and wash media were collected and pooled with quenched trypsin-containing media containing cells and apoptosis analysis was performed using an apoptosis detection kit (BioLegend, 640922) according to manufacturer instructions. Flow cytometry was performed to quantify the percentage of annexin V^−^, 7-AAD^−^ cells.

### Primary cell metabolic profiling and nutrient tracing

Twelve-well plates were coated with a 1/80 dilution of growth factor reduced Matrigel (Corning) in RPMI 1640 (Gibco) to enhance cell attachment. The 1/80 Matrigel coating was aspirated before primary basal and luminal cells were seeded at a density of 200,000 cells per well and 140,000 cells per well, respectively. Cells were cultured overnight in mouse organoid media^66^ containing [U-^13^C]glucose (Cambridge Isotope Laboratories). Before metabolite extraction, tracer-containing medium was aspirated and cells were washed with cold 150 mM ammonium acetate pH 7.3. Metabolite extractions were performed by adding 500 μl of cold 80% methanol to each well and removing cells using a cell scraper. The cell suspension was transferred to an Eppendorf tube and 10 μl of 1 mM norvaline (Sigma) was added as an internal standard. Each sample was vortexed for 30 s and centrifuged at 17,000*g* for 5 min at 1 °C. Then, 420 μl of the supernatant was transferred to an ABC vial (Fisher Scientific) and evaporated using an EZ-2Elite evaporator (Genevac). Samples were stored at −80 °C before analysis.

The liquid chromatography separation using an Ion Chromatography System (ICS) 5000 (Thermo Scientific) was performed on a Dionex IonPac AS11-HC-4μm anion exchange column. The gradient was 5–95 mM KOH over 13 min, followed by 5 min at 95 mM, before re-equilibration to 5 mM. Other liquid chromatography parameters: flow rate 350 µl min^−1^, column temperature 35 °C, injection volume 5 μl. The Q Exactive mass spectrometer (Thermo Scientific) was operated in negative ion mode for detection of metabolites using a resolution of 70,000 at *m*/*z* 200 and a scan range of 70–900 *m*/*z*. Data were extracted using Tracefinder 3.1 (Thermo Scientific). Metabolites were identified based on accurate mass (±5 ppm) and previously established retention times of pure standards.

Normalization was performed by resuspending the cell pellet in 300 μl of lysis solution (0.1 M NaCl, 20 mM Tris-HCl, 0.1% SDS, 5 mM EDTA in distilled water). Samples were syringed with a 25 G needle to reduce viscosity and 50 μl of each sample was transferred to a 96-well black-wall, clear-bottom tissue culture plate (Corning). We added 50 μl of lysis solution to one well for a blank reading. Then, 100 μl of 5 μg ml^−1^ Hoechst 33342 (Invitrogen) in distilled water was added to each well and 96-well plates were incubated for 30 min in the dark at 37 °C before measurement of DNA-based florescence using a Tecan Infinite M1000 plate reader with 355-nm excitation and 465-nm emission. The blank reading was subtracted from each absorbance value to calculate relative cell amount.

### In vivo basal-to-luminal differentiation RNA-seq

#### Cell preparation from postnatal prostates

Prostate tissue of mice at P10–P12 was microdissected under a stereoscope to separate the different lobes. The ventral lobe was used to further separate (by cutting) the tips from the main ducts. The ventral lobes of 20 mice at P10–P12 were used. Tissues were collected in 24-well plates and chopped. Minced tissues were digested in 5 mg ml^−1^ Collagenase Type I (Sigma-Aldrich, diluted in HBSS) for 2 h at 37 °C under agitation. Physical dissociation using a P-1000 pipette was performed every 20 min throughout the enzymatic digestion. Collagenase activity was blocked by adding EDTA (5 mM) for 2 min, followed by 0.25% Trypsin-EDTA for 5 min. Cells were rinsed in HBSS supplemented with 10% FBS and the cell suspensions were filtered through 70-μm cell strainers (BD Bioscience), followed by two successive washes in HBSS supplemented with 2% FBS.

#### Cell labelling, flow cytometry and sorting from postnatal prostates

Samples were incubated in 200 μl of PBS supplemented with 2% FBS with fluorochrome-conjugated antibodies for 30 min on ice protected from light, with shaking every 10 min. Antibodies were washed with 2% FBS/PBS and cells were resuspended in 2.5 mg ml^−1^ DAPI (Invitrogen, D1306) before analysis. The following antibodies were used: PE-conjugated anti-CD45 (rat, clone 30-F11, dilution 1:100, BD Biosciences Cat. no. 553081), PE-conjugated anti-CD31 (rat, clone MEC 13.3, dilution 1:100, BD Biosciences Cat. no. 553373), PE-conjugated anti-CD140a (rat, clone APA5, dilution 1:100, BD Biosciences Cat. no. 624049), APC-conjugated anti-CD49f (rat, clone GoH3, dilution 1:100, eBioscience Cat. no. 17-0495), APC-Cy7-conjugated anti-EpCAM (rat, clone G8.8, dilution 1:100, BioLegend Cat. no. 118218). Living cells were selected by forward and side scatter, doublets discriminating and DAPI dye exclusion. CD45^+^, CD31^+^ and CD140a^+^ cells were excluded (Lin^+^) before analysis of the epithelial basal (EpCam^high^/CD49f^high^) and luminal (EpCam^high^/CD49f^low^) cells. FACS and analysis were performed using FACSAria and LSRFortessa, using FACSDiva software (BD Bioscience). Sorted cells were collected in lysis buffer for RNA extraction (RLT buffer, QIAGEN). The following samples were collected in replicates from P10–P12 prostates: 1,702 basal cells and 1,626 luminal cells from the tips.

#### RNA extraction and RNA-seq

These methods apply to Fig. 2b and Extended Data Fig. 3a. RNA extraction from FACS isolated cells was performed using RNeasy micro kit (QIAGEN) according to the manufacturer’s recommendations. Before sequencing, the quality of RNA was evaluated by Bioanalyzer 2100 (Agilent). Indexed cDNA libraries were obtained using the Ovation Solo RNA-seq Systems (NuGen) following the manufacturer’s recommendations. The multiplexed libraries (11 pM/18 pM) were loaded on flow cells and sequences were produced using a NovaSeq 6000 S2 Reagent Kit (200 cycles from Novaseq 6000 System, Illumina) on a NovaSeq 6000 System (Illumina). Reads were mapped against the mouse reference genome (Grcm38/mm10) using STAR software to generate read alignments for each sample. Annotations for Mus_musculus.GRCm38.87.gtf were obtained from ftp.Ensembl.org. After assembling transcripts, gene level counts were obtained using HTseq and normalized to 20 million aligned reads. Genes with individual sample expression levels lower than 10 and replicate average abundance levels lower than 20 were filtered out. The fold changes of average gene abundance for the replicates were used to calculate the level of differential gene abundance between different cell populations. Genes with a fold change greater than or equal to 2 were considered upregulated and those with a fold change lower than or equal to 0.5 were considered downregulated.

### Organoid culture of primary basal- and luminal-derived mouse organoids

Basal cells were plated at a density of 1,000 cells per well and luminal cells were plated at a density of 20,000 cells per well. Growth factor reduced Matrigel (Corning) was added to the cell suspension at a final concentration of 75% before plating into rings in 24-well plates.

### Viral vectors

A Cre recombinase cassette was inserted into a red fluorescent protein-expressing FU-CRW lentivirus vector^70^ by restriction digestion and ligation at the EcoRI site to make FU-Cre-CRW. Insertion of the Cre cassette was confirmed by DNA sequencing. Concentrated viral preps of FU-Cre-CRW and FU-CRW were made by the University of California, Los Angeles Vector Core facility and the Cre recombinase activity was validated by infecting HEK 293T cells transduced with a Cre-reporter plasmid (Addgene no. 62732)^71^.

### Generation of *Mpc1*-KO, *Pten* SKO and *Pten*;*Rb1* DKO organoids

Basal cells were isolated from *Mpc1*^fl/fl^, *Pten*^fl/fl^ and *Pten*^fl/fl^;*Rb1*^fl/fl^ mouse prostates and infected with RFP (FU-CRW) or Cre-RFP (FU-Cre-CRW) lentivirus. Lentiviral spinfections were done by culturing the cells with virus in 200 μl of RPMI 1640 (Gibco) containing 10% FBS (Corning), 1 × P/S and 10 μM RI (RPMI, 10% FBS, 1% P/S + RI) plus 8 μg ml^−1^ polybrene for 30 min at 37 °C then spinning at 300*g* for 90 min. After spinfection, growth factor reduced Matrigel (Corning) was added to the cell suspension at a final concentration of 75% before plating into rings in 24-well plates. After 1 week of culture, organoids were dissociated to single cells. Organoids were removed from Matrigel by incubating in Advanced DMEM/F-12 (Gibco) containing 1 mg ml^−1^ dispase (Gibco) and 10 μM RI for 1 h at 37 °C. After centrifugation at 800*g* for 5 min, the pellet was washed with 1 × PBS. Organoids were resuspended in 800 μl of 0.05% Trypsin-EDTA (Gibco) and incubated at 37 °C for 5 min. The trypsin was quenched with 200 μl of RPMI, 10% FBS, 1% P/S + RI and organoids were pipetted up and down ten times to dissociate to single cells and passed through a 100-μm cell strainer (Corning). After centrifugation at 800*g* for 5 min, the pellet was washed with 1 × PBS and resuspended in RPMI, 10% FBS, 1% P/S + RI. RFP-positive cells were isolated by FACS. All prostate organoids were cultured based on established protocols^66,72^. Single organoids were imaged on a light microscope and organoid diameter was measured as a readout of organoid size.

### Organoid metabolic profiling and nutrient tracing

For glucose tracer analysis experiments, 17.5 mM [U-^13^C]glucose (Cambridge Isotope Laboratories) was added to glucose-free SILAC Advanced DMEM/F-12 Flex Media (Fisher Scientific). Arginine, lysine and alanine were also added back to the SILAC base media at the same concentrations found in Advanced DMEM/F-12 (Fisher Scientific). Organoids were grown in mouse organoid media made with the SILAC base media. For lactate tracer analysis experiments, organoids were cultured with 20 mM [U-^13^C]Lactate (Cambridge Isotope Laboratories, CLM-1579-0.5) for 24 h before metabolite extraction. To extract metabolites, tracer-containing medium was aspirated. Organoids were repeatedly blasted with cold 150 mM ammonium acetate pH 7.3 using a P-1000 pipette until the Matrigel ring was dislodged. The suspension was transferred to an Eppendorf tube and centrifuged at 800*g* for 5 min at 1 °C. The supernatant was aspirated and 500 μl of cold 80% methanol was added to the organoid pellet. We added 10 μl of 1 mM norvaline (Sigma) as an internal standard. Each sample was vortexed for 30 s and centrifuged at 17,000*g* for 5 min at 1 °C. We transferred 420 μl of the supernatant to an ABC vial (Fisher Scientific) and evaporated using an EZ-2Elite evaporator (Genevac). Samples were stored at −80 °C before analysis.

Dried metabolites were resuspended in 50% acetonitrile:water and one-tenth was loaded onto a Luna 3μm NH2 100A (150 × 2.0 mm^2^) column (Phenomenex). The chromatographic separation was performed on a Vanquish Flex (Thermo Scientific) with mobile phases A (5 mM NH_4_AcO pH 9.9) and B (acetonitrile) and a flow rate of 200 μl min^−1^. A linear gradient from 15% A to 95% A over 18 min was followed by 9 min of isocratic flow at 95% A and re-equilibration to 15% A. Metabolites were detected with a Thermo Scientific Q Exactive mass spectrometer run with polarity switching (+3.5 kV/−3.5 kV) in full scan mode with an *m*/*z* range of 70–975 and 70,000 resolution. TraceFinder 4.1 (Thermo Scientific) was used to quantify the targeted metabolites by area under the curve using expected retention time and accurate mass measurements (<5 ppm). For labelled datasets, relative amounts of metabolites were calculated by summing up the values for all isotopologues of a given metabolite. Metabolite isotopologue distributions were corrected for natural C-13 abundance.

Normalization was performed by resuspending the cell pellet in 300 μl of lysis solution (0.1 M NaCl, 20 mM Tris-HCl, 0.1% SDS, 5 mM EDTA, 500 μg ml^−1^ Proteinase K (Fisher Scientific) in distilled water). Samples were syringed with a 25 G needle to reduce viscosity and 50 μl of each sample was transferred to a 96-well black-wall, clear-bottom tissue culture plate (Corning). We added 50 μl of lysis solution to one well for a blank reading. Then, 100 μl of 5 μg ml^−1^ Hoechst 33342 (Invitrogen) in distilled water was added to each well and 96-well plates were incubated for 30 min in the dark at 37 °C before measurement of DNA-based florescence using a Tecan Infinite M1000 plate reader with 355-nm excitation and 465-nm emission. The blank reading was subtracted from each absorbance value to calculate relative cell amount.

### PCA

PCA was performed using metabolite abundance and fractional contribution data in the Python programming language (v.3.9.12). Data were processed using the NumPy (v.1.22.4), pandas (v.1.4.2) and scikit-learn (v.1.0.2) libraries and visualized using the Matplotlib library (v.3.5.1). Feature scaling was done along the metabolite dimension using the StandardScaler class from scikit-learn which employs *z*-score normalization. The 95% confidence ellipses were generated with a script provided by Matplotlib.

### Intracellular flow cytometry

Organoids were removed from Matrigel by incubating in Advanced DMEM/F-12 (Gibco) containing 1 mg ml^−1^ dispase (Gibco) and 10 μM RI for 1 h at 37 °C. After centrifugation at 800*g* for 5 min, the pellet was washed with 1 × PBS. Organoids were resuspended in 800 μl of 0.05% Trypsin-EDTA (Gibco) and incubated at 37 °C for 5 min. The trypsin was quenched with 200 μl of RPMI, 10% FBS, 1% P/S + RI and organoids were pipetted up and down ten times to dissociate to single cells and passed through a 100-μm cell strainer (Corning). Dissociated cells from mouse prostate organoids were washed with PBS and fixed in 1 ml of 2% paraformaldehyde made from 16% paraformaldehyde (Electron Microscopy Sciences) in PBS for 15 min on ice. For experiments including EpCAM surface staining, cells were stained with EpCAM-APC/Cy7 (BioLegend 118218, 1:100) in RPMI, 10% FBS, 1% P/S + RI for 15 min before fixation. Cells were then washed with PBS and permeabilized in 1 ml of permeabilization buffer (0.1% Saponin (Sigma-Aldrich), 5% FBS (Corning) in PBS) for 15 min at room temperature in the dark. Cells were resuspended in 100 ml of permeabilization buffer and stained with rabbit anti-cytokeratin 5-Alexa Fluor 647 (Abcam Ab193895, 1:100) and rabbit anti-cytokeratin 8-Alexa Fluor 488 (Abcam Ab192467, 1:100) for 20 min at room temperature in the dark. Cells were washed with permeabilization buffer and resuspended in PBS for analysis on a BD FACS Canto (BD Biosciences).

### Organoid immunofluorescence

Organoids were removed from Matrigel by incubating in Advanced DMEM/F-12 (Gibco) containing 1 mg ml^−1^ dispase (Gibco) and 10 μM RI for 1 h at 37 °C. After centrifugation at 800*g* for 5 min, the pellet was washed with 1 × PBS three times. Organoids were then fixed in 4% paraformaldehyde in PBS for 15 min. After fixation, organoids were washed with PBS three times. Organoids were then blocked in 2% donkey serum in 0.25% Triton X-100 for 1 h. Organoids were washed once with PBS and stained with anti-Cytokeratin 8 (Biolegend 904804, 1:500) antibody and anti-p63 (Biolegend 619002, 1:500) antibody in 0.5% BSA, 0.25% Triton X-100 at 4 °C overnight. Organoids were then washed with PBS three times, with the last wash lasting 6 h. Secondary antibody staining was performed overnight at 4 °C using goat anti-rabbit IgG-AlexaFluor647 (Thermo Fisher 21245, 1:1,000) and goat anti-mouse IgG-AlexaFluor488 (Thermo Fisher 11001, 1:1,000) in 0.5% BSA, 0.25% Triton X-100 with one drop of NucBlue. Organoids were washed with PBS three times and placed in PBS + 0.1% Tween-20 until imaging on a Nikon Ti-E Fluorescence Motorized DIC Microscope (Nikon) with RCM1 confocal box (Confocal.nl) using Nikon NIS Elements Imaging Software and Nikon CFI Apo LWD Lambda S 20XC WI objective, material number MRD77200.

### Cell lines

Cell lines were routinely tested for mycoplasma and authentication by short tandem repeat analysis (Laragen). Tissue culture plates were coated with 0.01% (v/v) poly-l-lysine (Sigma, P4832) diluted 1/20 in distilled water and washed with PBS to enhance cell attachment. 16D cells were received from Dr Amina Zoubeidi and cultured in RPMI base media (Gibco) + 10% FBS (v/v) + 1 × P/S. LuCaP35 cells were received from Dr Eva Corey and Dr Peter Nelson and cultured in DMEM base media (Gibco, 11965-092) + 10% FBS (v/v) + 1 × P/S and 1 × GlutaMAX. LAPC4 cells were received from Dr Rob Reiter and cultured in IMDM (Gibco, 31980-030) + 5% FBS (v/v) + 1 × P/S. UK5099 treatment was performed by adding 10 μM or 30 μM UK5099 (Sigma, PZ0160) every 48 h.

### Histone extractions

Histone extractions were performed using a histone extraction kit (Abcam, Ab113476) according to manufacturer instructions.

### ATAC-seq

Cells were collected and frozen in culture media containing FBS and 5% dimethylsulfoxide. Cryopreserved cells were sent to Active Motif to perform the ATAC-seq assay. The cells were then thawed in a 37 °C water bath, pelleted, washed with cold PBS and tagmented as previously described^73^, with some modifications^74^. Briefly, cell pellets were resuspended in lysis buffer, pelleted and tagmented using the enzyme and buffer provided in the Nextera Library Prep Kit (Illumina). Tagmented DNA was then purified using the MinElute PCR purification kit (Qiagen), amplified with ten cycles of PCR and purified using Agencourt AMPure SPRI beads (Beckman Coulter). The resulting material was quantified using the KAPA Library Quantification Kit for Illumina platforms (KAPA Biosystems) and sequenced with PE42 sequencing on the NovaSeq 6000 sequencer (Illumina). Reads were aligned using the BWA algorithm (v.0.7.12; mem mode; default settings). Duplicate reads were removed; only reads mapping as matched pairs and uniquely mapped reads (mapping quality ≥ 1) were used for further analysis. Alignments were extended in silico at their 3′-ends to a length of 200 bp and assigned to bins 32 nucleotides in size along the genome. The resulting histograms (genomic ‘signal maps’) were stored in bigWig files. Peaks were identified using the MACS 2.1.0 algorithm at a cutoff of *P* value 1 × 10^−7^, without control file, and with the –nomodel option. Peaks that were on the ENCODE blacklist of known false ChIP–seq peaks were removed. Signal maps and peak locations were used as input data to Active Motif’s proprietary analysis program, which creates Excel tables containing detailed information on sample comparison, peak metrics, peak locations and gene annotations. For differential analysis, reads were counted in all merged peak regions (using Subread), and the replicates for each condition were compared using DESeq2 (v.1.24.0)^54^.

### HOMER transcription factor motif analysis

#### Identification of sites with differential ATAC-seq signal

After identifying merged regions as part of the standard analysis pipeline, the DESeq2 software was run on the unnormalized BAM files (without duplicates). In brief, the DESeq2 software generates normalized counts specifically for the merged regions, and the shrunken log_2_ fold change and adjusted *P* values for each merged region are calculated. For the subsequent steps of the analysis, we consider any region as differential if the adjusted *P* value is less than 0.1.

#### HOMER-based motif analysis

BED files listing the significantly increased (‘DESeq2_Up_difpeaks.bed’) and decreased (‘DESeq2_Down_difpeaks.bed’) regions were generated for each comparison. Each BED file was then sorted by the shrunken log_2_ fold change and the 2,500 regions with the largest absolute fold changes were selected. We then performed HOMER motif analysis (findMotifsGenome.pl) on the 200-bp sequence centred around the midpoint of the differential region (+100 bp, −100 bp). During this analysis, common repeats are masked as this can affect the discovery of de novo motifs. The analysis identifies motifs that are enriched across all sequences; individual peak regions are not annotated with specific motifs.

### PDX enzalutamide sensitivity assay

Using a razor blade, MDA PCa 203-A PDX and MDA PCa 183-A PDX tumours were mechanically dissociated in dissociation media composed of RPMI 1640 (Gibco) containing 10% FBS (Corning), 1 × P/S, 1 mg ml^−1^ collagenase type I (Gibco), 1 mg ml^−1^ dispase (Gibco), 0.1 mg ml^−1^ deoxyribonuclease (Gibco) and 10 μM RI. When large chunks were no longer visible, the samples were incubated at 37 °C on a nutating platform for 15 min in 20 ml of dissociation media. After centrifugation at 800*g* for 5 min, the pellet was washed with 1 × PBS (Gibco). The cell pellet was resuspended in Advanced DMEM/F-12 and passed through a 100-μm cell strainer (Corning). After centrifugation at 800*g* for 5 min, the pellet was resuspended in human organoid media and plated in 75% growth factor reduced Matrigel (Corning) based on established protocols^66^. After 7 d of culture with vehicle, 10 μM UK5099 (Sigma, PZ0160) or 20 mM sodium lactate (Sigma, L7022-5G), organoids were removed from Matrigel by incubating in Advanced DMEM/F-12 containing 1 mg ml^−1^ dispase and 10 μM of the p160ROCK inhibitor Y-27632 dihydrochloride for 1 h at 37 °C. After centrifugation at 800*g* for 5 min, the pellet was washed with 1 × PBS. Organoids were then plated into rings in a 96-well black-wall, clear-bottom plate (Fisher, 07-200-588) in 75% growth factor reduced Matrigel with or without 10 μM enzalutamide (Selleck Chemicals, S1250). After 5 d of culture, a CellTiter-Glo assay (Promega, G7571) was performed according to manufacturer instructions and relative luminescence signal was quantified on a Tecan Infinite M1000 plate reader.

### Cell line enzalutamide sensitivity assays

After 7 d of culture with vehicle, 10 μM UK5099 (Sigma, PZ0160), 30 μM UK5099 or 20 mM sodium lactate (Sigma, L7022-5G), cells were plated into a 96-well black-wall, clear-bottom plate (Fisher, 07-200-588) with or without 10 μM enzalutamide (Selleck Chemicals, S1250). The 96-well plate was coated with 0.01% (v/v) poly-l-lysine (Sigma, P4832) diluted 1/20 in distilled water and washed with PBS before plating cells to enhance cell attachment. After 2 d of culture, a CellTiter-Glo assay (Promega, G7571) was performed according to manufacturer instructions and relative luminescence signal was quantified on a Tecan Infinite M1000 plate reader. For the 5-ethynyl-2′-deoxyuridine-based (EdU) cell cycle assay, cells were seeded at 30% confluence and cultured in 6-well dishes for 72 h before cell cycle analysis. Media changes were performed 48 h after plating. After 72 h of culture, cell cycle analysis was performed using an EdU kit (Thermo Fisher Scientific, C10635) according to the specified protocol. EdU labelling was performed for 2 h. For experiments that contained small-molecule inhibitors, fresh inhibitor(s) were adding during each media change. Flow cytometry analysis identified the percentage EdU-positive.

### Statistics and reproducibility

Prism v.8.3.0 (GraphPad) was used to generate graphs and perform statistical analyses. All in vitro experiments shown were repeated at least three times with similar results obtained, and representative data are shown unless otherwise indicated. No statistical method was used to predetermine sample size but our sample sizes are similar to those reported in previous publications^21^. Data distribution was assumed to be normal but this was not formally tested. No data were excluded from the analyses. Data collection and analysis were not performed blind to the conditions of the experiments. For animal experiments, mice were randomly divided into cages. For in vitro experiments, samples were not randomized as this was not relevant for the individual assays.

### Reporting summary

Further information on research design is available in the Nature Portfolio Reporting Summary linked to this article.

## Online content

Any methods, additional references, Nature Portfolio reporting summaries, source data, extended data, supplementary information, acknowledgements, peer review information; details of author contributions and competing interests; and statements of data and code availability are available at 10.1038/s41556-023-01274-x.

### Supplementary information

Reporting Summary

Supplementary Tables Supplementary Tables 1–10.

### Source data

Source Data Figs. 1–6 and Extended Data Figs. 1–8 and 10 All statistical source data.

Source Data Figs. 2–5 and Extended Data Figs. 1, 3, 4, 6, 7 and 10 All unprocessed western blots.

## Data Availability

Bulk RNA-seq, scRNA-seq and ATAC-seq data that support the findings of this study have been deposited in the Gene Expression Omnibus (GEO) under accession codes GSE221023, GSE222786, GSE236573, GSE206555 and GSE221442. Previously published RNA-seq data that were re-analysed here are available under accession codes GSE122367 and GSE67070. The SMMU, Beltran et al. and TCGA datasets were accessed on cBioPortal (https://www.cbioportal.org/). Ensembl databases were accessed from http://useast.ensembl.org/Mus_musculus/Info/Index. An interactive scRNA-seq *t*-SNE plot is available at: https://singlecell.broadinstitute.org/single_cell/study/SCP1234/prostate-organoid-vehicle-uk5099. Source data are provided with this paper. All other data supporting the findings of this study are available from the corresponding author on reasonable request.

## Code availability

The code used to generate the PCA plots can be accessed at https://github.com/Nick-Nunley/Metabolism-and-lineage-PCA.git.
