# Supplementary material for: Prostate lineage-specific metabolism governs luminal differentiation and response to antiandrogen treatment
Source: Nat Cell Biol. 2023 Dec 4;25(12):1821–32. doi: 10.1038/s41556-023-01274-x (PMC10709144; doi:10.1038/s41556-023-01274-x)
Supplement: Supplementary file 1 — Reporting Summary [file 41556_2023_1274_MOESM1_ESM.pdf]

nature portfolio

Corresponding author(s): Andrew S. Goldstein

Last updated by author(s): Sep 13, 2023

Reporting Summary

Nature Portfolio wishes to improve the reproducibility of the work that we publish. This form provides structure for consistency and transparency in reporting. For further information on Nature Portfolio policies, see our Editorial Policies and the Editorial Policy Checklist.

Statistics

For all statistical analyses, confirm that the following items are present in the figure legend, table legend, main text, or Methods section.

n/a	Confirmed	
☐	☒	The exact sample size (*n*) for each experimental group/condition, given as a discrete number and unit of measurement
☐	☒	A statement on whether measurements were taken from distinct samples or whether the same sample was measured repeatedly
☐	☒	The statistical test(s) used AND whether they are one- or two-sided<br>*Only common tests should be described solely by name; describe more complex techniques in the Methods section.*
☒	☐	A description of all covariates tested
☐	☒	A description of any assumptions or corrections, such as tests of normality and adjustment for multiple comparisons
☐	☒	A full description of the statistical parameters including central tendency (e.g. means) or other basic estimates (e.g. regression coefficient) AND variation (e.g. standard deviation) or associated estimates of uncertainty (e.g. confidence intervals)
☐	☒	For null hypothesis testing, the test statistic (e.g. *F*, *t*, *r*) with confidence intervals, effect sizes, degrees of freedom and *P* value noted<br>*Give P values as exact values whenever suitable.*
☒	☐	For Bayesian analysis, information on the choice of priors and Markov chain Monte Carlo settings
☒	☐	For hierarchical and complex designs, identification of the appropriate level for tests and full reporting of outcomes
☐	☒	Estimates of effect sizes (e.g. Cohen's *d*, Pearson's *r*), indicating how they were calculated

Our web collection on statistics for biologists contains articles on many of the points above.

Software and code

Policy information about availability of computer code

Data collection

For bulk RNA-seq, RNA was extracted from the cells using the RNeasy Mini Kit (QIAGEN) following the manufacturer's instruction. Libraries for RNA-seq were prepared with KAPA Stranded mRNA-Seq Kit (Roche). The workflow consists of mRNA enrichment, cDNA generation, and end repair to generate blunt ends, A-tailing, adaptor ligation and PCR amplification. Different adaptors were used for multiplexing samples in one lane. Sequencing was performed on Illumina HiSeq 3000. For single cell sequencing, samples were counted using Countess II Automated Cell Counter (Thermo Fisher Scientific) and hemocytometer for cell concentration and viability using Trypan Blue stain 0.4% (Invitrogen). Cells were loaded to form GEMs and barcode individual cells. GEMs were treated according to manufacturer's instructions. Single cell gene expression libraries were created using Chromium Next GEM Single Cell 3' (v3.1 Chemistry) (10x Genomics), Chromium Next GEM Chip G Single Cell Kit (10x Genomics), and Single Index Kit T Set A (10x Genomics) according to the manufacturer's instructions. Paired-end sequencing was done using Illumina Novaseq 6000.

For ATAC-seq, cell pellets were resuspended in lysis buffer, pelleted, and tagmented using the enzyme and buffer provided in the Nextera Library Prep Kit (Illumina). Tagmented DNA was then purified using the MinElute PCR purification kit (Qiagen), amplified with 10 cycles of PCR, and purified using Agencourt AMPure SPRI beads (Beckman Coulter). Resulting material was quantified using the KAPA Library Quantification Kit for Illumina platforms (KAPA Biosystems) and sequenced with PE42 sequencing on the NovaSeq 6000 sequencer (Illumina).

For metabolomics on primary cells, cells were cultured overnight in mouse organoid media containing [U-13C]glucose (Cambridge Isotope Laboratories). Prior to metabolite extraction, tracer-containing media was aspirated and cells were washed with cold 150mM ammonium acetate pH 7.3. Metabolite extractions were performed by adding 500 µL cold 80% methanol to each well and removing cells using a cell scrapper. The cell suspension was transferred to an Eppendorf tube and 10 µL 1mM norvaline (Sigma) was added as an internal standard. Each sample was vortexed for 30 seconds and centrifuged at 17000g for 5 minutes at 1°C. 420 µL of the supernatant was transferred to an ABC vial (Fisher Scientific) and evaporated using an EZ-2Elite evaporator (Genevac). Samples were stored at -80°C prior to analysis. The LC

separation utilizing an Ion Chromatography System (ICS) 5000 (Thermo Scientific) was performed on a Dionex IonPac AS11-HC-4µm anion exchange column. The gradient was 5-95 mM KOH over 13 minutes, followed by 5 minutes at 95mM, before re-equilibration to 5mM. Other LC parameters: flow rate 350 µl/min, column temperature 35°C, injection volume 5 µL. The Q Exactive mass spectrometer (Thermo Scientific) was operated in negative ion mode for detection of metabolites using a resolution of 70,000 at m/z 200 and a scan range of 70-900 m/z.For metabolomics on organoids, For glucose tracer analysis experiments, 17.5mM [U-13C]glucose (Cambridge Isotope Laboratories) was added to glucose-free SILAC Advanced DMEM/F-12 Flex Media (Fisher Scientific). Arginine, lysine and alanine were also added back to the SILAC base media at the same concentrations found in Advanced DMEM/F-12 (Fisher Scientific). Organoids were grown in mouse organoid media made with the SILAC base media. For lactate tracer analysis experiments, organoids were cultured with 20mM [U-13C]Lactate (Cambridge Isotope Laboratories, CLM-1579-0.5) for 24 hours prior to metabolite extraction. To extract metabolites, tracer-containing media was aspirated. Organoids were repeatedly blasted with cold 150mM ammonium acetate pH 7.3 using a P-1000 pipette until the Matrigel ring was dislodged. The suspension was transferred to an Eppendorf tube and centrifuged at 800g for 5 minutes at 1°C. The supernatant was aspirated and 500µL cold 80% methanol was added to the organoid pellet. 10µL 1mM norvaline (Sigma) was added as an internal standard. Each sample was vortexed for 30 seconds and centrifuged at 17000g for 5 minutes at 1°C. 420µL of the supernatant was transferred to an ABC vial (Fisher Scientific) and evaporated using an EZ-2Elite evaporator (Genevac). Samples were stored at -80°C prior to analysis. Dried metabolites were resuspended in 50% ACN:water and 1/10th was loaded onto a Luna 3um NH2 100A (150 × 2.0 mm) column (Phenomenex). The chromatographic separation was performed on a Vanquish Flex (Thermo Scientific) with mobile phases A (5 mM NH_4AcO pH 9.9) and B (ACN) and a flow rate of 200 µL/minute. A linear gradient from 15% A to 95% A over 18 minutes was followed by 9 minutes isocratic flow at 95% A and reequilibration to 15% A. Metabolites were detection with a Thermo Scientific Q Exactive mass spectrometer run with polarity switching (+3.5 kV/− 3.5 kV) in full scan mode with an m/z range of 70-975 and 70.000 resolution. The LC separation utilizing an Ion Chromatography System (ICS) 5000 (Thermo Scientific) was performed on a Dionex IonPac AS11-HC-4µm anion exchange column. The gradient was 5-95 mM KOH over 13 minutes, followed by 5 minutes at 95mM, before re-equilibration to 5mM. Other LC parameters: flow rate 350 µl/min, column temperature 35°C, injection volume 5 µL. The Q Exactive mass spectrometer (Thermo Scientific) was operated in negative ion mode for detection of metabolites using a resolution of 70,000 at m/z 200 and a scan range of 70-900 m/z.

For western blot readouts, each sample was sonicated for 40 seconds at 20kHz with a sonic dismembrator (Fisher Scientific) to improve membranous and nuclear protein yield. Samples were run on NuPAGE 4–12% Bis-Tris Gel (Invitrogen) and transferred onto PVDF membranes (Millipore Sigma). Total protein was visualized using SYPRO RUBY protein blot stain (Fisher Scientific) and membranes were blocked in PBS + 0.1% Tween-20 (Fisher Scientific) + 5% milk (Fisher Scientific). Proteins were probed with primary antibodies followed by chromophore-conjugated anti-mouse (Invitrogen, A21235) or anti-rabbit secondary antibodies (Invitrogen, A21244) or HRP-conjugated anti-mouse (Thermo, 31430) or anti-rabbit secondary antibodies (Thermo, 31463) and detected by florescence or HRP chemiluminescence respectively. Samples were acquired using the Invitrogen iBright FL1500 imaging system.

For immunofluorescence, organoids were fixed in 4% paraformaldehyde (PFA) in PBS for 15 minutes. After fixation, organoids were washed with PBS three times. Organoids were then blocked in 2% donkey serum in 0.25% Triton X-100 for 1 hour. Organoids were washed once with PBS and stained with anti-Cytokeratin 8 (Biolegend, 904804, 1:500) antibody and anti-p63 (Biolegend, 619002, 1:500) antibody in 0.5% BSA 0.25% Triton X-100 at 4°C overnight. Organoids were then washed with PBS three times, with the last wash lasting six hours. Secondary antibody staining was performed overnight at 4°C using goat anti-rabbit IgG-AlexaFluor647 (1:1000) and goat anti-mouse IgG-AlexaFluor488 (1:1000) in 0.5% BSA 0.25% Triton X-100 with one drop of NucBlue. Organoids were washed with PBS three times and placed in PBS + 0.1% Tween-20 until imaging on a confocal microscope.

Flow cytometry was performed using a BD LSRFortessa and BD FACS Canto with FACSDiva software (8.0.2). Dissociated cells from mouse prostate organoids were washed with PBS and fixed in 1mL of 2% paraformaldehyde made from 16% paraformaldehyde (Electron Microscopy Sciences) in PBS for 15 minutes on ice. For experiments including EpCAM surface staining, cells were stained with EpCAM-APC/Cy7 (Abcam) in RPMI 10% FBS 1% P/S + ROCK inhibitor for 15 minutes prior to fixation. Cells were then washed with PBS and permeabilized in 1 mL of permeabilization buffer (0.1% Saponin (Sigma-Aldrich), 5% FBS (Corning) in PBS) for 15 minutes at room temperature in the dark. Cells were resuspended in 100 mL of permeabilization buffer and stained with rabbit anti-cytokeratin 5-Alexa Fluor 647 (Abcam) and rabbit anti-cytokeratin 8-Alexa Fluor 488 (Abcam) for 20 minutes at room temperature in the dark. Cells were washed with permeabilization buffer and resuspended in PBS for analysis.

For immunofluorescence, organoids were imaged on a Nikon Ti-E Fluorescence Motorized DIC Microscope (Nikon) with RCM1 confocal box (Confocal.nl) using Nikon NIS Elements Imaging Software and Nikon CFI Apo LWD Lambda S 20XC WI objective, material number MRD77200.

Data analysis

For Bulk RNA sequencing, data quality check was done on Illumina SAV. Demultiplexing was performed with Illumina Bcl2fastq v2.19.1.403 software. The reads were mapped by STAR 2.7.9a and read counts per gene were quantified using the mouse Ensembl GRCm39.105 GTF file. In Partek Flow v7.0, read counts were normalized by CPM 1.0 x 10-4. All results of differential expression analysis utilized the statistical analysis tool, DESeq2 (v1.40.2). KEGG pathway analysis was performed using DAVID Bioinformatics. GSEA was performed using GSEA_4.0.3 software. For significance testing relevant to Extended Data Figure 8: To measure the RNA abundance, RNA-seq reads were trimmed using fastp (v0.20.1) with default parameters, then mapped to the mouse Ensembl GRCm38-EBI102 using STAR (v2.7.10a). STAR alignment was carried out using default settings with an additional argument to include the minimum length of 10 bp for chimeric junction segment. Aligned reads were quantified using rsem-calculate-expression program (v1.3.3) for TPM calculation with default settings. We also performed read level quality control metrics using FastQC (v0.11.8).

To test the combination effect of UK5099 and Butyrate, we constructed the following two-factor, two-level linear model:

Y ~ α_0 + α_1 · UK5099 + α_2 · Butyrate + α_3 · UK5099:Butyrate

Here, Y refers to the abundance level of a gene, which is log2 transformation of TPM values; α_0 refers to the basal abundance level of that gene; UK5099 indicates "UK5099-dependent, Butyrate-independent" abundance changes; Butyrate indicates "Butyrate-dependent, UK5099-independent" abundance changes; UK5099-Butyrate captures "UK5099-dependent, Butyrate-dependent" abundance changes.

We used R package limma (v3.17) in R (v4.2.2) to fit each gene in the RNA sequencing to the model. The model was adjusted using empirical Bayes moderation for standard error, and the false discovery rate (FDR) was controlled using the Benjamini-Hochberg method. Genes exhibiting significant changes were identified based on the adjusted p-value < 0.01 and |log2(Coefficient)| > 1 threshold. Venn diagrams representing the overall and directional effects were generated using the VennDiagram package in R (v1.7.3). The hierarchical clustering heatmap of gene TPM was constructed using R package BoutrosLab.plotting.general (v7.0.8).

Functional enrichment analysis: For genes with differential mRNA abundance calculated based on the coefficient from the general linear model, we ranked the genes according to their log2(Coefficient) from high to low. Gene set enrichment analysis was then performed using the R package clusterProfiler (v3.17). Gene ontology enrichment analysis was conducted for both up-regulated genes (log2(Coefficient) > 1, -log10(FDR) > 1) and down-regulated genes (log2(Coefficient) < -1, -log10(FDR) > 1) using R package clusterProfiler (v3.17). The results of both GSEA and GO enrichment analyses were visualized using the BoutrosLab.plotting.general (v7.0.8).

For single cell RNA sequencing, basecalling was done using Illumina Casava (v1.7) software. CellRanger (v5.1) count was used to create an RNA abundance matrix and samples were loaded into the Seurat (v3.2.2) R package. DoubletDcon (v1.1.2) was used to remove potential doublets. Additionally, cells were filtered based on the number of genes (≥250), unique molecular identifiers (UMIs; ≥500) and percent of mitochondrial genes (<20%). After quality control, log2 normalization was performed within each sample using NormalizeData function with default parameters. The top 2,000 variable genes were selected using FindVariableFeatures. The two samples were integrated together with FindIntegrationAnchors and IntegrateData functions which incorporate canonical correlation analysis to align cells with similar transcriptomic patterns across samples. Principal component analysis (PCA) was performed after the integration. The top 20 PCs were used to construct the k-nearest neighbor graph, followed by Louvain algorithm to cluster cells based on similar gene expression patterns. Cell clusters were visualized using t-distributed stochastic neighbor embedding (tSNE). After, markers for each cluster were determined using FindAllMarkers with average log2 fold change > 0.25 and minimum percent difference > 0.25. Cell types were determined by comparing canonical markers with cluster-specific markers. After cell type identification, cell type proportions were calculated with the number of cells in each cell type divided by the total number of cells in each sample. To see the effect of UK5099 in the luminal cluster, DotPlot in Seurat was used to visualize the expression of luminal markers, basal markers, glycolytic enzymes, lipid metabolism genes, and inflammatory signaling genes.

Metabolomics data were extracted using Tracefinder 3.1 and 4.1 (Thermo Scientific). Metabolites were identified based on accurate mass (±5 ppm) and previously established retention times of pure standards. For labeled datasets, relative amounts of metabolites were calculated by summing up the values for all isotopologues of a given metabolite. Metabolite Isotopologue Distributions were corrected for natural C13 abundance.

Principal component analysis was processed using the NumPy (v1.22.4), pandas (v1.4.2), and scikit-learn (v1.0.2) libraries and visualized using the Matplotlib library (v3.5.1). The code used to generate the PCA plots can be accessed at https://github.com/Nick-Nunley/Metabolism-and-lineage-PCA.git

For ATAC-seq, Reads were aligned using the BWA algorithm (v0.7.12; mem mode; default settings). Peaks were identified using the MACS 2.1.0 algorithm. Signal maps and peak locations were used as input data to Active Motif's proprietary analysis program. For differential analysis, reads were counted in all merged peak regions (using Subread), and the replicates for each condition were compared using DESeq2 (v1.24.0). HOMER motif analysis (findMotifsGenome.pl) identifies motifs that are enriched across all sequences.
Prism v8.3.0 (GraphPad) was used to generate graphs and perform statistical analyses.

For manuscripts utilizing custom algorithms or software that are central to the research but not yet described in published literature, software must be made available to editors and reviewers. We strongly encourage code deposition in a community repository (e.g. GitHub). See the Nature Portfolio guidelines for submitting code & software for further information.

Data

Policy information about availability of data

All manuscripts must include a data availability statement. This statement should provide the following information, where applicable:

- Accession codes, unique identifiers, or web links for publicly available datasets
- A description of any restrictions on data availability
- For clinical datasets or third party data, please ensure that the statement adheres to our policy

Bulk RNA-seq, scRNA-seq and ATAC-seq data that support the findings of this study have been deposited in the Gene Expression Omnibus (GEO) under accession codes GSE221023, GSE222786, GSE236573, GSE206555 and GSE221442. Previously published RNA-seq data that were re-analyzed here are available under accession codes GSE122367 and GSE67070. The SMMU, Beltran et al., and TCGA datasets were accessed on cBioPortal (https://www.cbioportal.org/). Ensembl database were accessed from http://useast.ensembl.org/Mus_musculus/Info/Index. An interactive scRNA-seq tSNE plot is available at: https://singlecell.broadinstitute.org/single_cell/study/SCP1234/prostate-organoid-vehicle-uk5099. All other data supporting the findings of this study are available from the corresponding author on reasonable request.

Human research participants

Policy information about studies involving human research participants and Sex and Gender in Research.

Reporting on sex and gender	MDA-PCa 183-A was derived from a 58-year-old male. MDA-PCa 203-A was derived from a 58-year-old male.
Population characteristics	Not applicable
Recruitment	Written informed consent was obtained from patients before sample acquisition. Patients were not compensated, and they cannot be identified from data provided in this manuscript.
Ethics oversight	All samples were processed according to a protocol approved by the Institutional Review Board of the University of Texas MD Anderson Cancer Center. The studies were conducted in accordance with the Belmont Report and the US Common Rule.

Note that full information on the approval of the study protocol must also be provided in the manuscript.

Field-specific reporting

Please select the one below that is the best fit for your research. If you are not sure, read the appropriate sections before making your selection.

☒ Life sciences ☐ Behavioural & social sciences ☐ Ecological, evolutionary & environmental sciences

For a reference copy of the document with all sections, see nature.com/documents/nr-reporting-summary-flat.pdf

Life sciences study design

All studies must disclose on these points even when the disclosure is negative.

Sample size	Sample size was not pre-determined in this study but our sample sizes are similar to those reported in previous publications (Crowell et al, 2019). Each experiment included at least 2-3 technical and/or biological replicates, consistent with the standard for publications in our field. Statistical tests were used to confirm that sample sizes were sufficient to support conclusions in this study. Results confirmed small variability and a high degree of reproducibility between samples and independent experiments.
Data exclusions	No data were excluded from the analyses in this study.
Replication	Biological replicates were performed three times and confirmed the reproducibility of all experiments described in the manuscript.
Randomization	For animal treatment experiments, mice were divided randomly into cages. For in vitro experiments, samples were not randomized as this was not relevant for the individual assays.
Blinding	Blinding was not performed because the researchers performing experiments were also collecting data. When possible, blinded researchers measured organoid number and size to reduce potential bias.

Reporting for specific materials, systems and methods

We require information from authors about some types of materials, experimental systems and methods used in many studies. Here, indicate whether each material, system or method listed is relevant to your study. If you are not sure if a list item applies to your research, read the appropriate section before selecting a response.

Materials & experimental systems

n/a	Involved in the study	
☐	☒	Antibodies
☐	☒	Eukaryotic cell lines
☒	☐	Palaeontology and archaeology
☐	☒	Animals and other organisms
☒	☐	Clinical data
☒	☐	Dual use research of concern

Methods

n/a	Involved in the study	
☒	☐	ChIP-seq
☐	☒	Flow cytometry
☒	☐	MRI-based neuroimaging

Antibodies

Antibodies used

Flow cytometry: rat anti-CD49f-PE (BioLegend 313612, 1:100), rat anti-CD326 (EpCAM)-APC (BioLegend 324207, 1:100), rat anti-CD31-FITC (BioLegend 102405, 1:100), rat anti-CD45-FITC (BioLegend 103108, 1:100), rat anti-Ter119-FITC (BioLegend 116205, 1:100) and rat anti-ESAM-FITC (BioLegend 136205, 1:100).

Intracellular flow cytometry: rabbit anti-cytokeratin 5-Alexa Fluor 647 (Abcam Ab193895, 1:100) and rabbit anti-cytokeratin 8-Alexa Fluor 488 (Abcam Ab192467, 1:100).

Western blotting: Proteins were probed with primary antibodies followed by chromophore-conjugated anti-mouse (Invitrogen A21235, 1:1000) or anti-rabbit secondary antibodies (Invitrogen A21244, 1:1000) or HRP-conjugated anti-mouse (Thermo 31430, 1:10000) or anti-rabbit secondary antibodies (Thermo 31463, 1:10000) and detected by florescence or HRP chemiluminescence respectively. Primary antibodies used were anti-Cytokeratin 5 (Biolegend 905504, 1:3000), anti-Probasin (Santa Cruz sc-393830, 1:1000), anti-Glut1 (Abcam ab115730, 1:10,000), anti-Glut3 (Abcam ab191071, 1:1000), anti-Hexokinase 2 (Cell Signaling 28675, 1:1000), anti- Phosphofructokinase (Abcam ab204131, 1:5000), anti-Pyruvate carboxylase (Abcam ab128952, 1:1000), anti- Pyruvate dehydrogenase E1 component subunit alpha (Proteintech 18068-1-AP, 1:1000), anti-Aconitase 2 (Abcam ab110321, 1:1000), anti-Histone H3 (Cell Signaling 9717S, 1:1000), anti-Cytokeratin 8 (Biolegend 904804, 1:1000), anti-p63 (Biolegend 619002, 1:1000), anti-beta Actin (Fisher MA1-140, 1:15000), anti-Proliferating cell nuclear antigen (Fisher 13-3900, 1:1000), anti-Androgen receptor (Abcam ab133273, 1:1000), anti-Mitochondrial pyruvate carrier 1 (Cell Signaling 14462, 1:1000), anti-Ki-67 (Abcam ab15580, 1:1000), anti-cleaved caspase-3 (Cell Signaling 9661L, 1:500), anti-Cytokeratin 18 (Fisher MA5-12104, 1:100), anti-Vinculin (Abcam Ab129002, 1:1000), anti-Phosphatase and tensin homolog (Cell Signaling 9559, 1:1000), anti-Retinoblastoma protein 1 (Abcam ab181616, 1:1000), anti-Acetyl-histone H3 (Lys9) (Cell Signaling 9649, 1:1000), anti-Pan-acetyl histone H3 (Active Motif 61637,

1:1000), anti-Histone H4 (Abcam ab10158, 1:1000), anti-Pan-acetyl histone H4 (Abcam ab177790, 1:1000), anti-prostate-specific antigen (Cell Signaling 5877, 1:1000), anti-neuron-specific enolase (Proteintech 66150-1-Ig, 1:3000), anti-synaptophysin (Cell Signaling 5461, 1:1000), anti-Sox2 (Cell Signaling 14962, 1:1000).

For immunofluorescence: Primary antibodies mouse anti-Cytokeratin 8 (Biolegend 904804, 1:500) antibody and rabbit anti-p63 (Biolegend 619002, 1:500). Secondary antibodies goat anti-rabbit IgG-AlexaFluor647 (1:1000) and goat anti-mouse IgG-AlexaFluor488 (1:1000).

Validation

All antibodies used in this study were purchased from commercial vendors who had validated specificity in human tissues/cells for the specific assays (flow cytometry, western blot, immunofluorescence).

Eukaryotic cell lines

Policy information about cell lines and Sex and Gender in Research

Cell line source(s)

For prostate cancer cell lines used in functional studies: 16D cells were received from Dr. Amina Zoubeidi. LuCaP35 cells were received from Dr. Eva Corey and Dr. Peter Nelson. LAPC4 cells were received from Dr. Rob Reiter. HEK293T cells were obtained from ATCC and used only to validate Cre activity, but were not used in any functional experiments.

Authentication

None of the prostate cancer cell lines were authenticated because they are not commercially available.

Mycoplasma contamination

Cell lines were routinely tested for mycoplasma and authentication by short tandem repeat analysis (Laragen).

Commonly misidentified lines
(See ICLAC register)

No commonly misidentified cell lines were used.

Animals and other research organisms

Policy information about studies involving animals; ARRIVE guidelines recommended for reporting animal research, and Sex and Gender in Research

Laboratory animals

Animals were housed under the care of the Division of Laboratory Animal Medicine at the University of California, Los Angeles, using protocols approved by the Animal Research Committee (ARC #2017-020). All mice are housed under 12 h:12 h light–dark cycle, with room temperature maintained at 73°F and relative humidity level of 30-70%. Mouse cages include clean bedding and enrichment materials consistent with Institutional Animal Care and Use Committee regulations. Prostates from 8-12 week old male C57BL/6J mice from Jackson Laboratories were used for primary basal and luminal cell experiments. Prostates used for isolation of multipotent basal cells and basal-derived luminal cells were collected from CD1 mice purchased from the Jackson Laboratory. The experimental mice used were males of mixed background and at P10-P12 age. Mpc1 floxed mice were male, 2-12 months of age, and of mixed C57Bl/6N and C57Bl/6J genetic background. Pten floxed and Pten;Rb1 floxed mice were male, aged 2-12 months, and of mixed C57BL/6:129/Sv:FVB genetic background. PDX was grown in male 2-12 month old NOD-scid-IL2R-gamma-null mice.

Wild animals

This study does not involve wild animals

Reporting on sex

All mice were male as prostate cancer only occurs in males and only male mice have prostate tissue.

Field-collected samples

This study does not involve field-collected samples

Ethics oversight

All experiments, including animal studies, were conducted in compliance with federal and state government guidelines and followed approved protocols by the Institutional Biosafety Committee and the Institutional Animal Care and Use Committee at the University of California, Los Angeles.

Note that full information on the approval of the study protocol must also be provided in the manuscript.

Flow Cytometry

Plots

Confirm that:

☒ The axis labels state the marker and fluorochrome used (e.g. CD4-FITC).

☒ The axis scales are clearly visible. Include numbers along axes only for bottom left plot of group (a 'group' is an analysis of identical markers).

☒ All plots are contour plots with outliers or pseudocolor plots.

☒ A numerical value for number of cells or percentage (with statistics) is provided.

Methodology

Sample preparation

Mouse prostate tissues were dissociated to single cells, stained with directly conjugated primary antibodies: rat anti-CD49f-PE (BioLegend), rat anti-CD326 (EpCAM)-APC (BioLegend), rat anti-CD31-FITC (BioLegend), rat anti-CD45-FITC (BioLegend), rat anti-Ter119-FITC (BioLegend) and rat anti-ESAM-FITC (BioLegend) for 20 minutes on ice. Cells were stained in media containing RPMI 1640 (Gibco), 10% FBS (Corning), 1x penicillin-streptomycin (Gibco), and 10μM of the p160ROCK inhibitor

Y-27632 dihydrochloride (Tocris Bioscience). For intracellular flow cytometry on organoids, Organoids were removed from Matrigel by incubating in Advanced DMEM/F-12 (Gibco) containing 1 mg/mL dispase (Gibco) and 10µM of the p160ROCK inhibitor Y-27632 dihydrochloride (Tocris Biosciences) for 1 hour at 37°C. After centrifugation at 800g for 5 minutes, the pellet was washed with 1x phosphate buffered saline (PBS). Organoids were resuspended in 800 µL 0.05% Trypsin-EDTA (Gibco) and incubated at 37°C for 5 minutes. The Trypsin was quenched with 200 µL RPMI 10% FBS 1% P/S +RI and organoids were pipetted up and down ten times to dissociate to single cells and passed through a 100µm cell strainer (Corning). Dissociated cells from mouse prostate organoids were washed with PBS and fixed in 1mL of 2% paraformaldehyde made from 16% paraformaldehyde (Electron Microscopy Sciences) in PBS for 15 minutes on ice. Cells were washed with PBS and permeabilized in 1 mL of permeabilization buffer (0.1% Saponin (Sigma-Aldrich), 5% FBS (Corning) in PBS) for 15 minutes at room temperature in the dark. Cells were resuspended in 100 mL of permeabilization buffer and stained with rabbit anti-cytokeratin 5-Alexa Fluor 647 (Abcam) and rabbit anti-cytokeratin 8-Alexa Fluor 488 (Abcam) for 20 minutes at room temperature in the dark. Cells were washed with permeabilization buffer and resuspended in PBS for analysis on a BD FACS Canto (BD Biosciences). For apoptosis analysis, Cell culture media and wash media were collected and pooled with quenched trypsin-containing media containing cells and apoptosis analysis was performed using an apoptosis detection kit (BioLegend, 640922) according to manufacturer instructions. Flow cytometry was performed to quantify the percentage of annexin V-, 7-AAD- cells. For EdU labeling assay, cells were seeded at 30 percent confluence and cultured in 6-well dishes for 72 hours prior to cell cycle analysis. Media changes were performed 48 hours after plating. After 72 hours of culture, cell cycle analysis was performed using a 5-ethynyl-2'-deoxyuridine-based (EdU) kit (Thermo Fisher Scientific, C10635) according to the specified protocol. EdU labeling was performed for 2 hours. For experiments that contained small molecule inhibitors, fresh inhibitor(s) were adding during each media change. Flow cytometry analysis identified the percentage of EdU-positive.

Instrument

Analysis was performed using a BD LSRFortessa and BD FACS Canto. Sorting was performed on a BD FACS Aria II (BD Biosciences).

Software

FACSDiva software (8.0.2)

Cell population abundance

Post-sort purity was assessed routinely by re-running sorted samples and confirming greater than 95% purity of the specific epithelial population.

Gating strategy

Forward and side scatter were used to identify cells of interest and exclude debris. Doublets were excluded by analyzing the remaining cells by forward scatter height and forward scatter width. Gates and compensation were set by first running negative or single color controls.

☒ Tick this box to confirm that a figure exemplifying the gating strategy is provided in the Supplementary Information.
