# Supplementary material for: Prostate lineage-specific metabolism governs luminal differentiation and response to antiandrogen treatment
Source: Nat Cell Biol. 2023 Dec 4;25(12):1821–32. doi: 10.1038/s41556-023-01274-x (PMC10709144; doi:10.1038/s41556-023-01274-x)

Figure 2d

KRT8

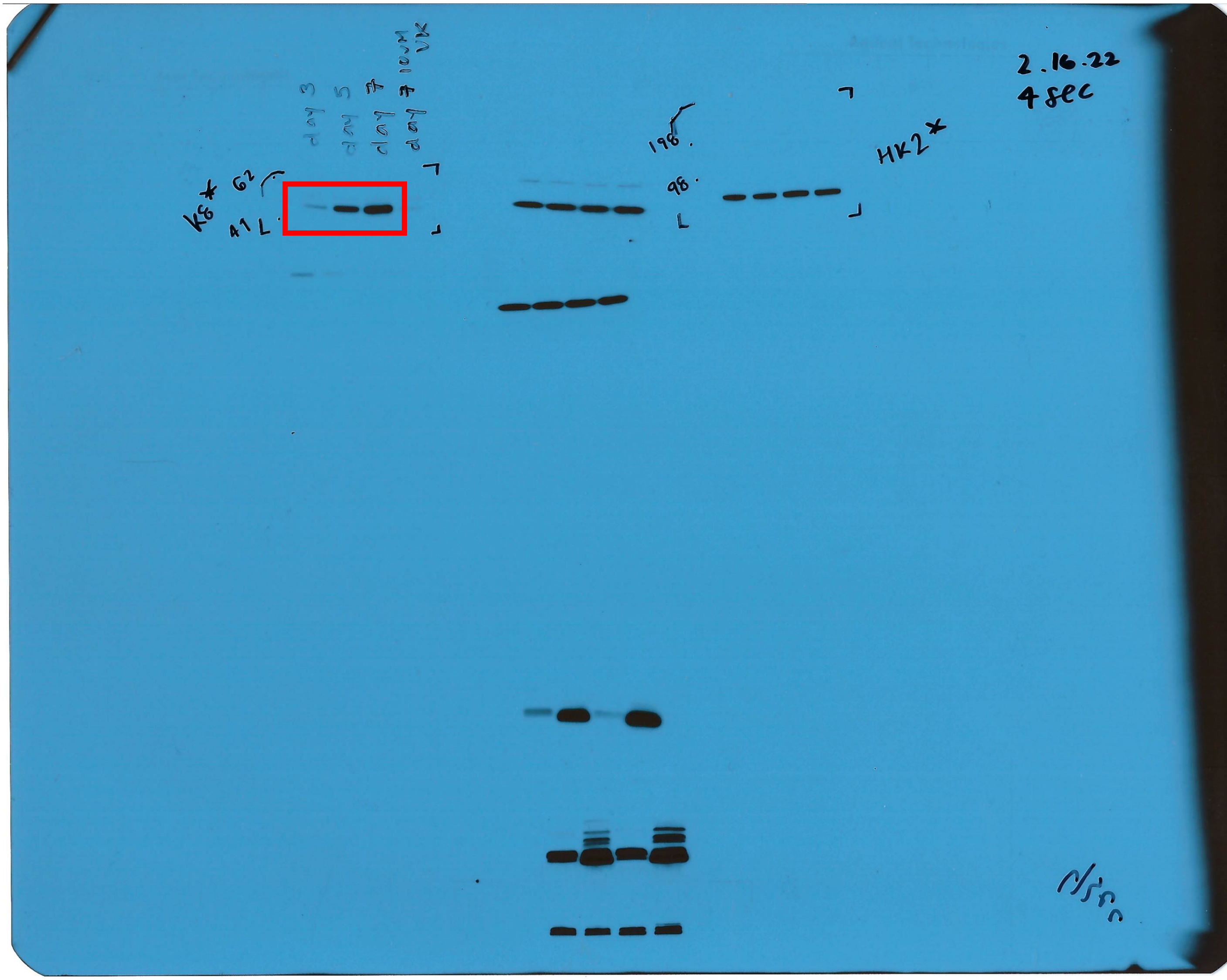

Figure 2d

p63

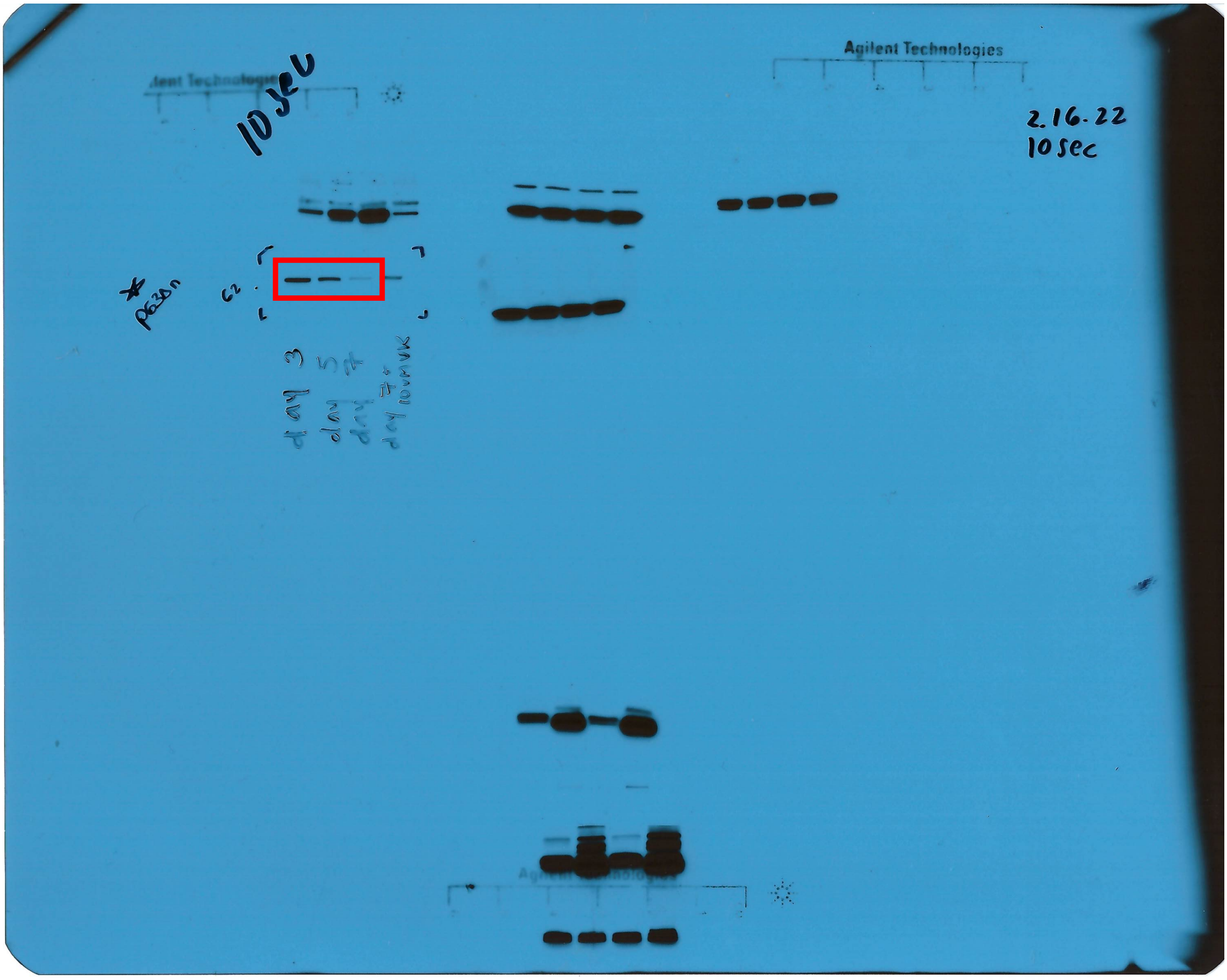

Figure 2d

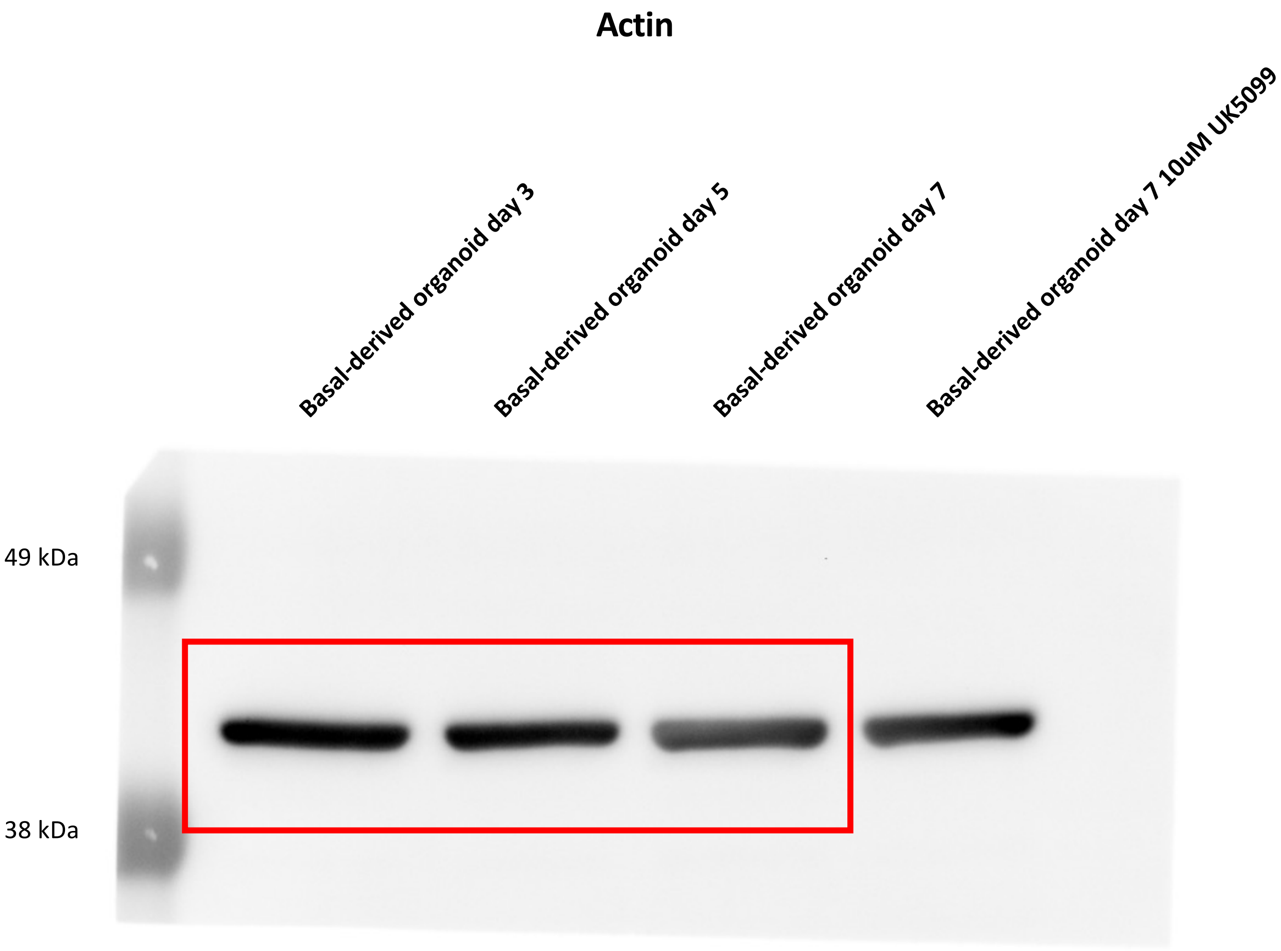
Actin
Basal-derived organoid day 3
Basal-derived organoid day 5
Basal-derived organoid day 7
Basal-derived organoid day 7 10uM UK5099
49 kDa
38 kDa

Figure 3d

AR

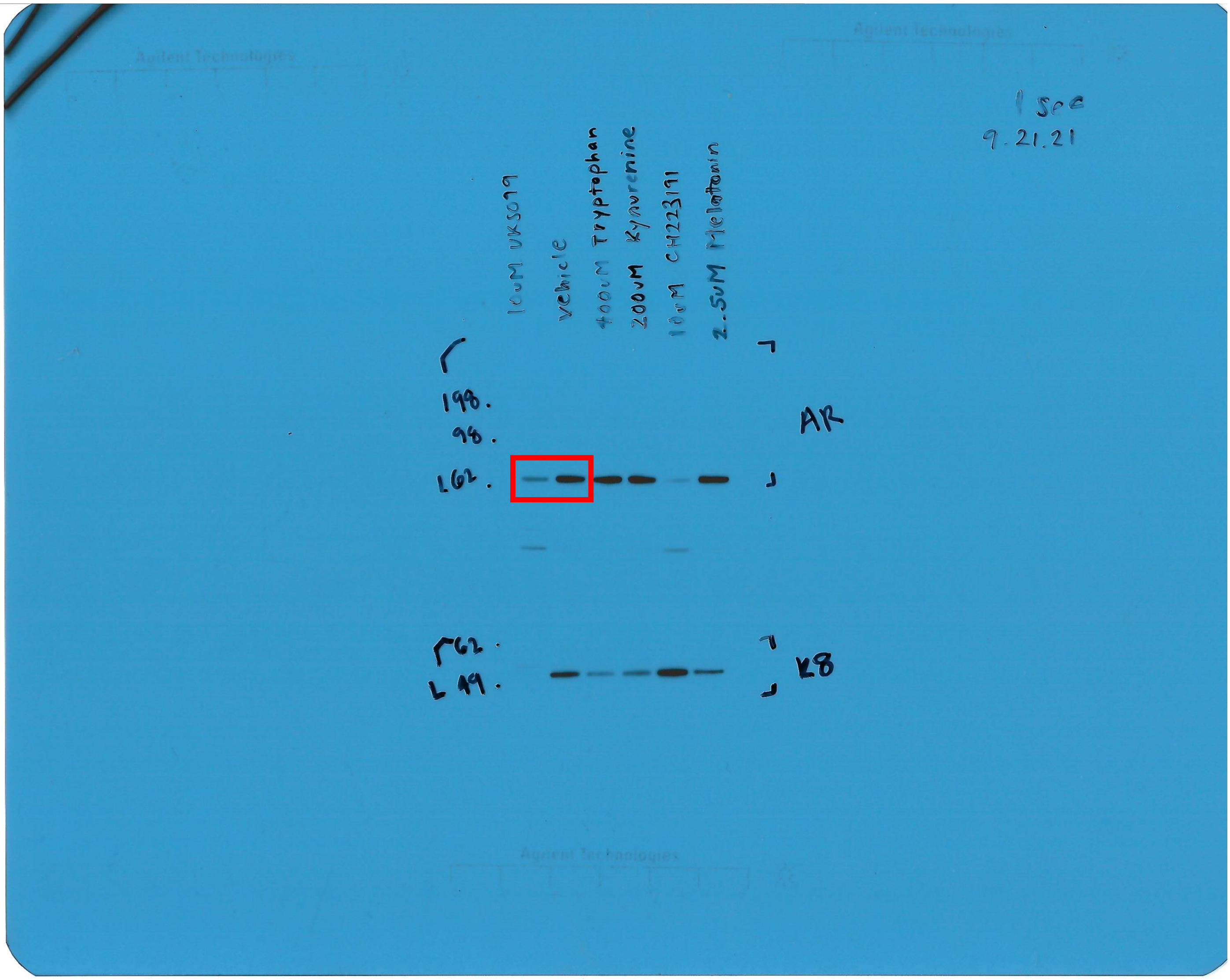

1 sec
9.21.21
10uM UK5099
vehicle
400uM Tryptophan
200uM Kynurenine
10uM CH223191
2.5uM Melatonin
198.
98.
62.
AR
62.
49.
K8

Figure 3d

KRT8

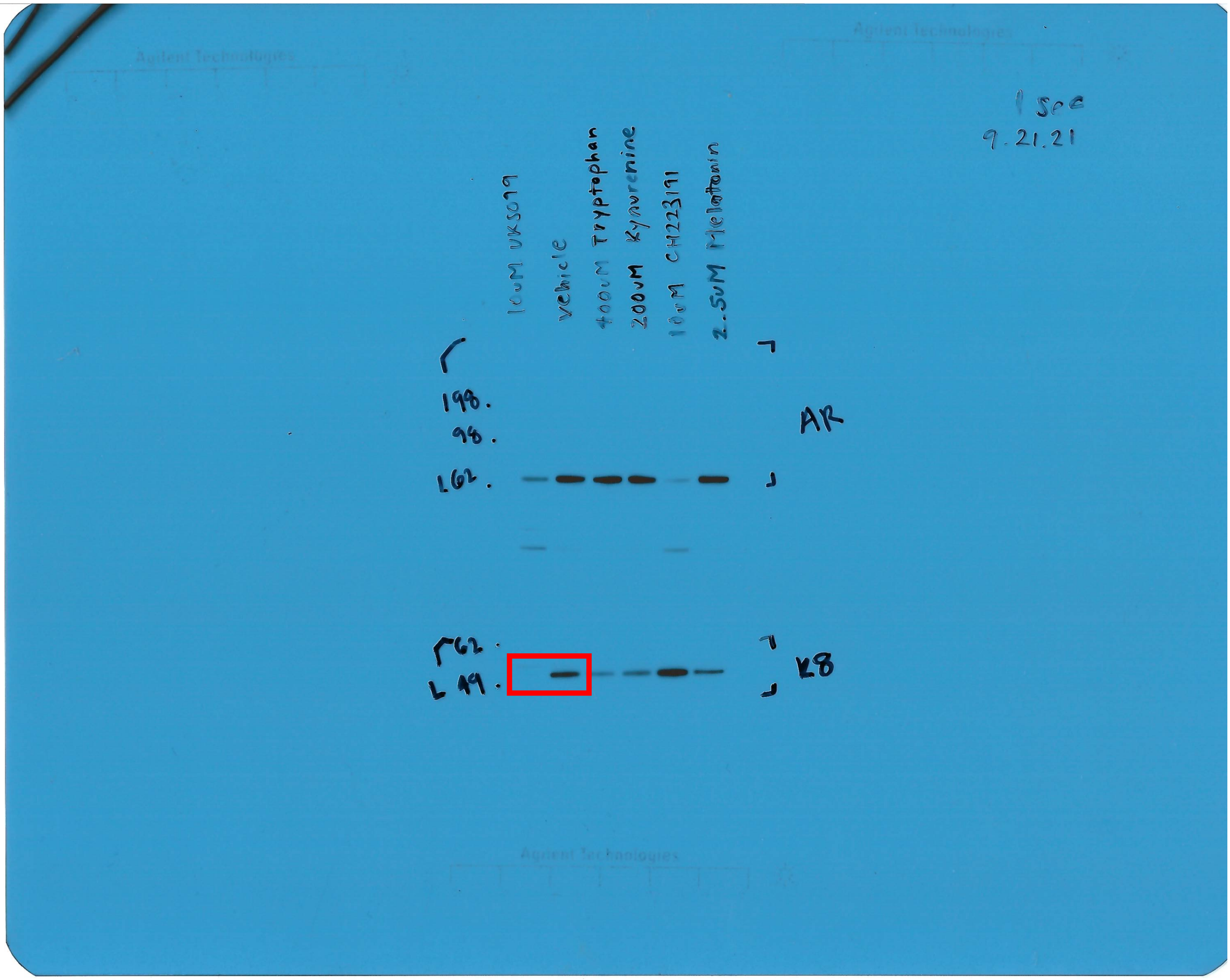

Figure 3d

p63

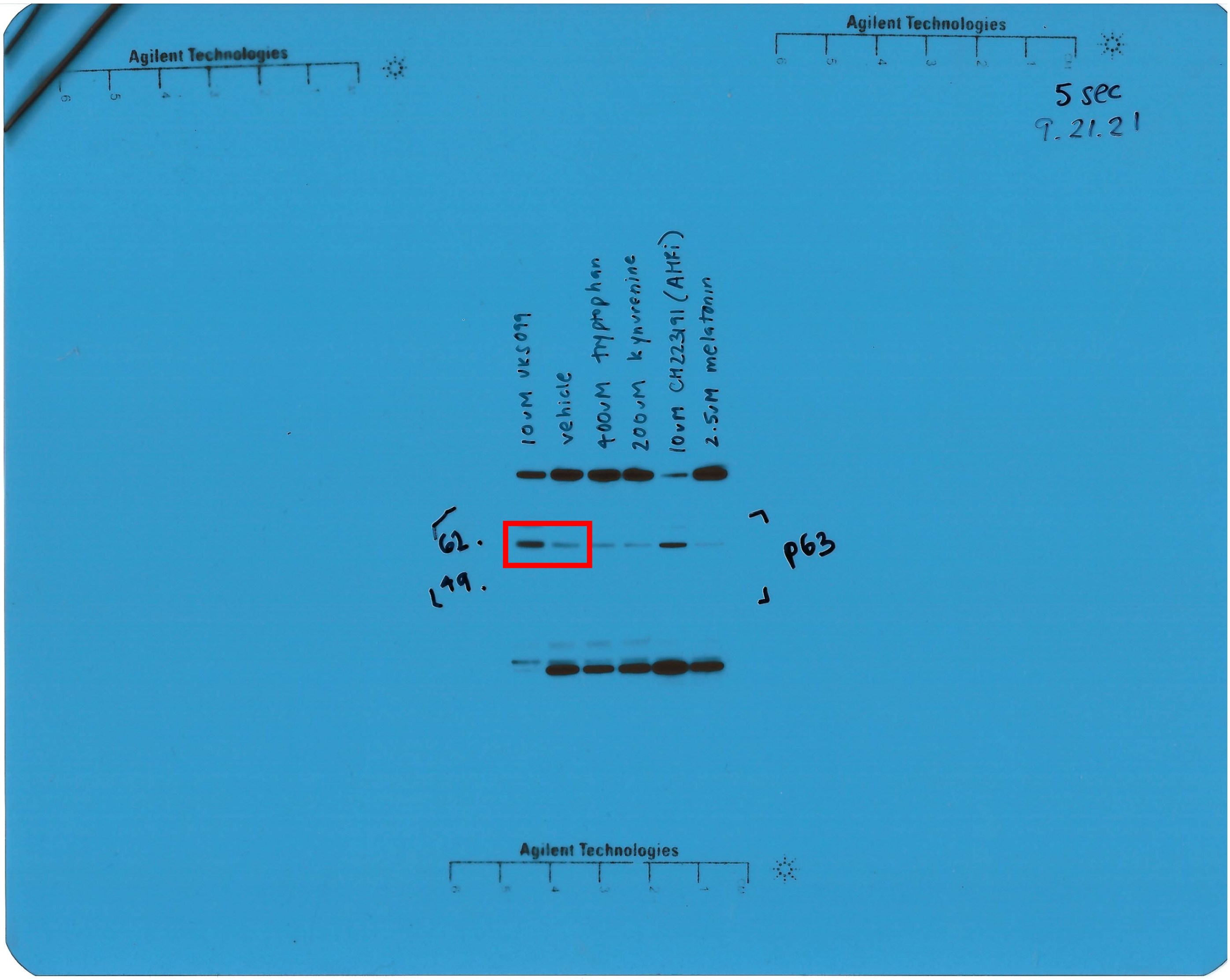

Figure 3d

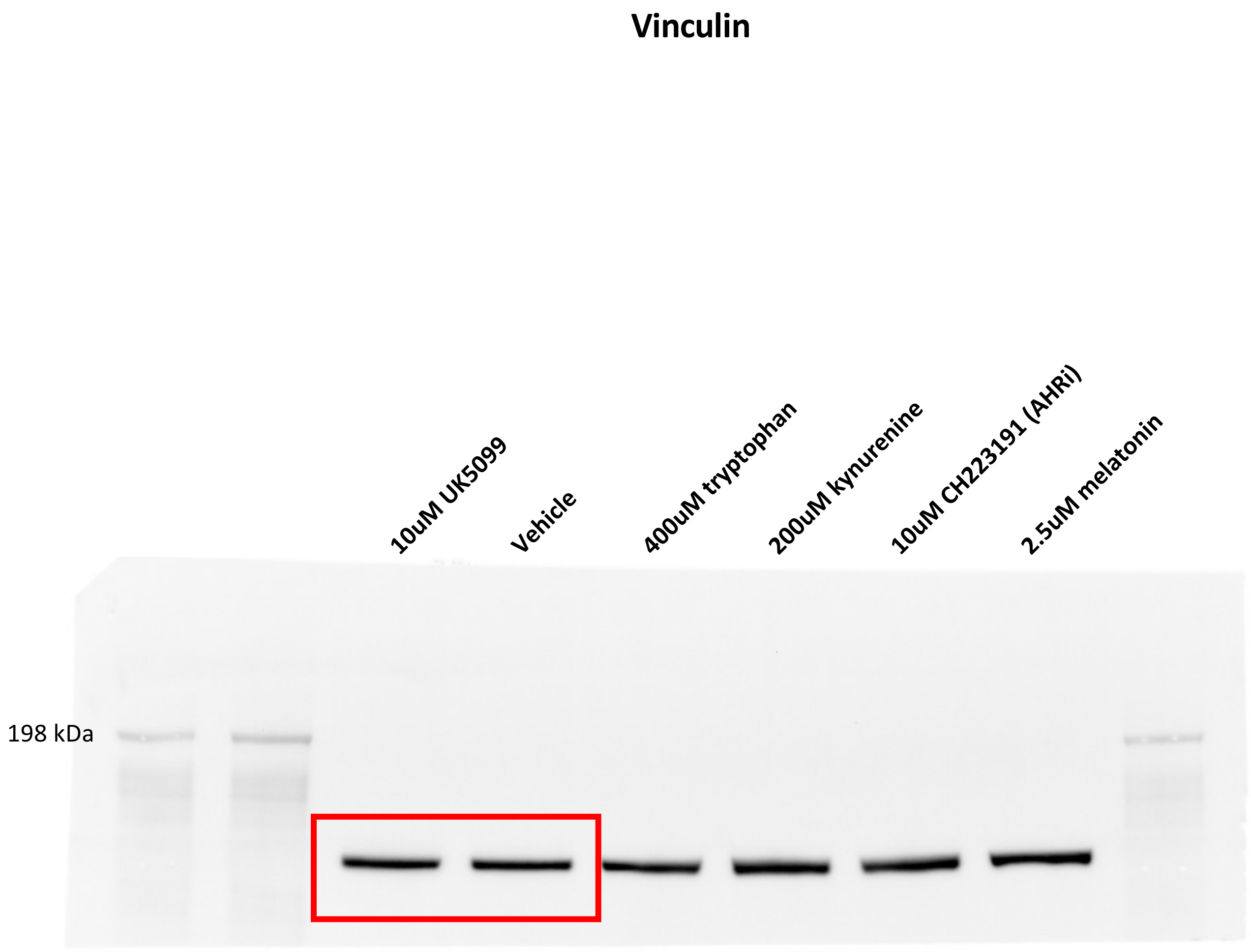
Vinculin
10uM UK5099
Vehicle
400uM tryptophan
200uM kynurenine
10uM CH223191 (AHRi)
2.5uM melatonin
198 kDa

Figure 3i

KRT8

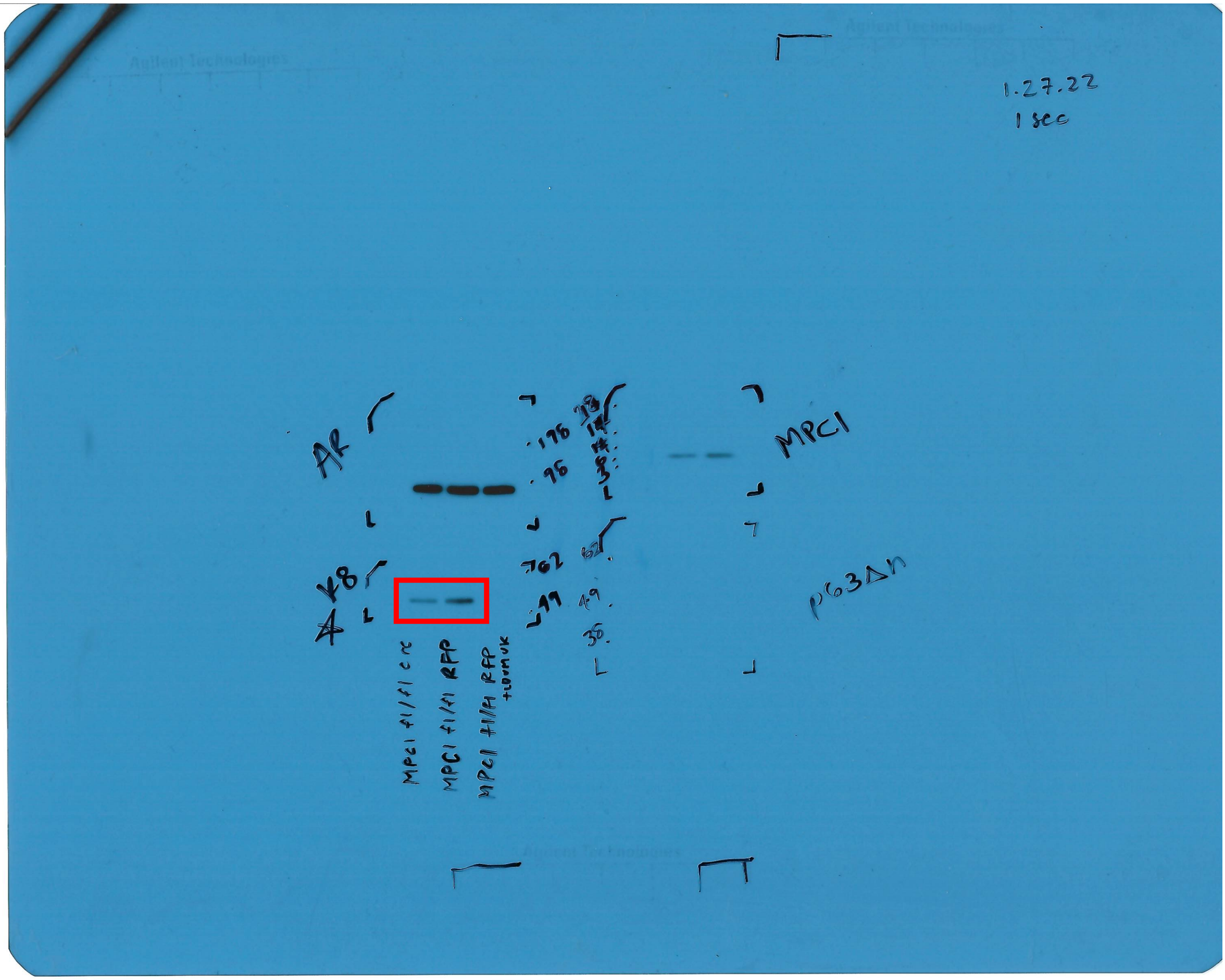

Figure 3i

p63

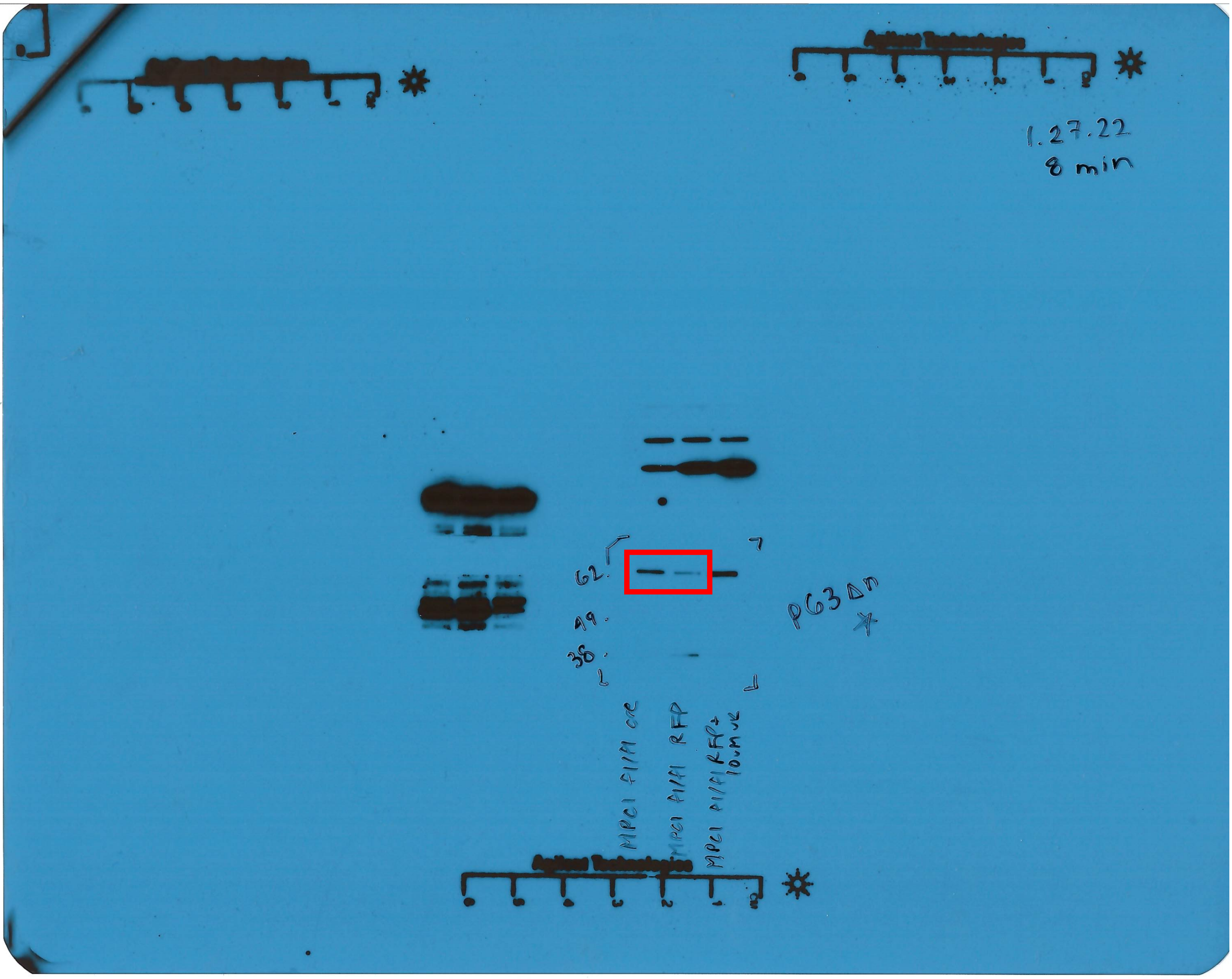

Figure 3i

MPC1

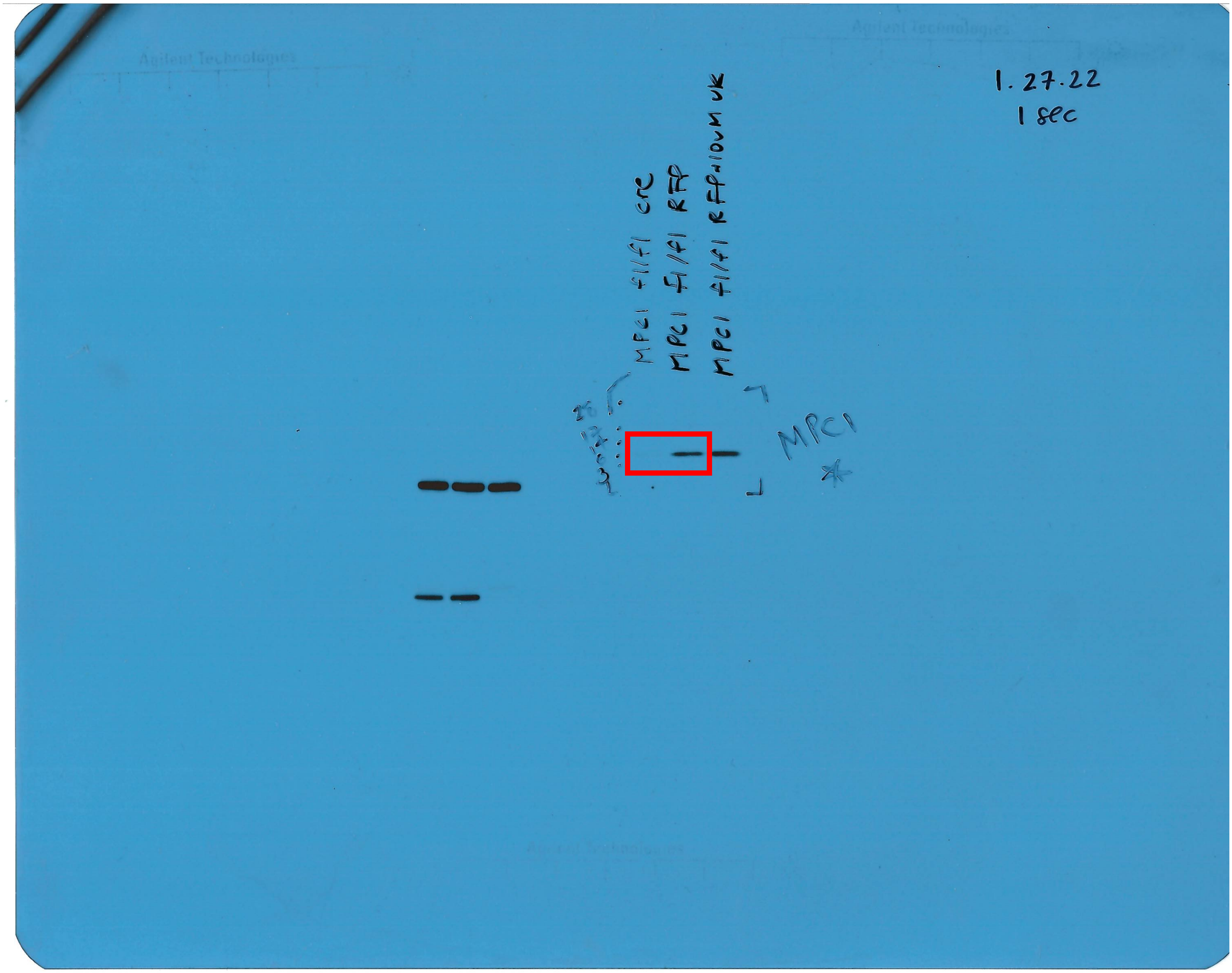

Figure 3i

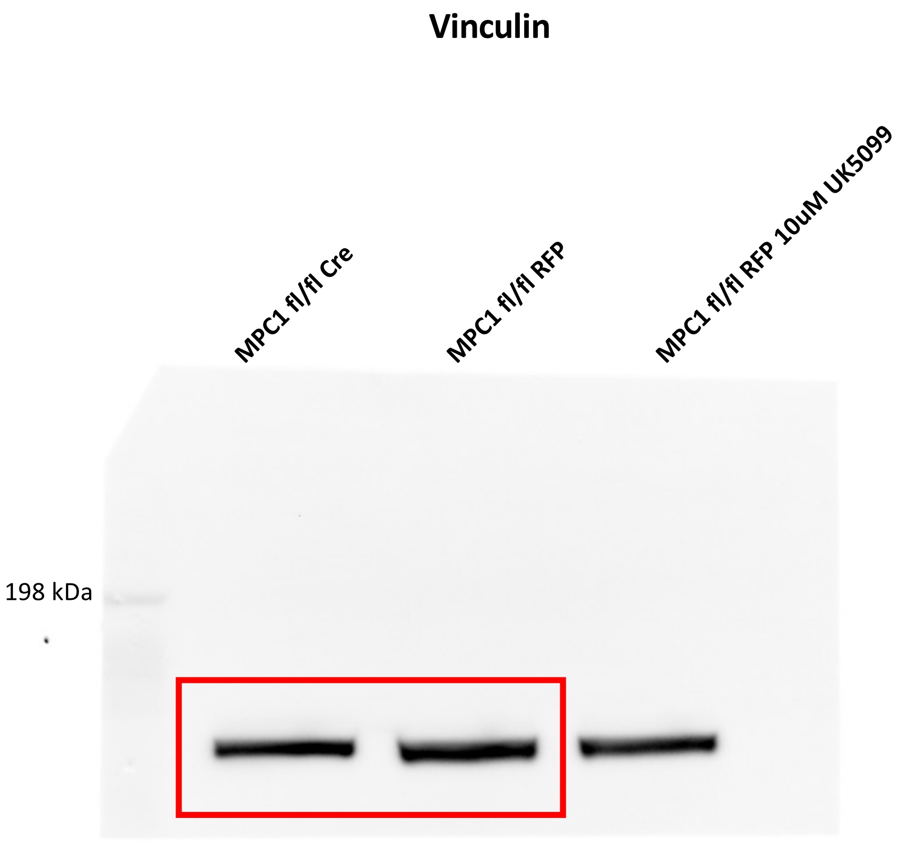

Vinculin
MPC1 fl/fl Cre
MPC1 fl/fl RFP
MPC1 fl/fl RFP 10uM UK5099
198 kDa

Figure 4a

KRT8

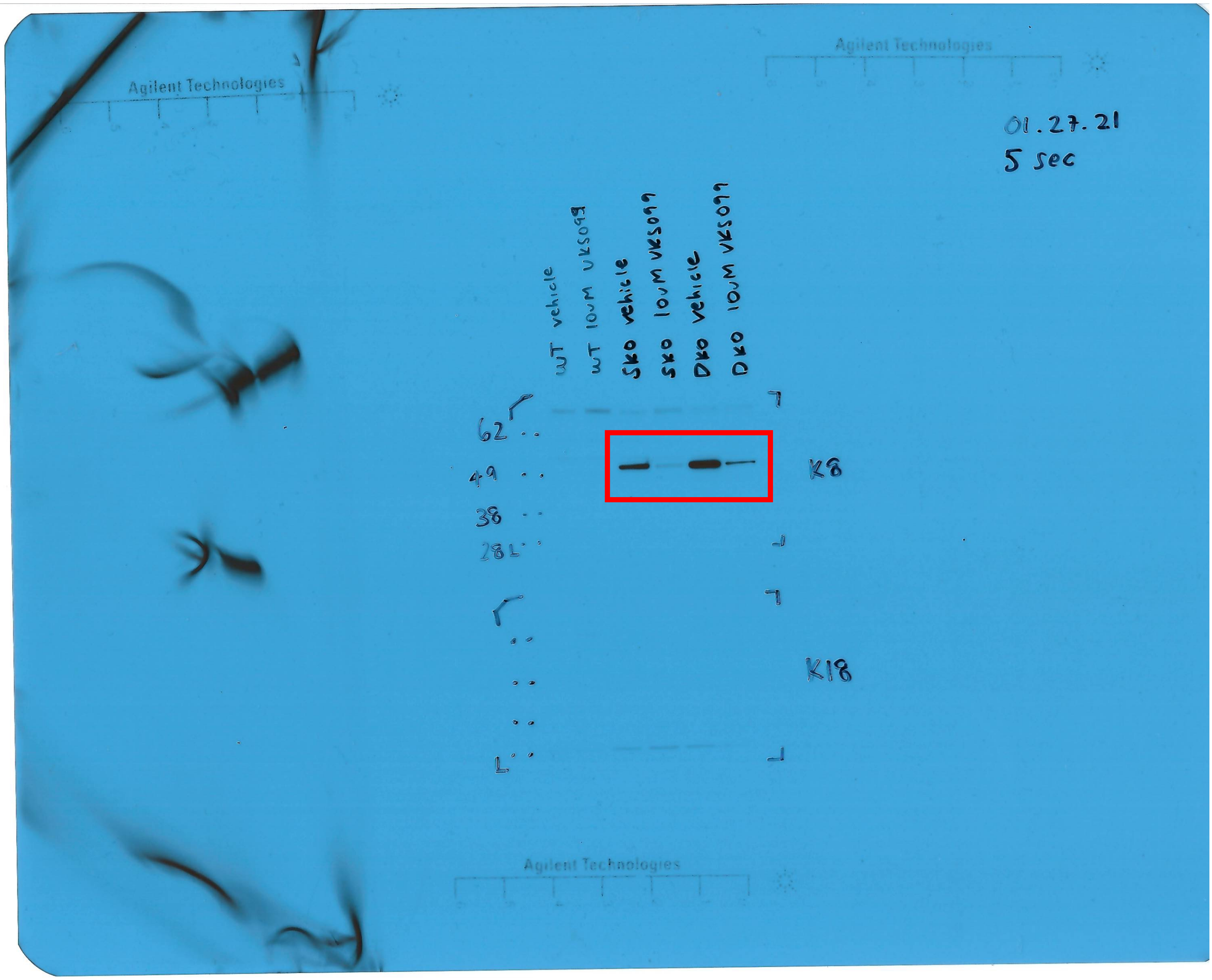

Figure 4a

KRT18

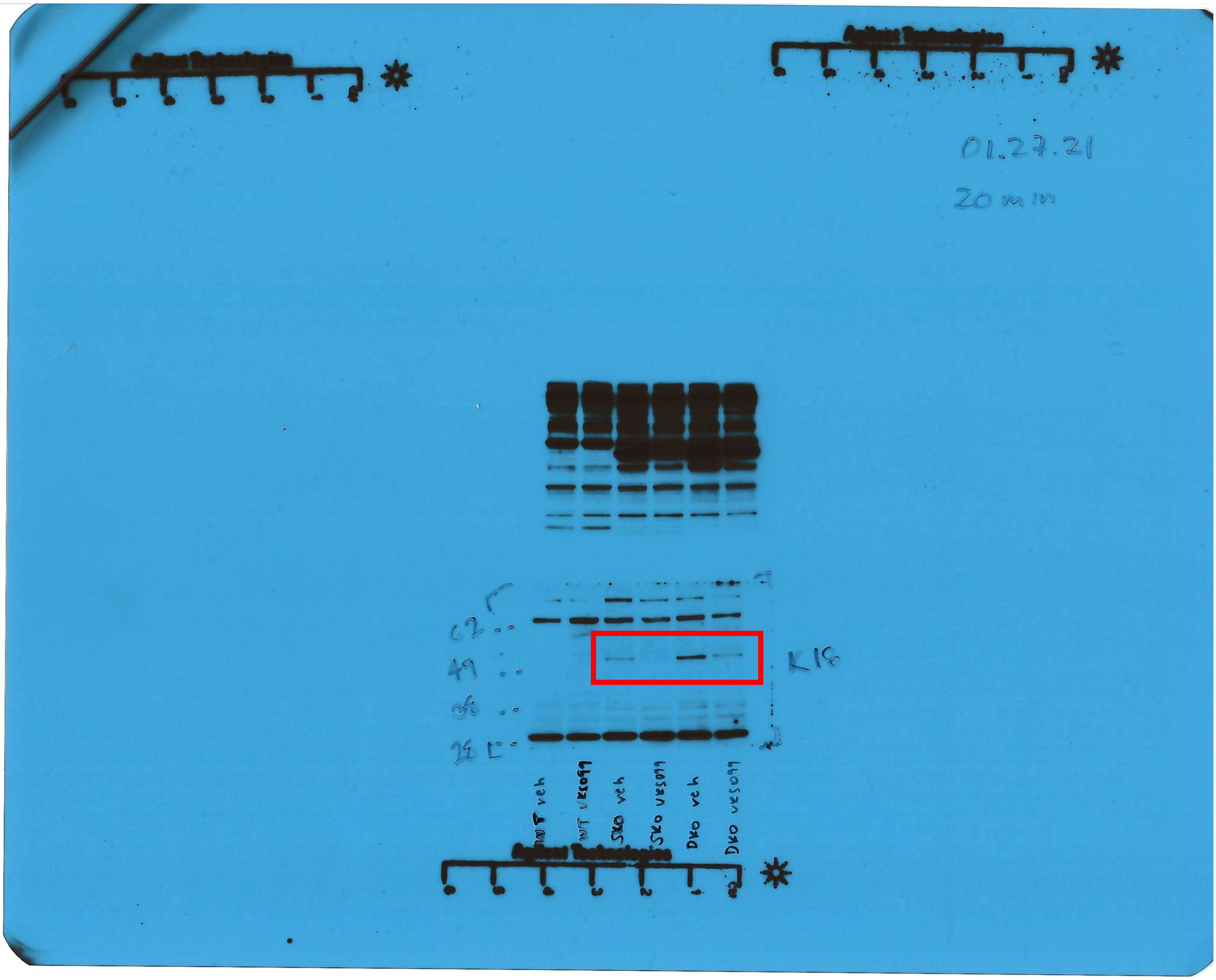

Figure 4a

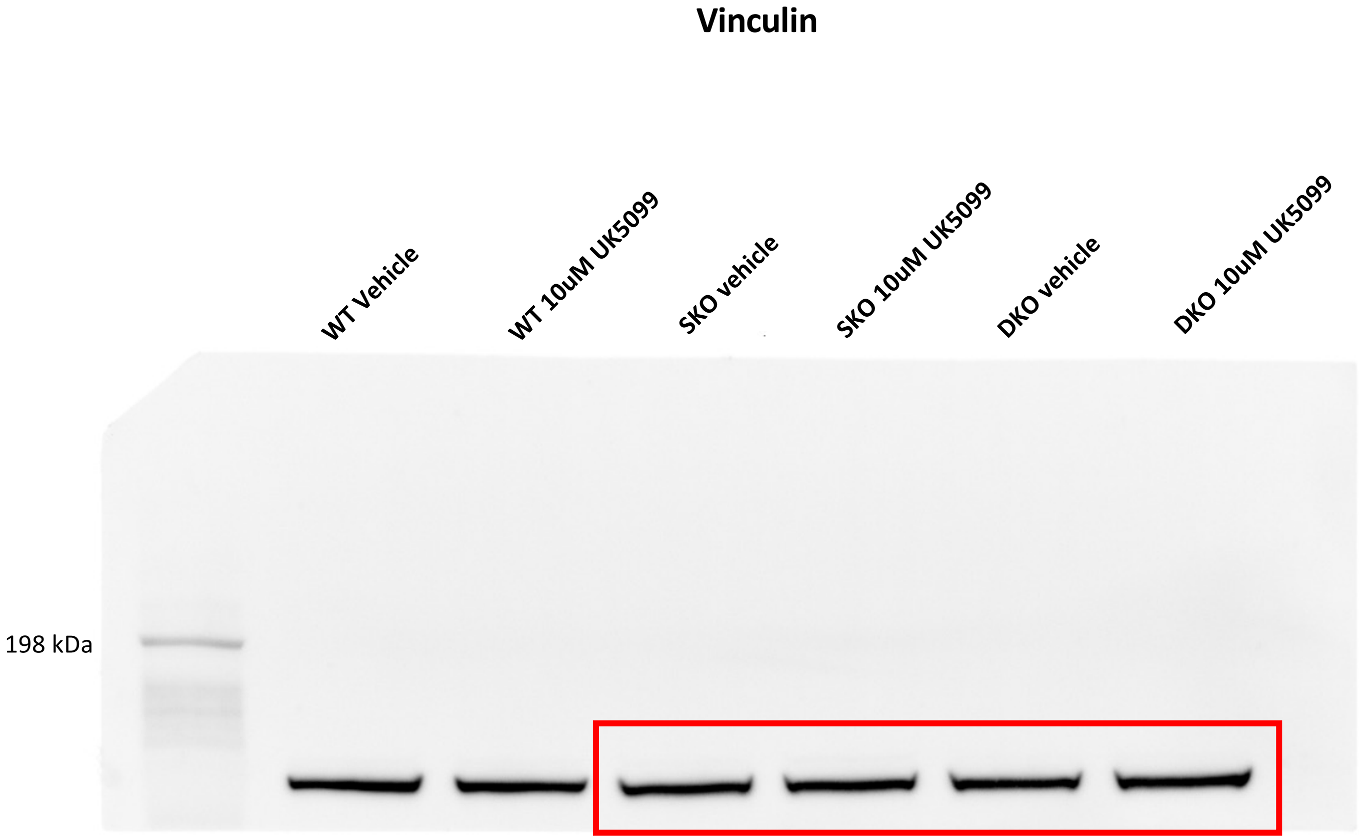

Vinculin
WT Vehicle
WT 10uM UK5099
SKO vehicle
SKO 10uM UK5099
DKO vehicle
DKO 10uM UK5099
198 kDa

Figure 5b

KRT8

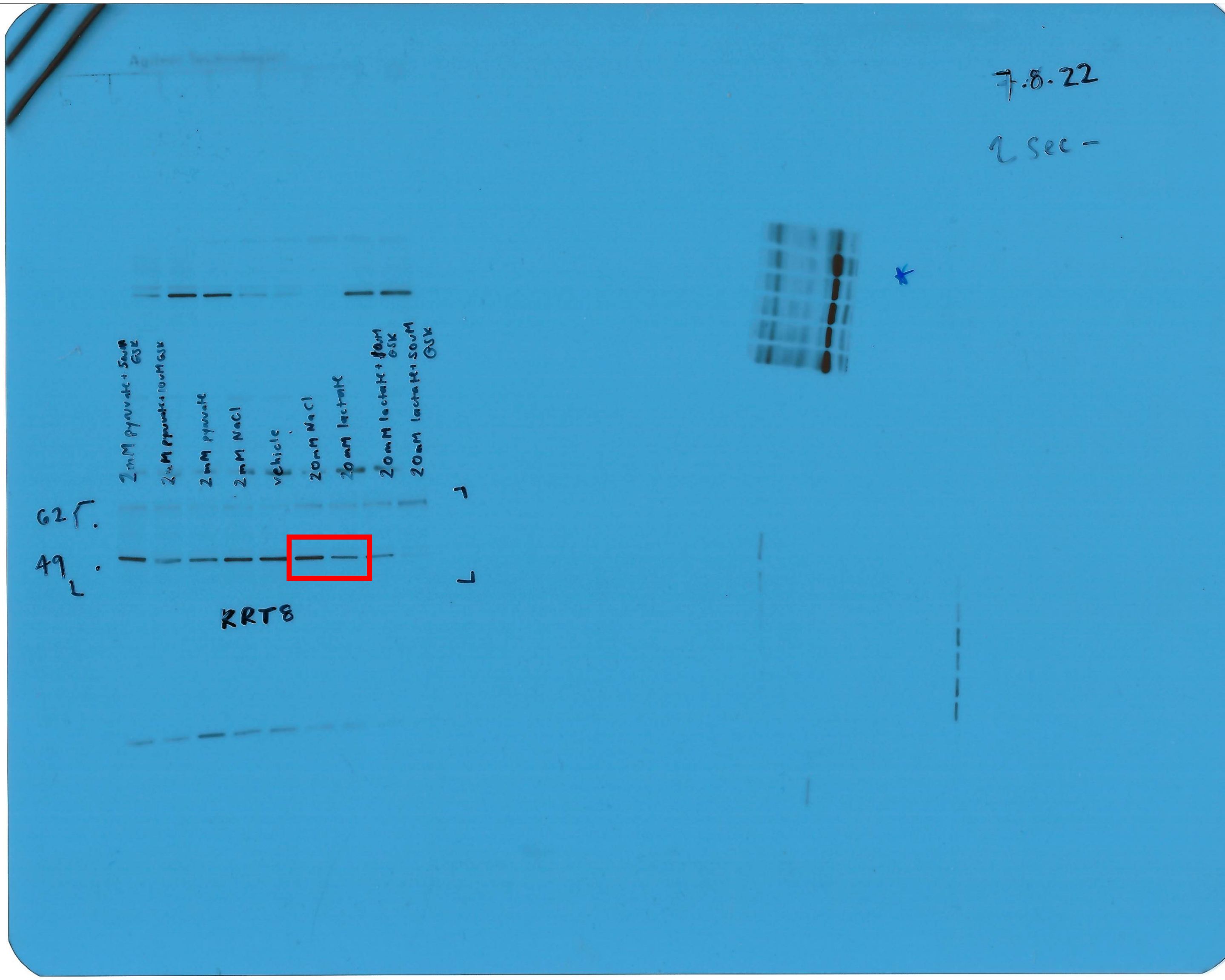

Figure 5b

p63

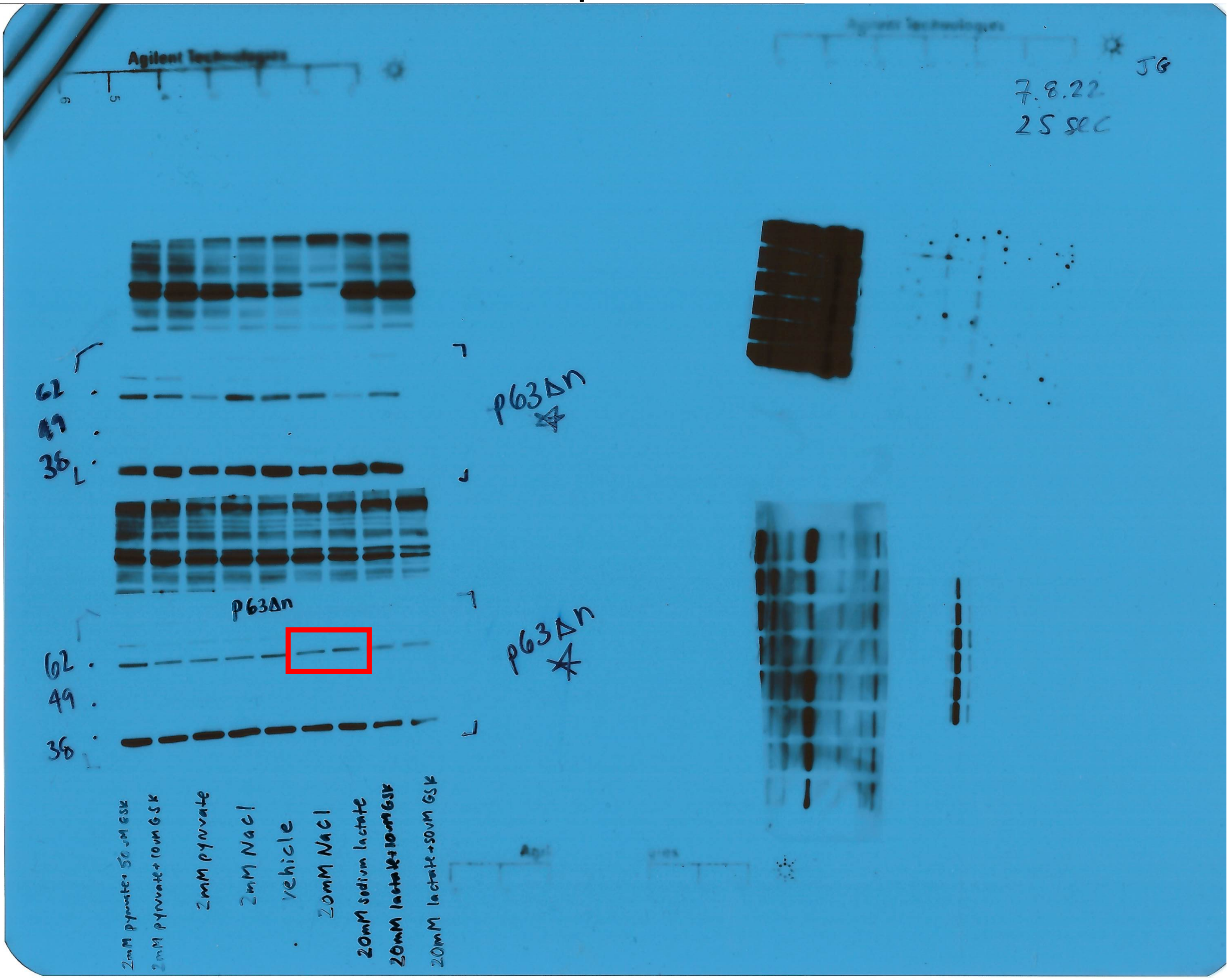

Figure 5b

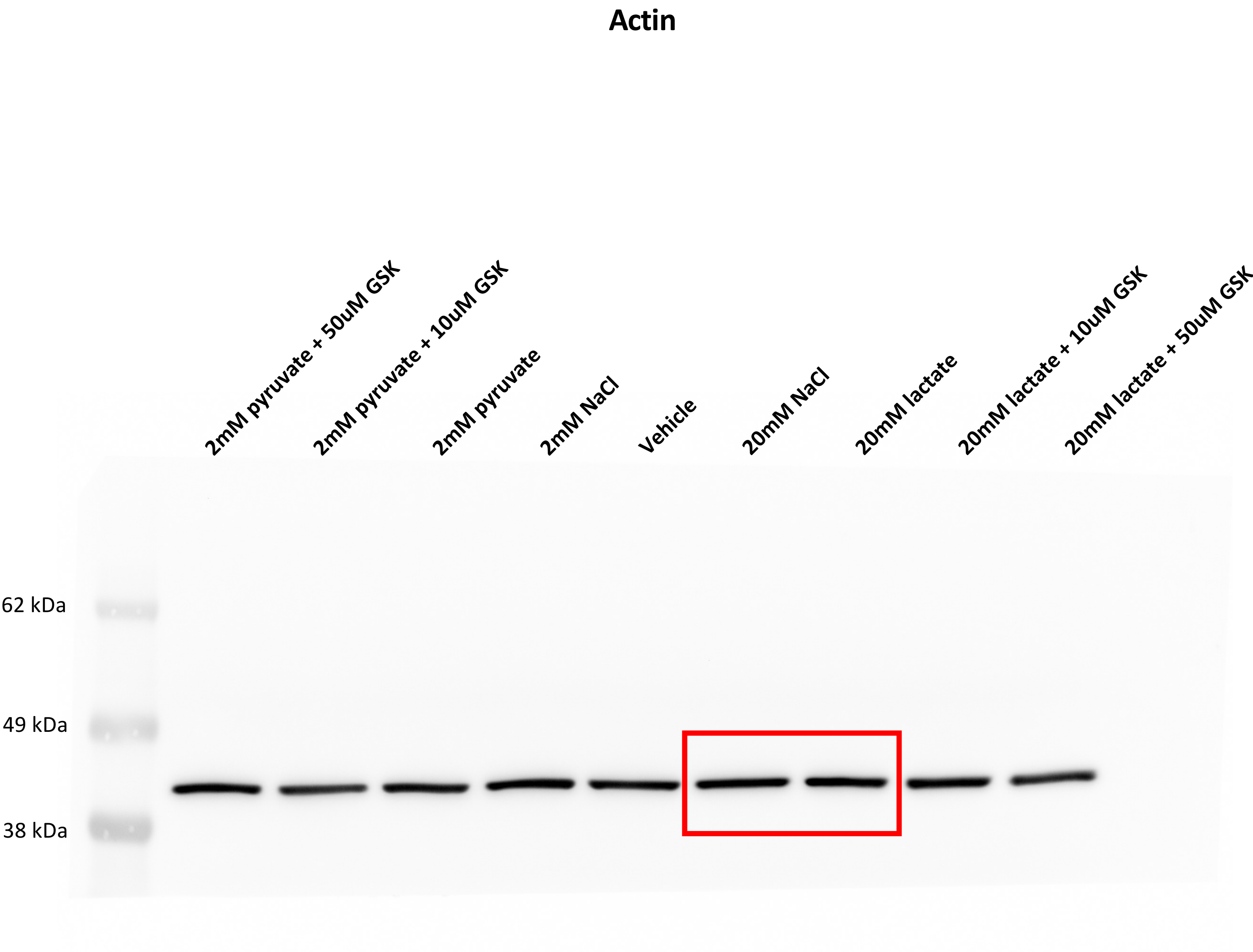
Actin
2mM pyruvate + 50uM GSK
2mM pyruvate + 10uM GSK
2mM pyruvate
2mM NaCl
Vehicle
20mM NaCl
20mM lactate
20mM lactate + 10uM GSK
20mM lactate + 50uM GSK
62 kDa
49 kDa
38 kDa

Figure 5c

KRT8

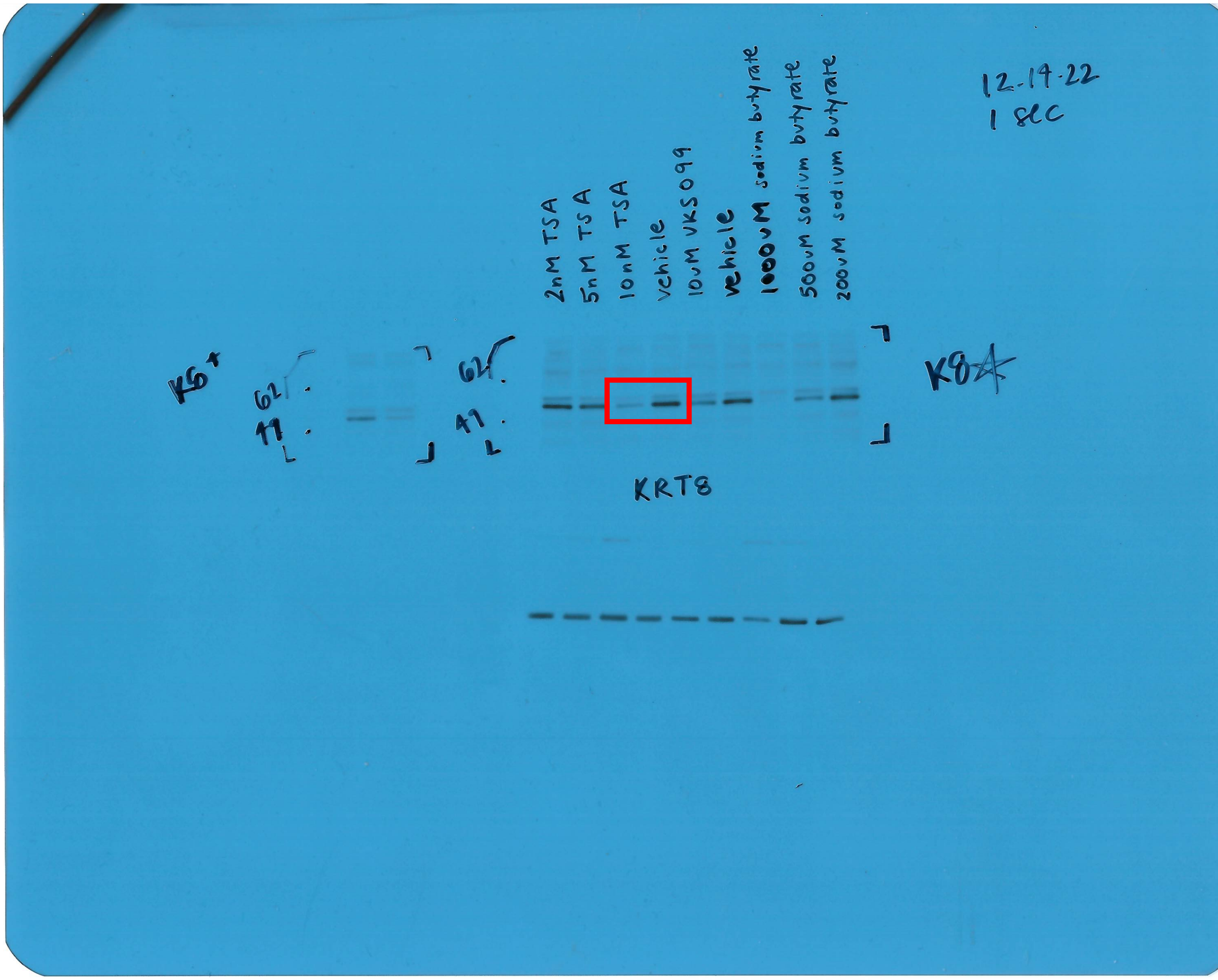

Figure 5c

p63

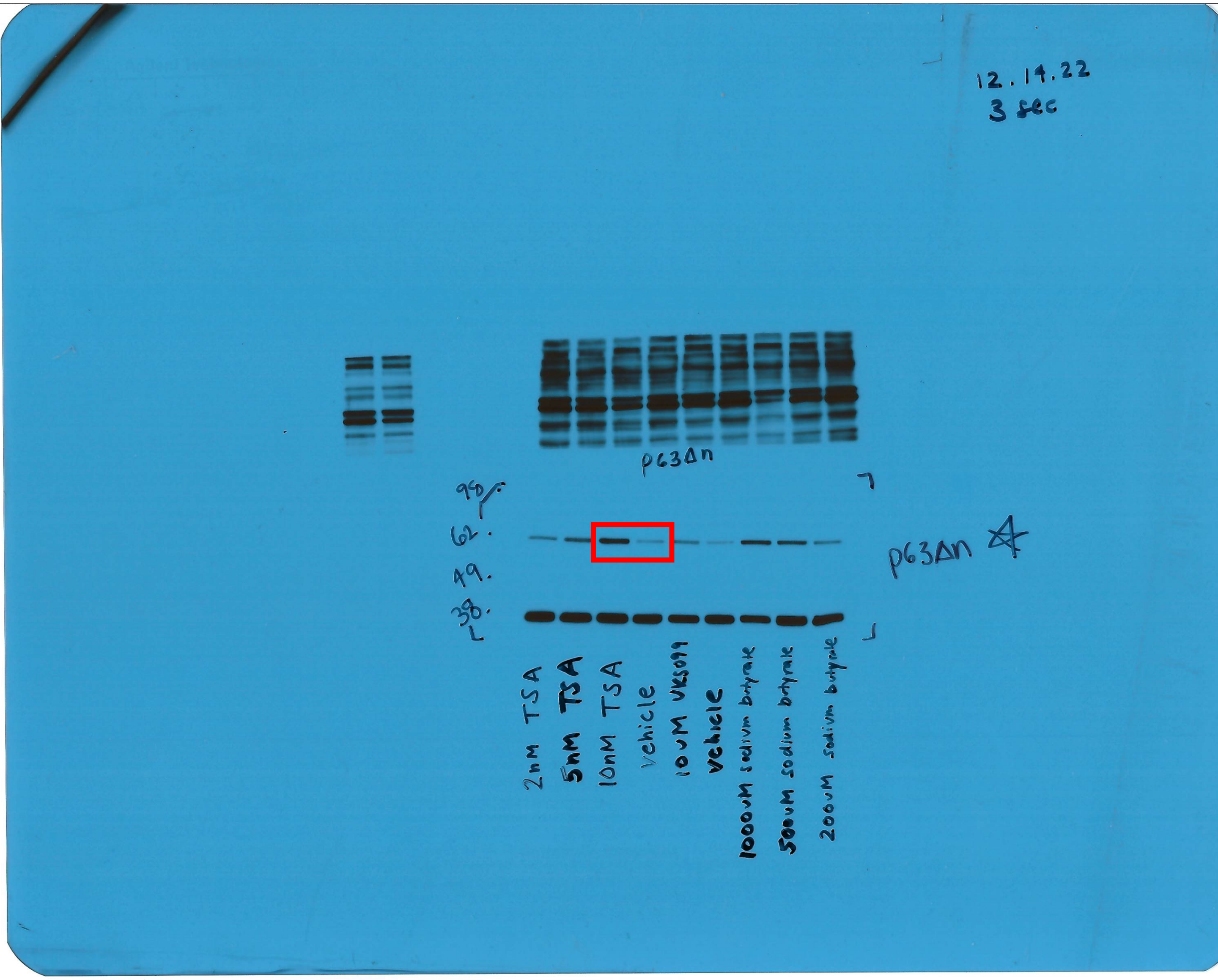

Figure 5c

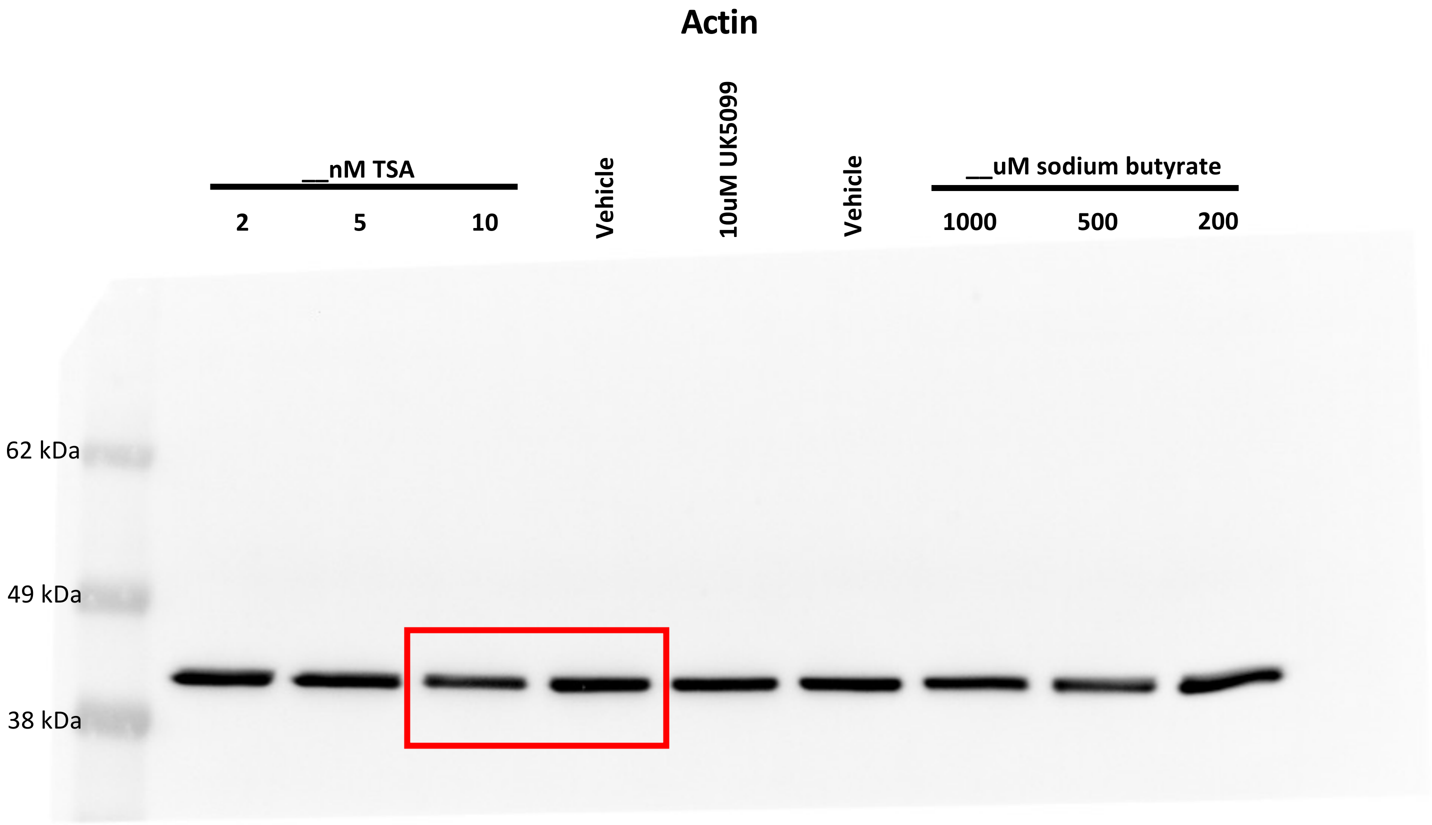

Actin
__nM TSA
2
5
10
Vehicle
10uM UK5099
Vehicle
__uM sodium butyrate
1000
500
200
62 kDa
49 kDa
38 kDa

Figure 5d

KRT8

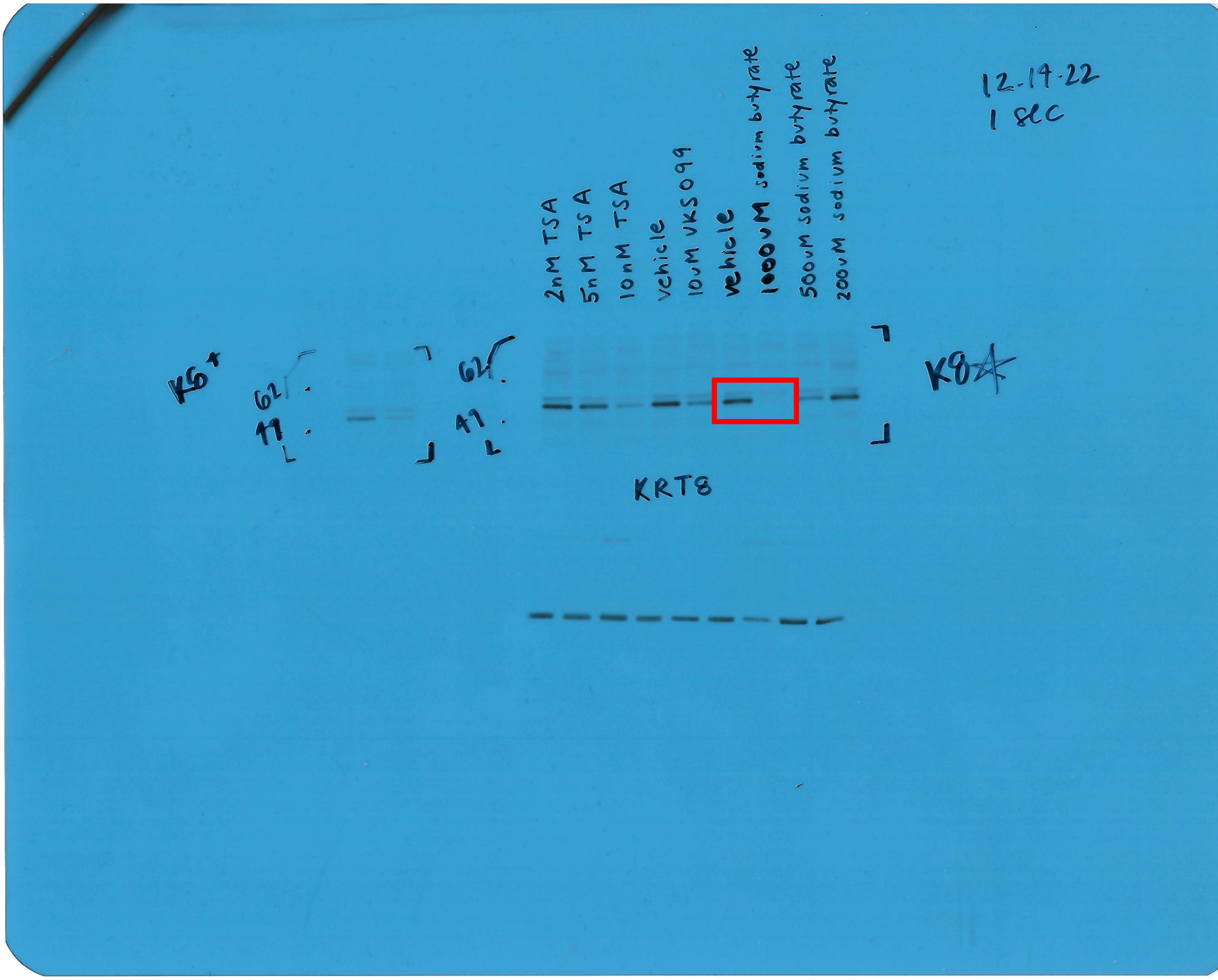

Figure 5d

p63

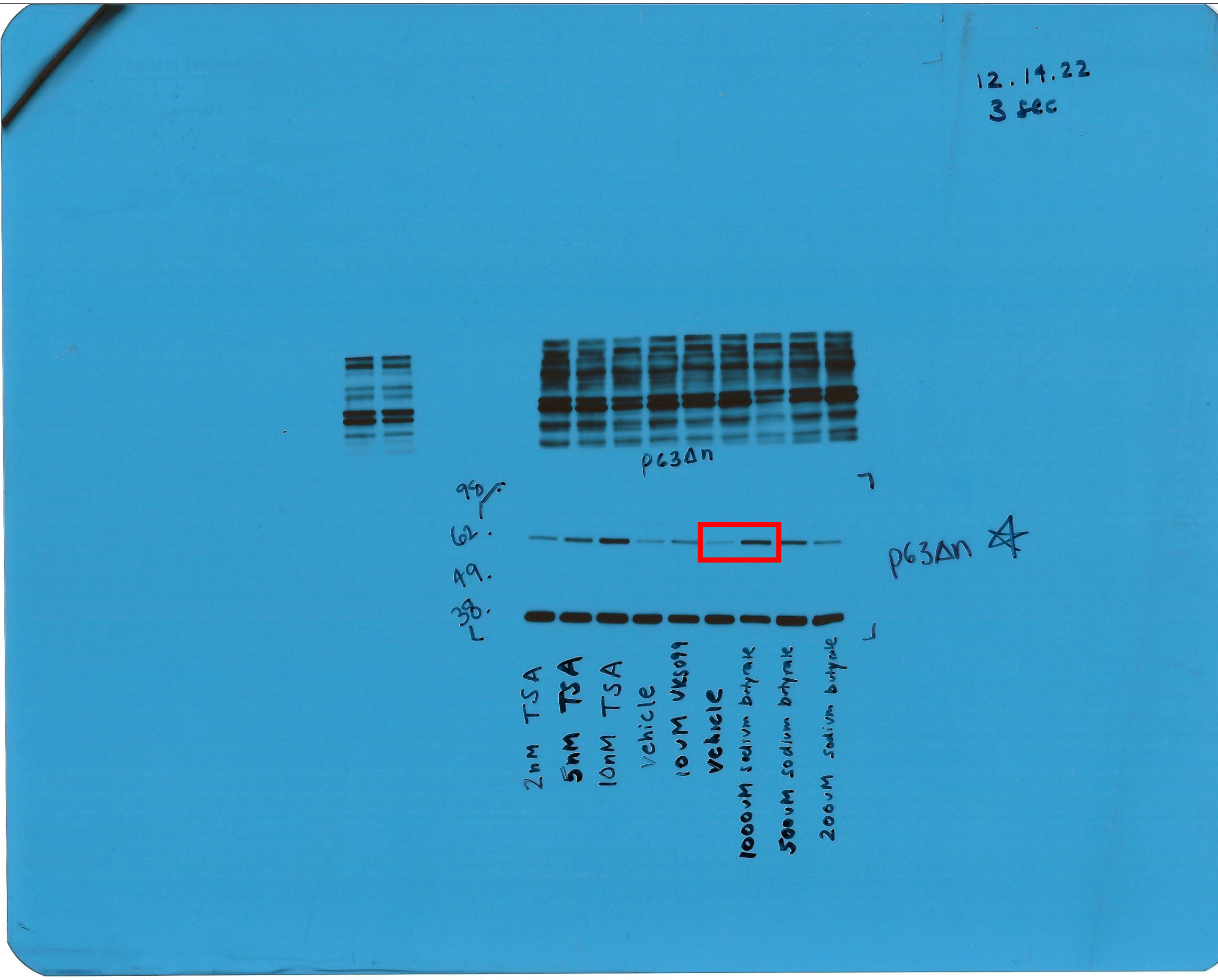

Figure 5d

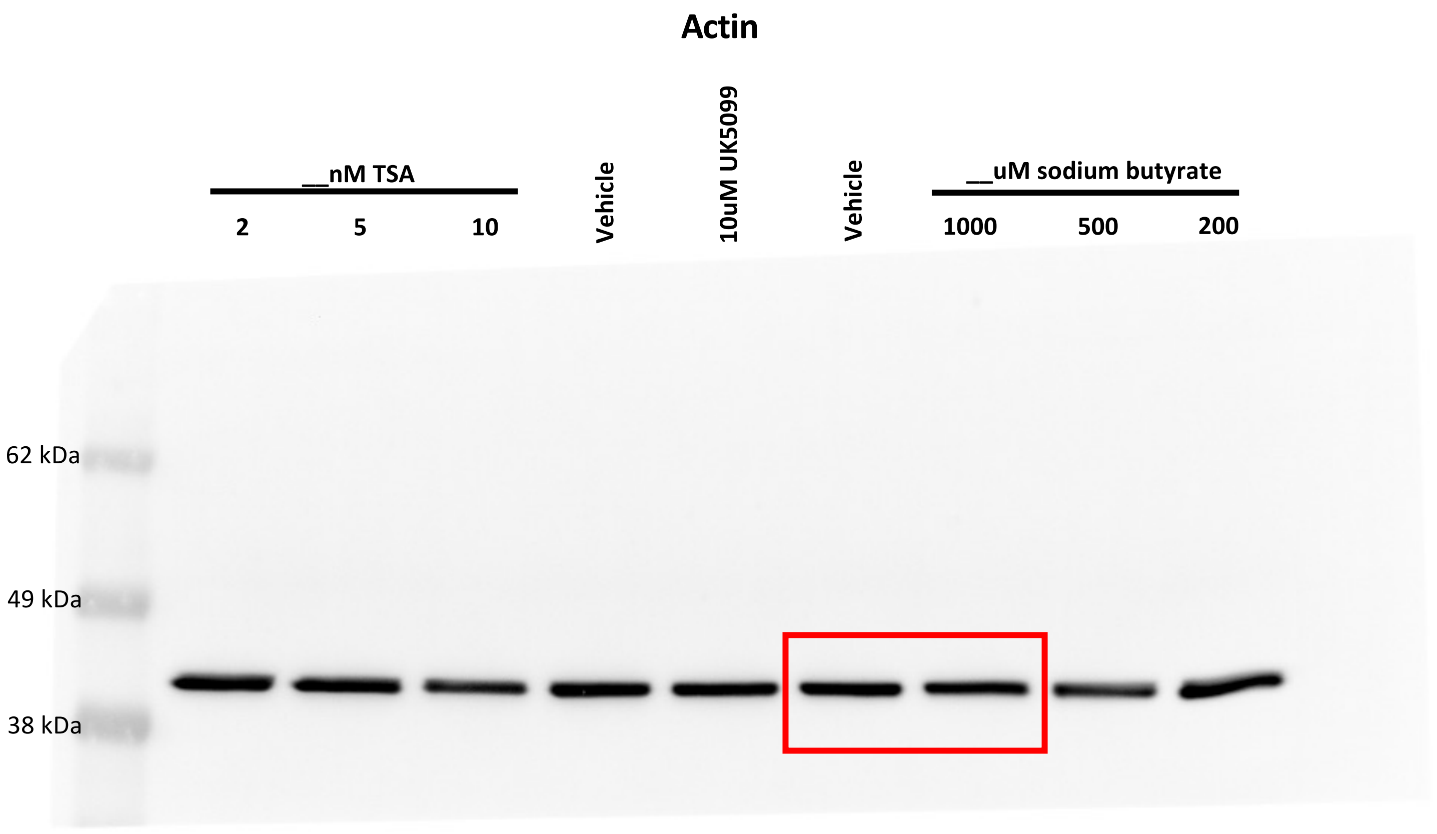

Actin
__nM TSA
2
5
10
Vehicle
10uM UK5099
Vehicle
__uM sodium butyrate
1000
500
200
62 kDa
49 kDa
38 kDa

Extended Data Figure 1f

KRT5

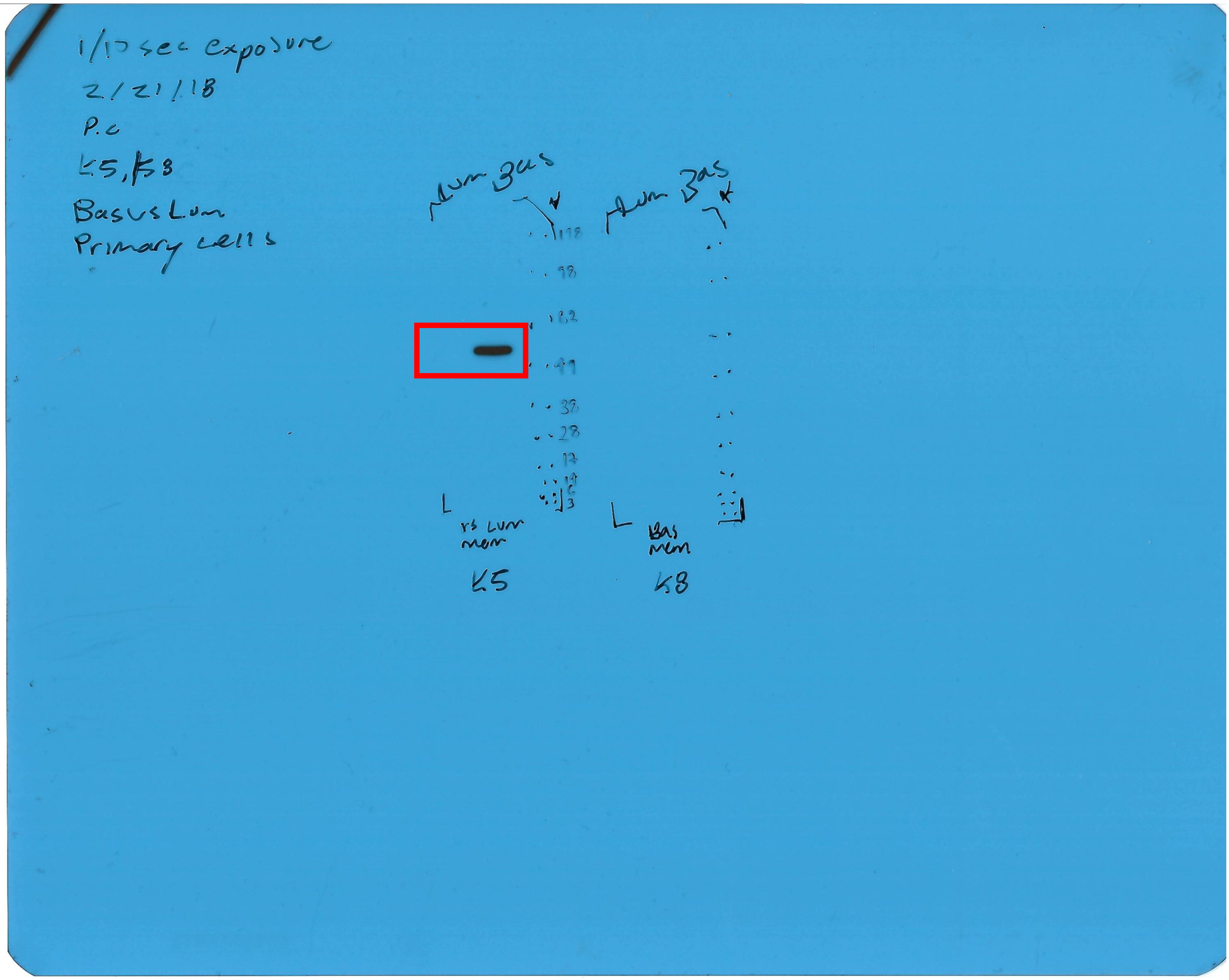

Extended Data Figure 1f

PBSN

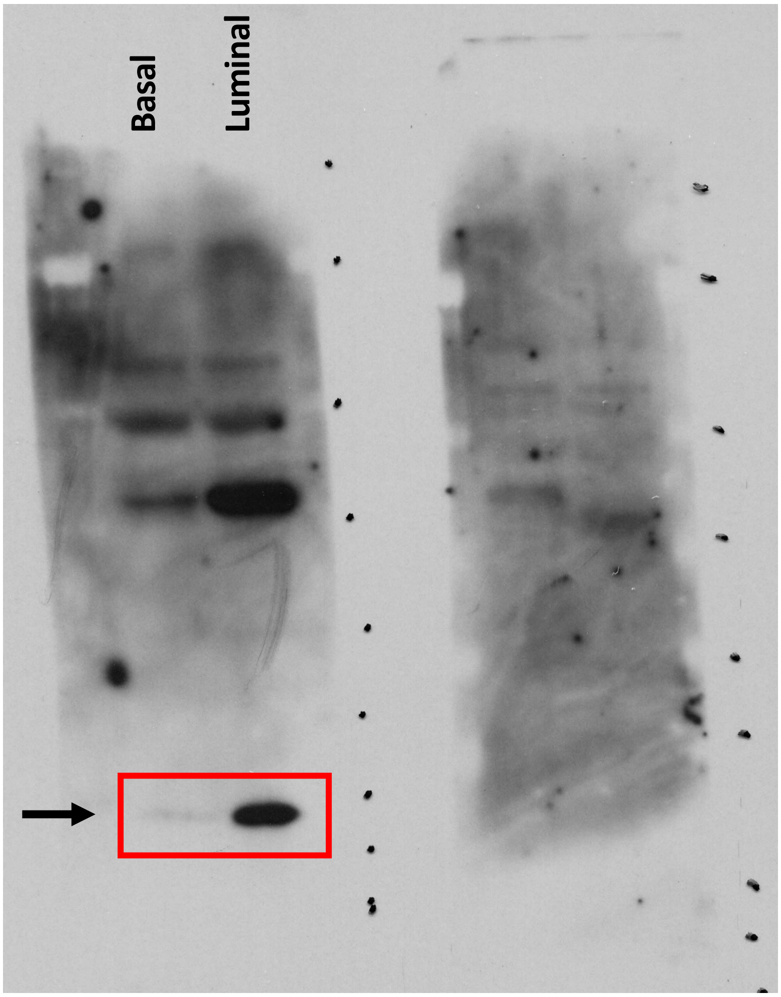

PBSN (~22kDa)

Slot-blot – SYPRO Ruby

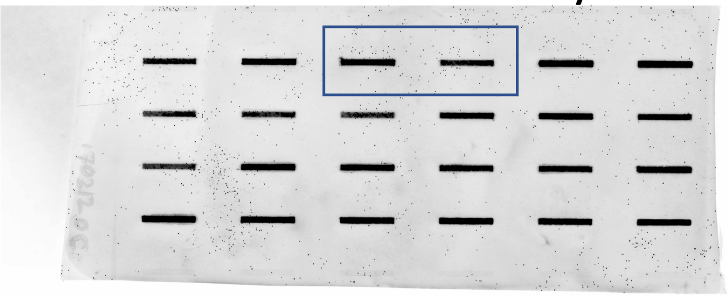

Extended Data Figure 1f

GLUT1

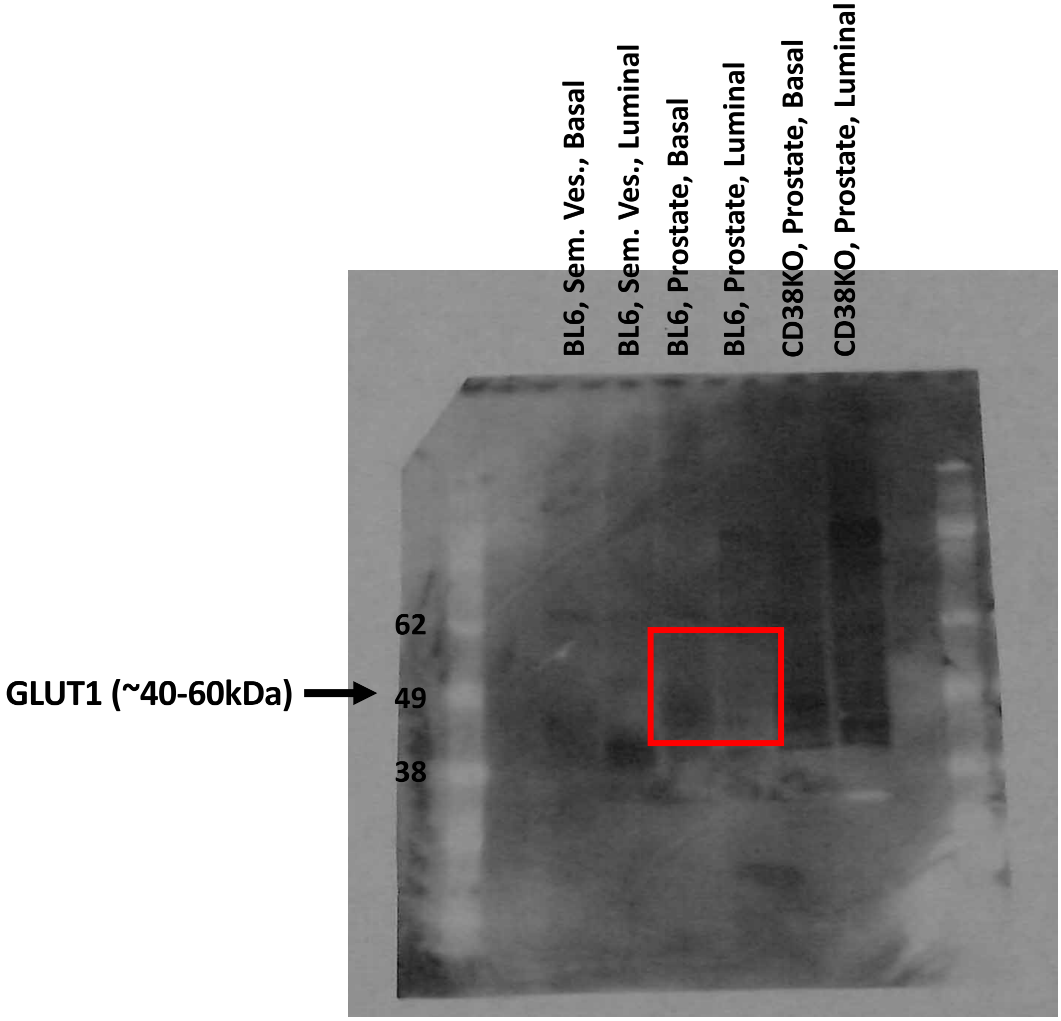

Slot-blot – SYPRO Ruby

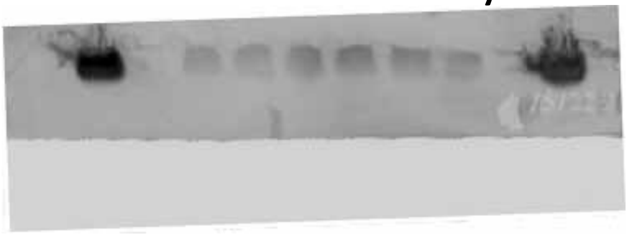

Extended Data Figure 1f

GLUT3

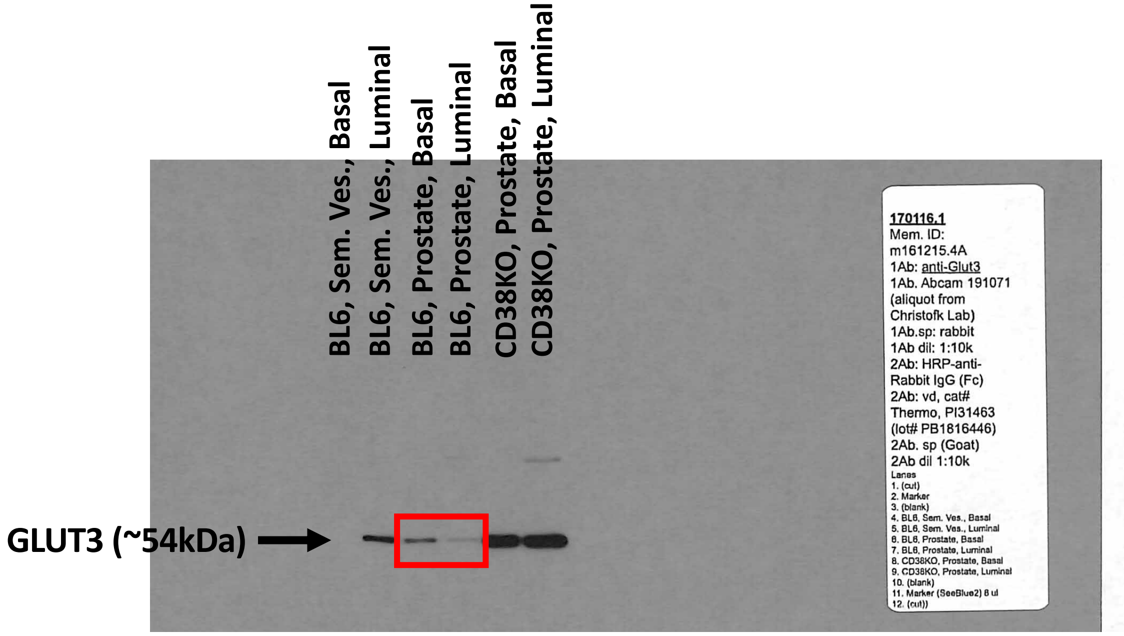

Slot-blot – SYPRO Ruby

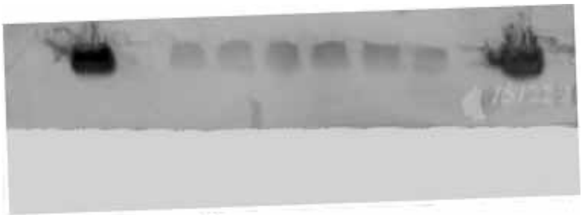

Extended Data Figure 1f

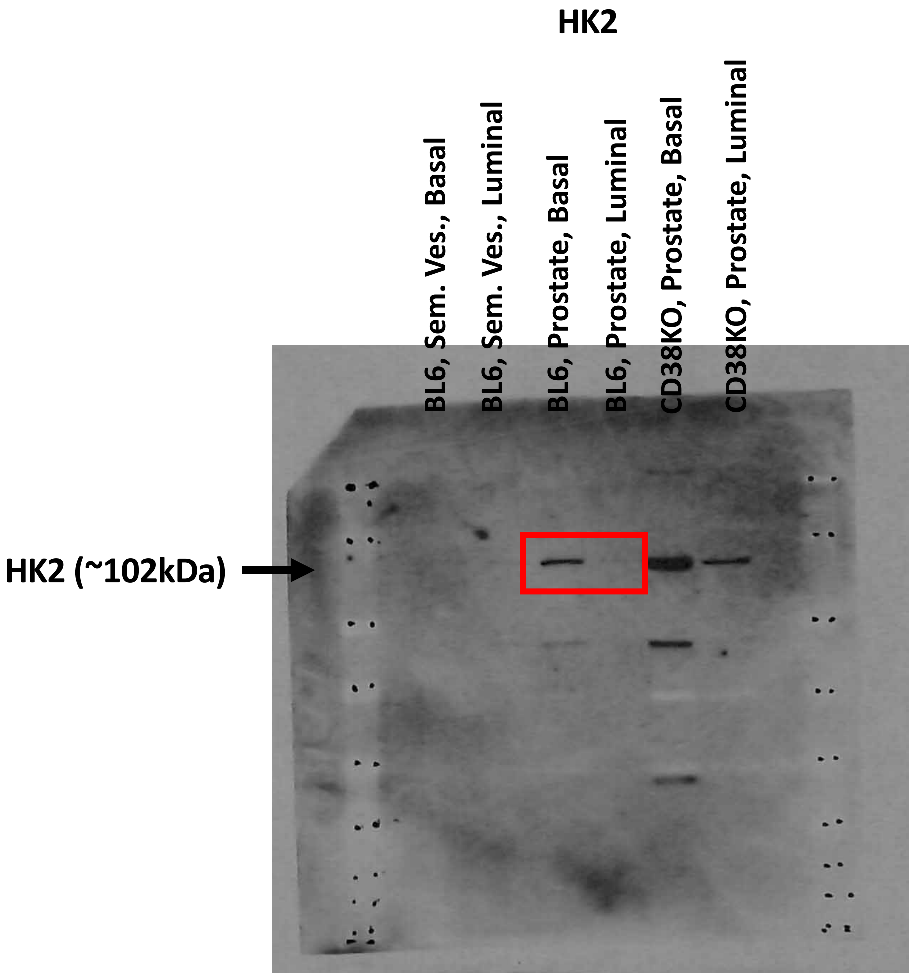

Slot-blot – SYPRO Ruby

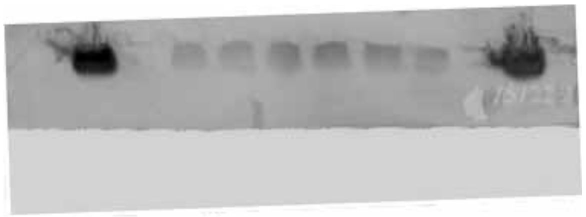

Extended Data Figure 1f

PFKP

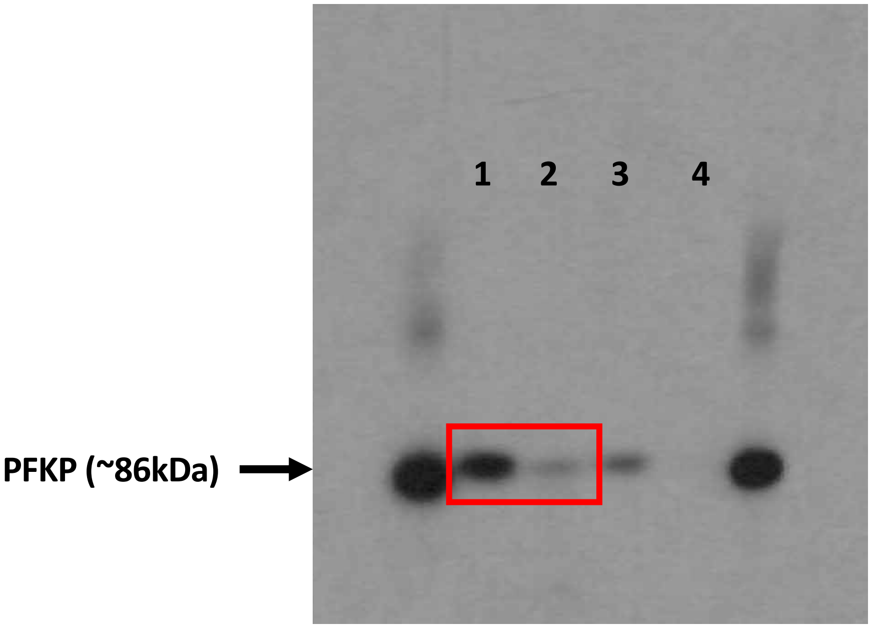

1. 12/5/16-3: WT(BL6) Prostate Basal 2.5 ug
2. 12/5/16-4: WT(BL6) Prostate Luminal 2.5 ug
3. 1/18/17-3: WT(BL6) Prostate Basal 2.5 ug
4. 1/18/17-4: WT(BL6) Prostate Luminal 2.5 ug

SYPRO Ruby

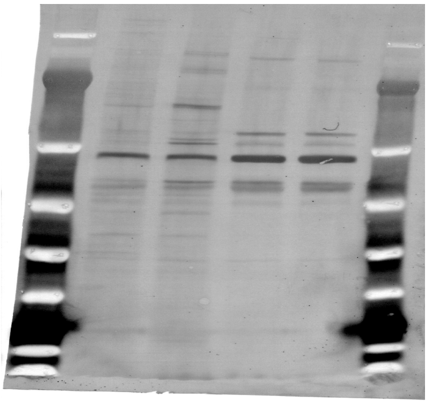

Extended Data Figure 1f

PCX

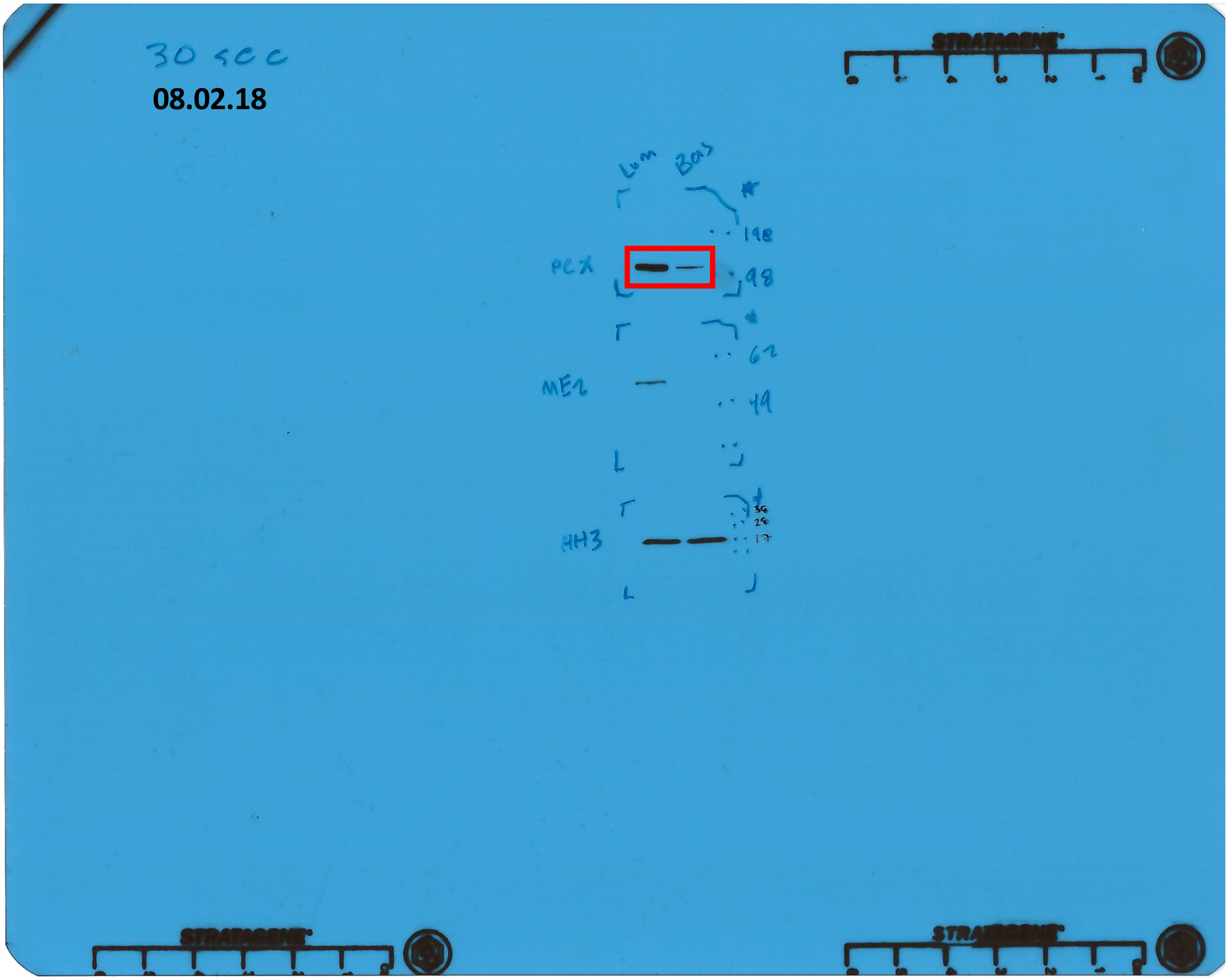

Extended Data Figure 1f

PDHA1

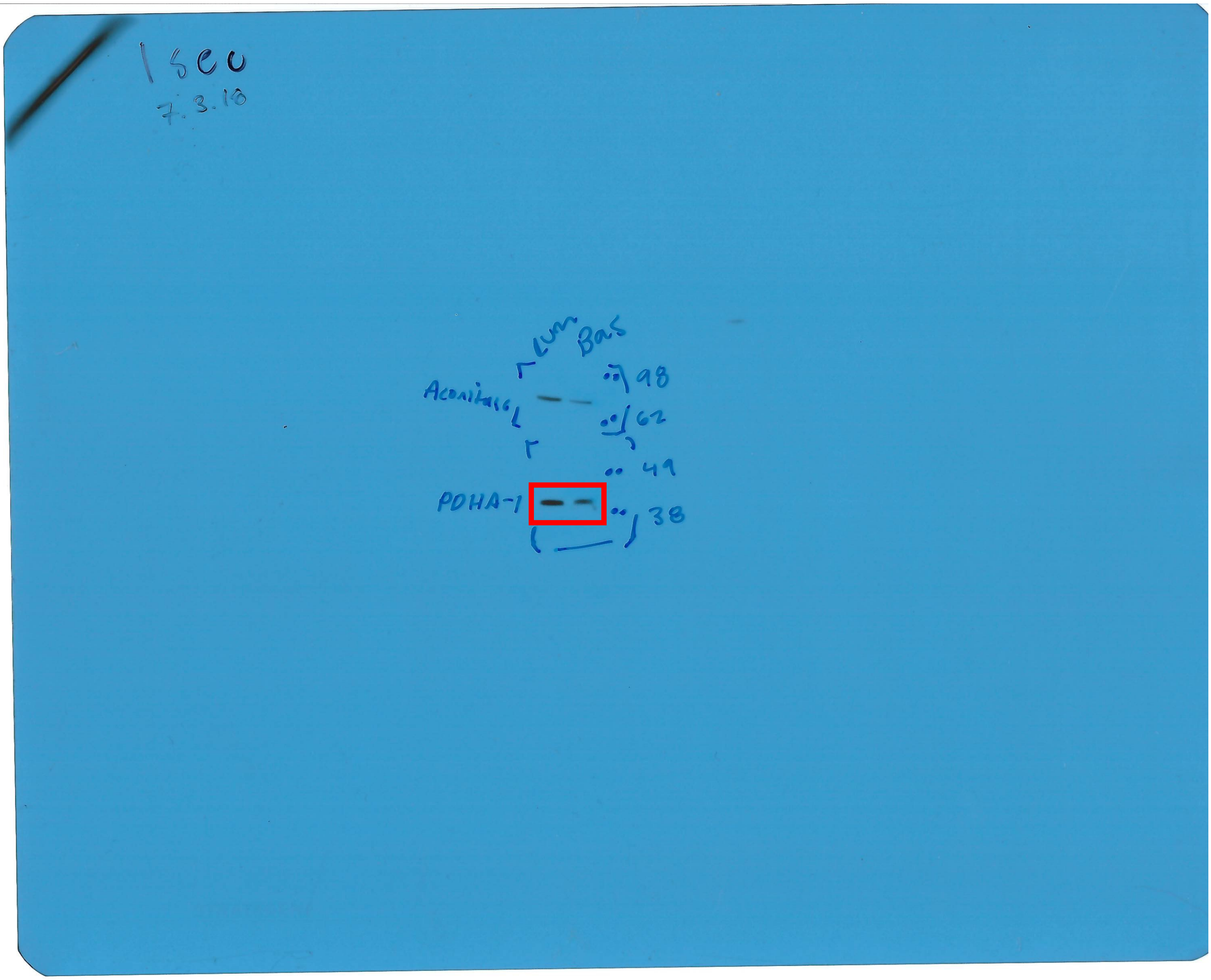

Extended Data Figure 1f

ACO2

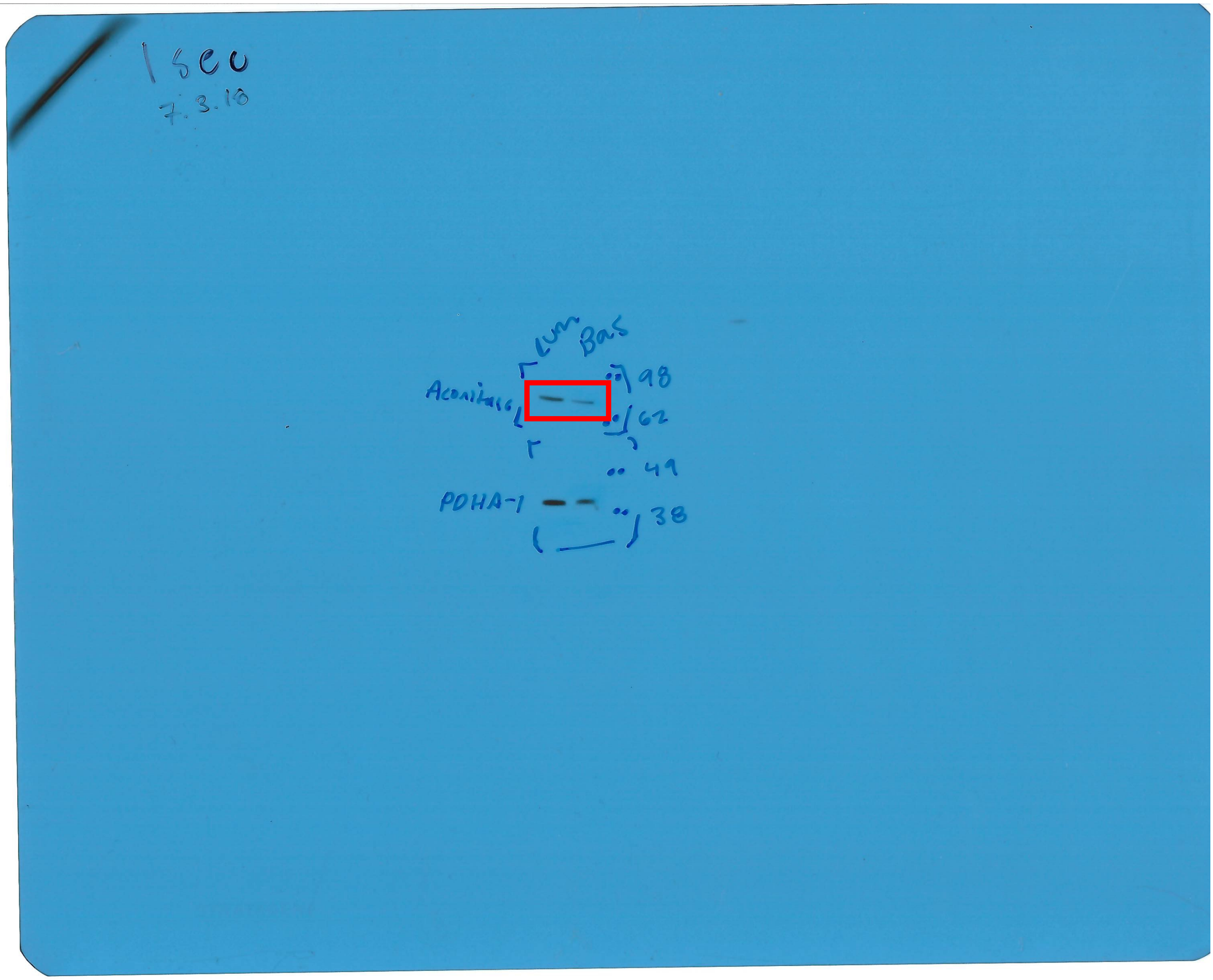

Extended Data Figure 1f

HH3

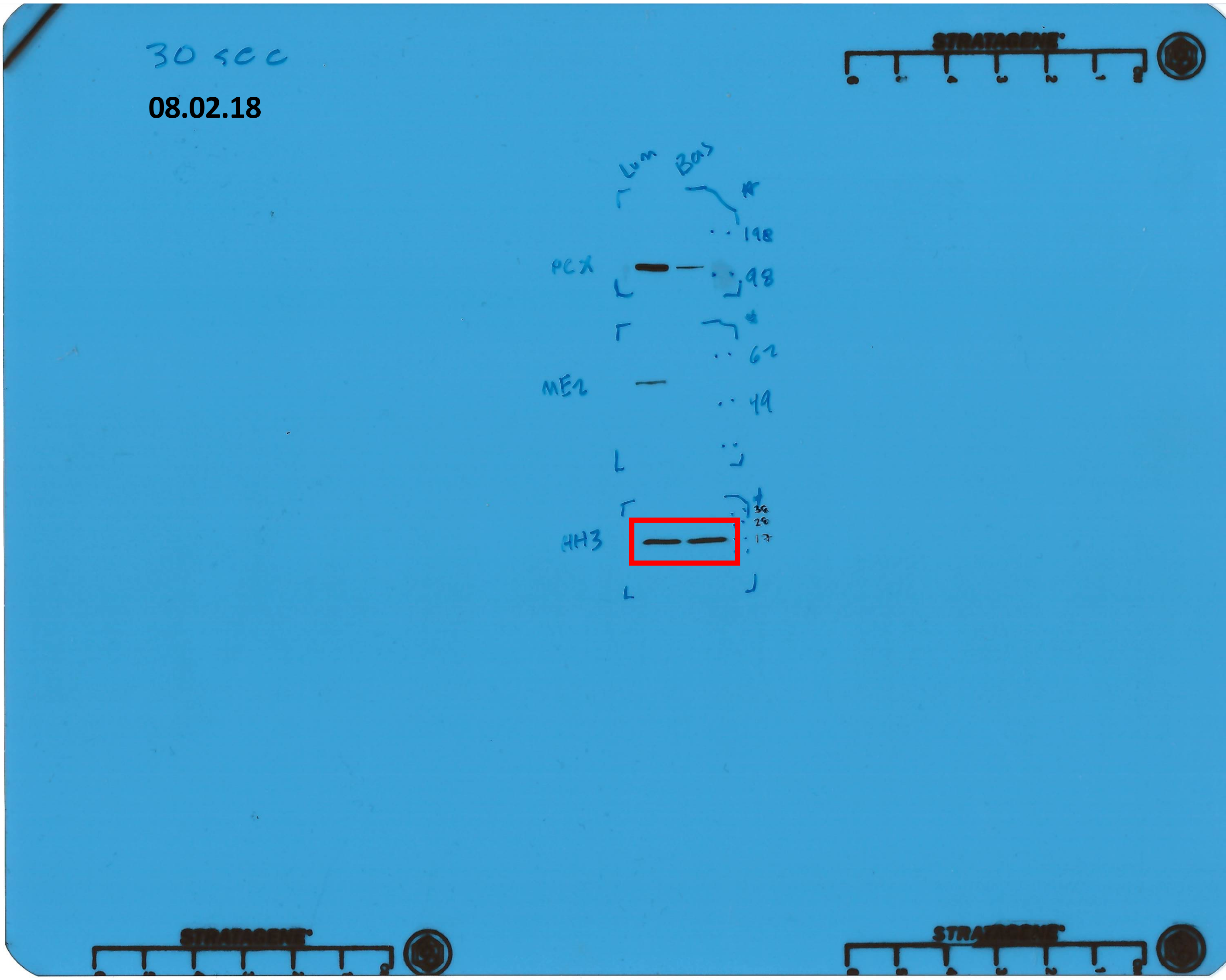

Extended Data Figure 3f

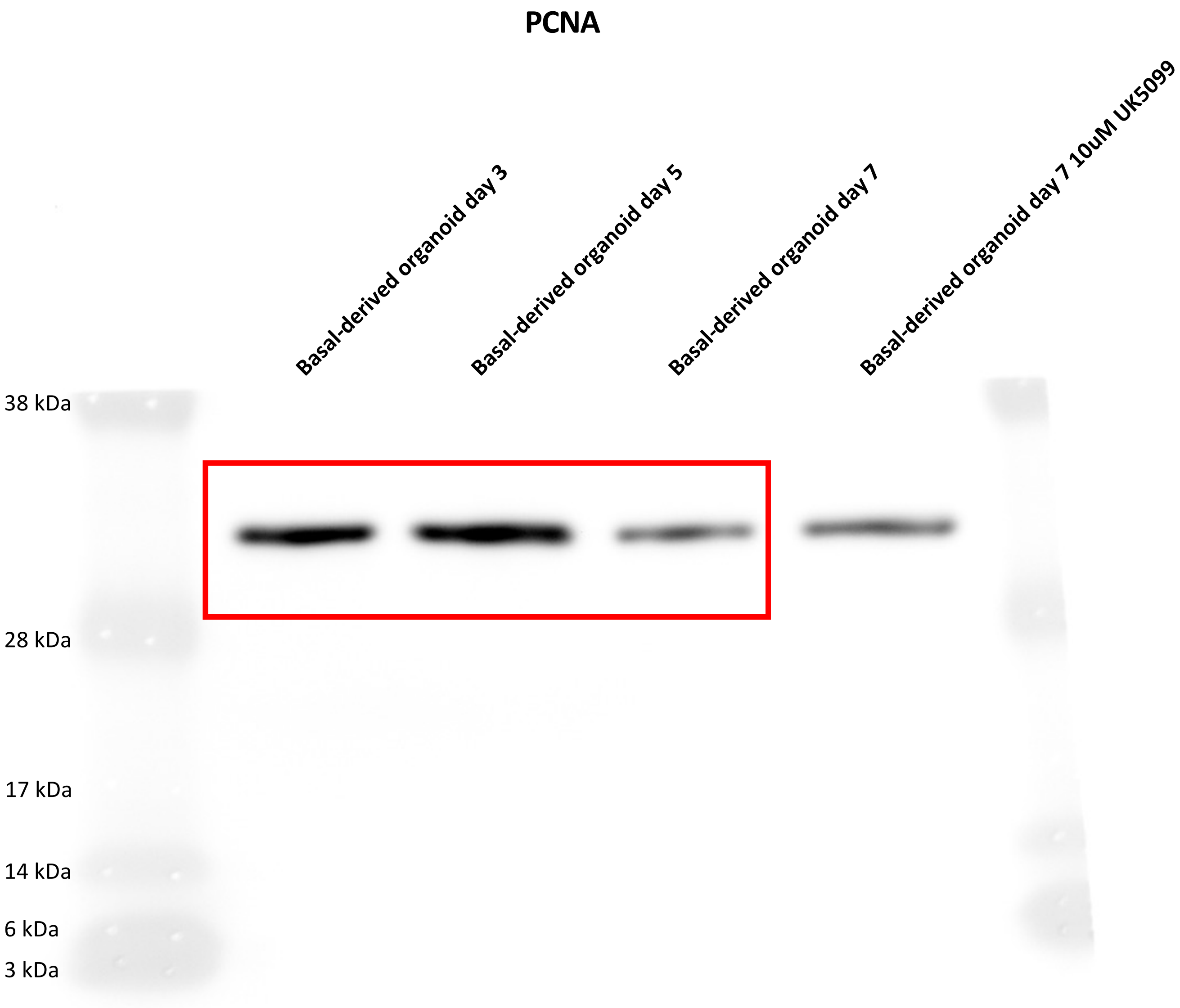

Extended Data Figure 3f

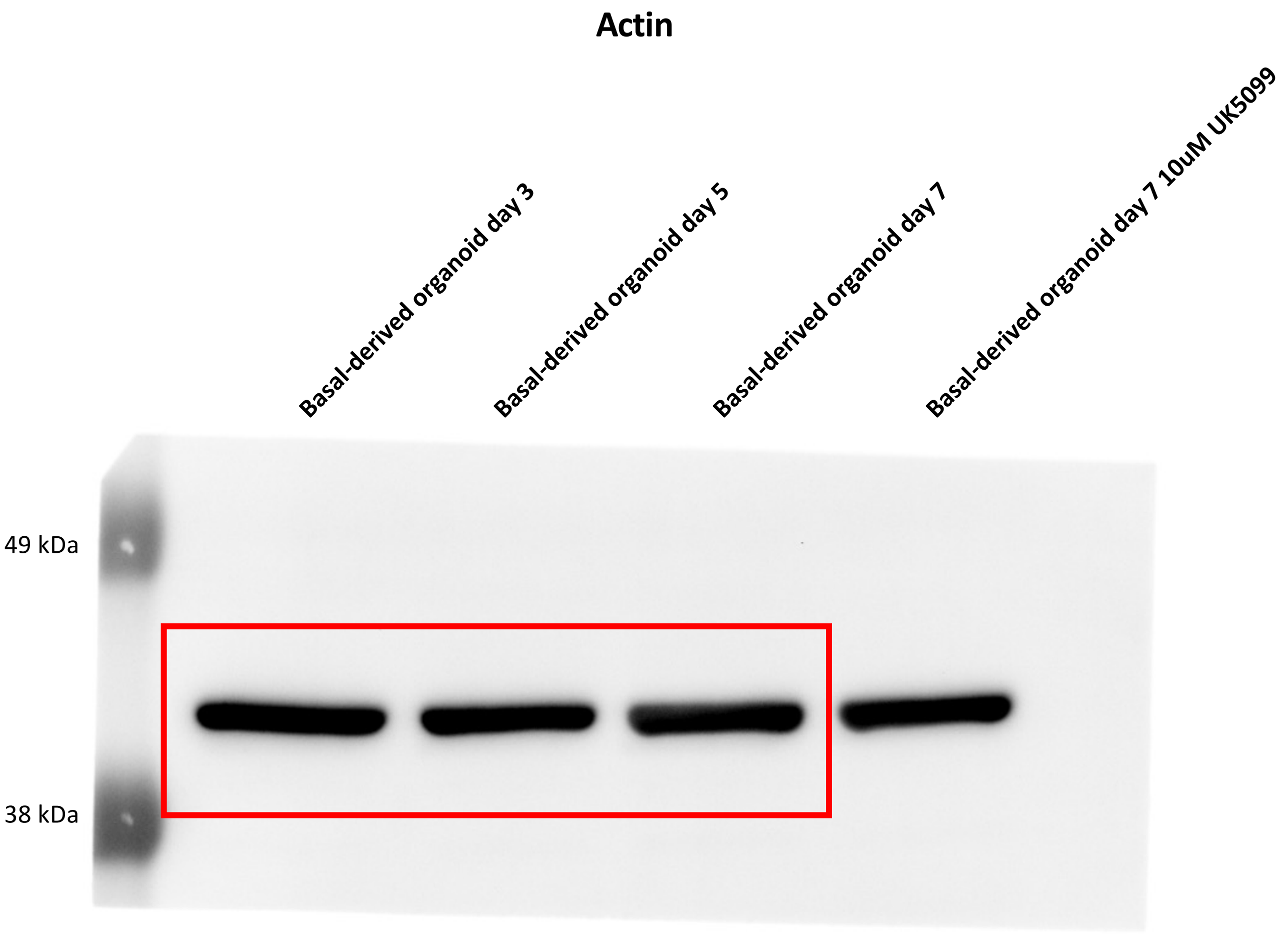

Extended Data Figure 4d

Ki67

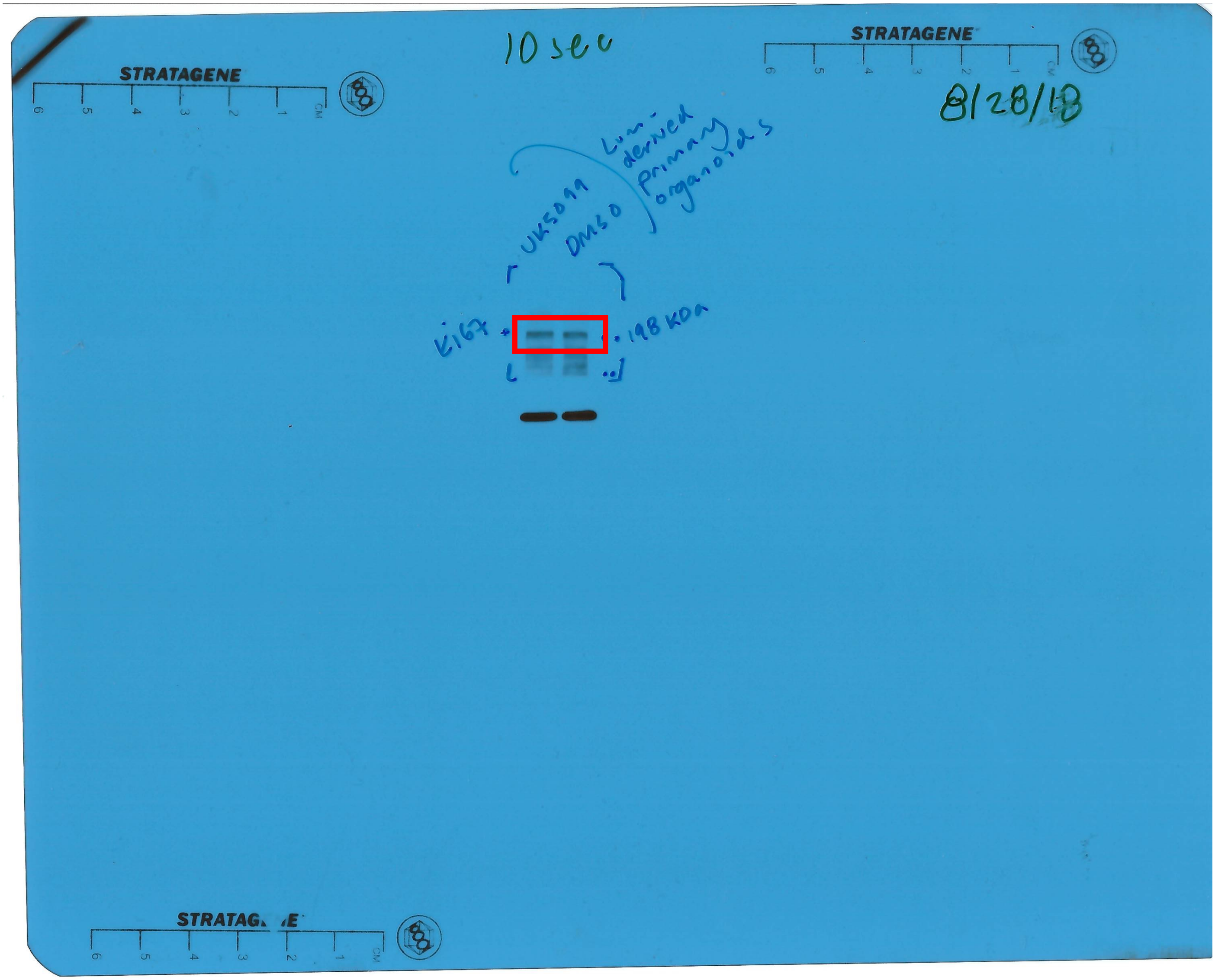

Extended Data Figure 4d

PCNA

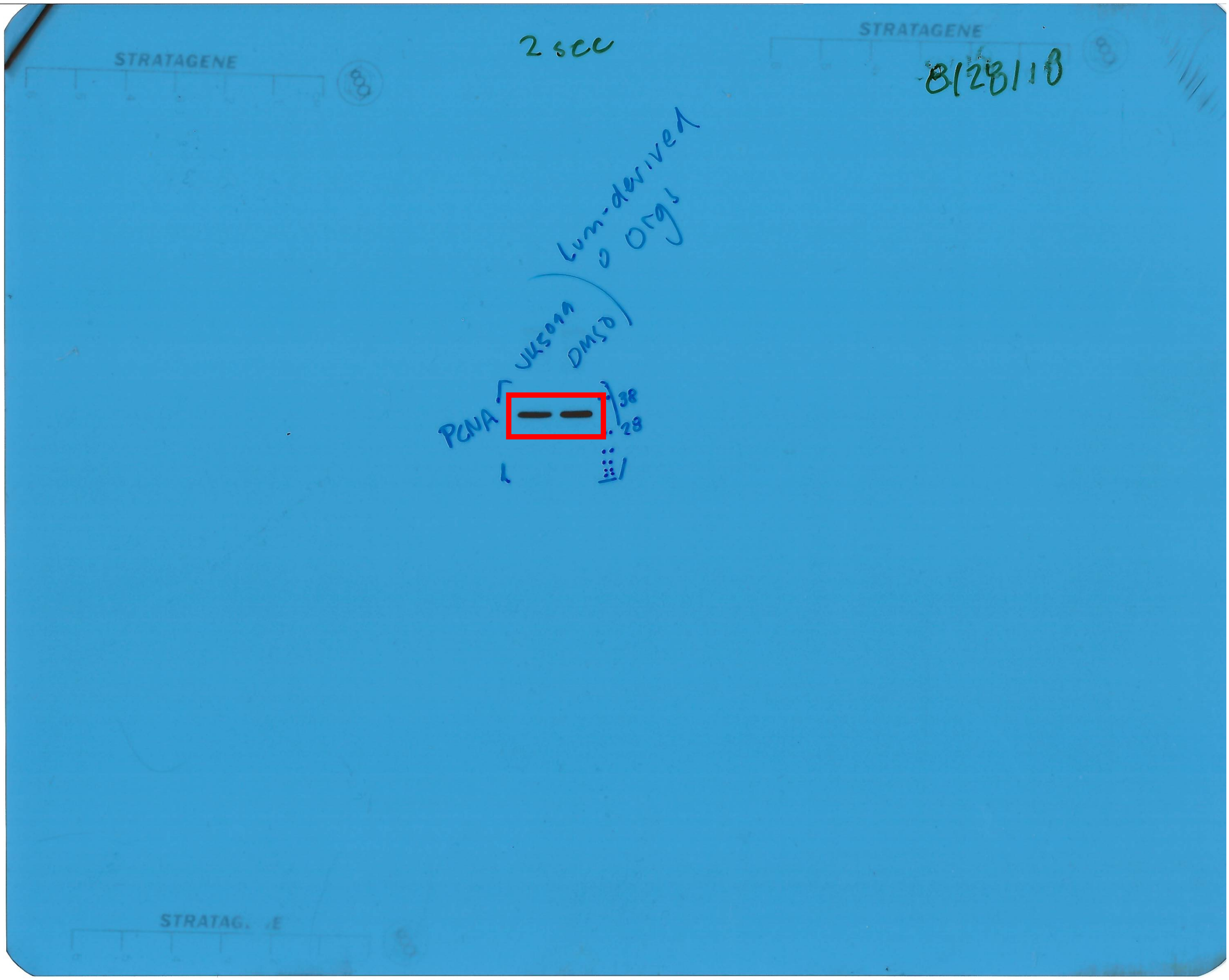

Extended Data Figure 4d

Cleaved caspase-3

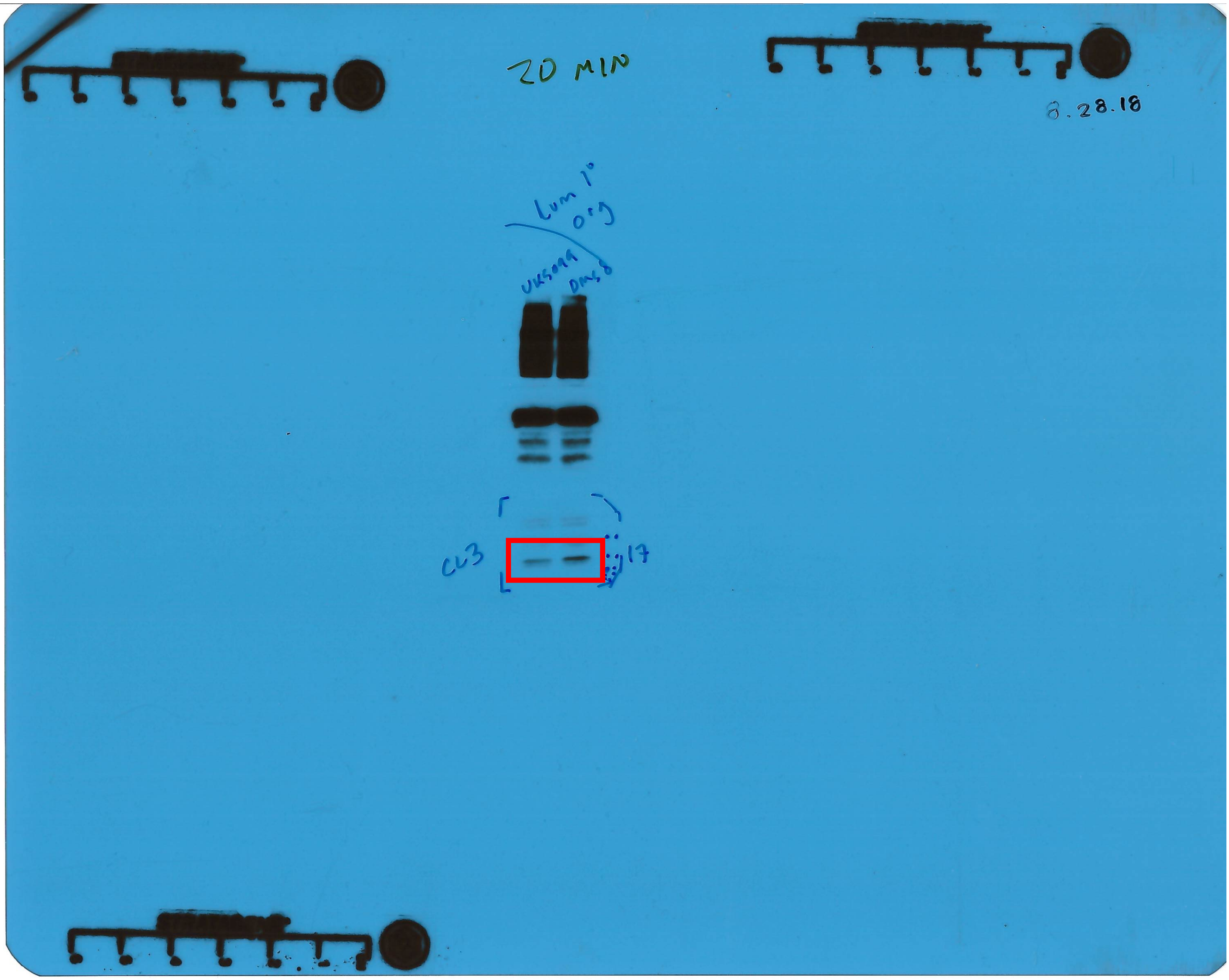

Extended Data Figure 4d

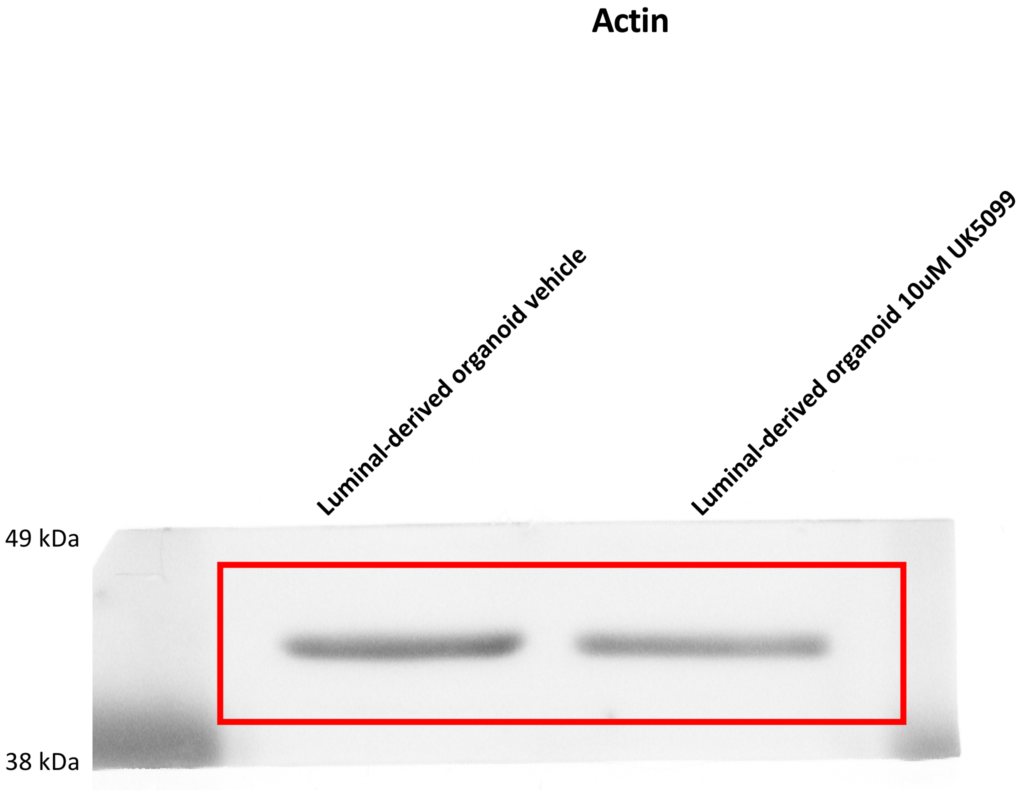

Extended Data Figure 6a

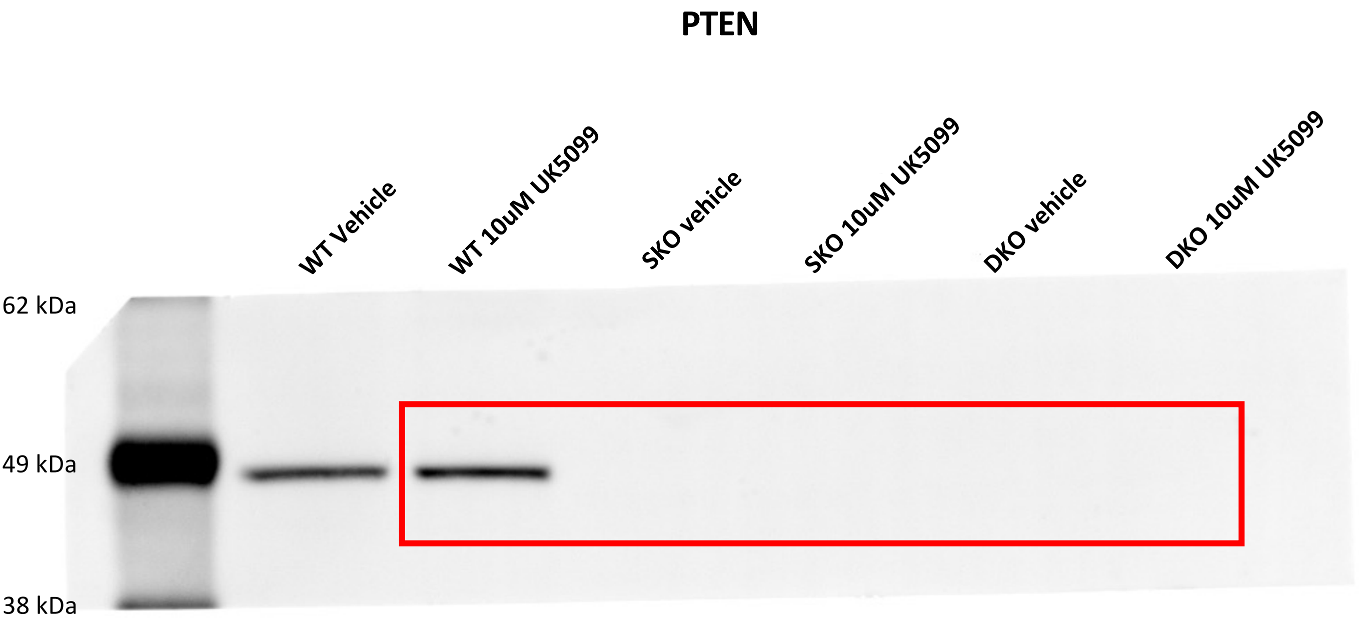

Extended Data Figure 6a

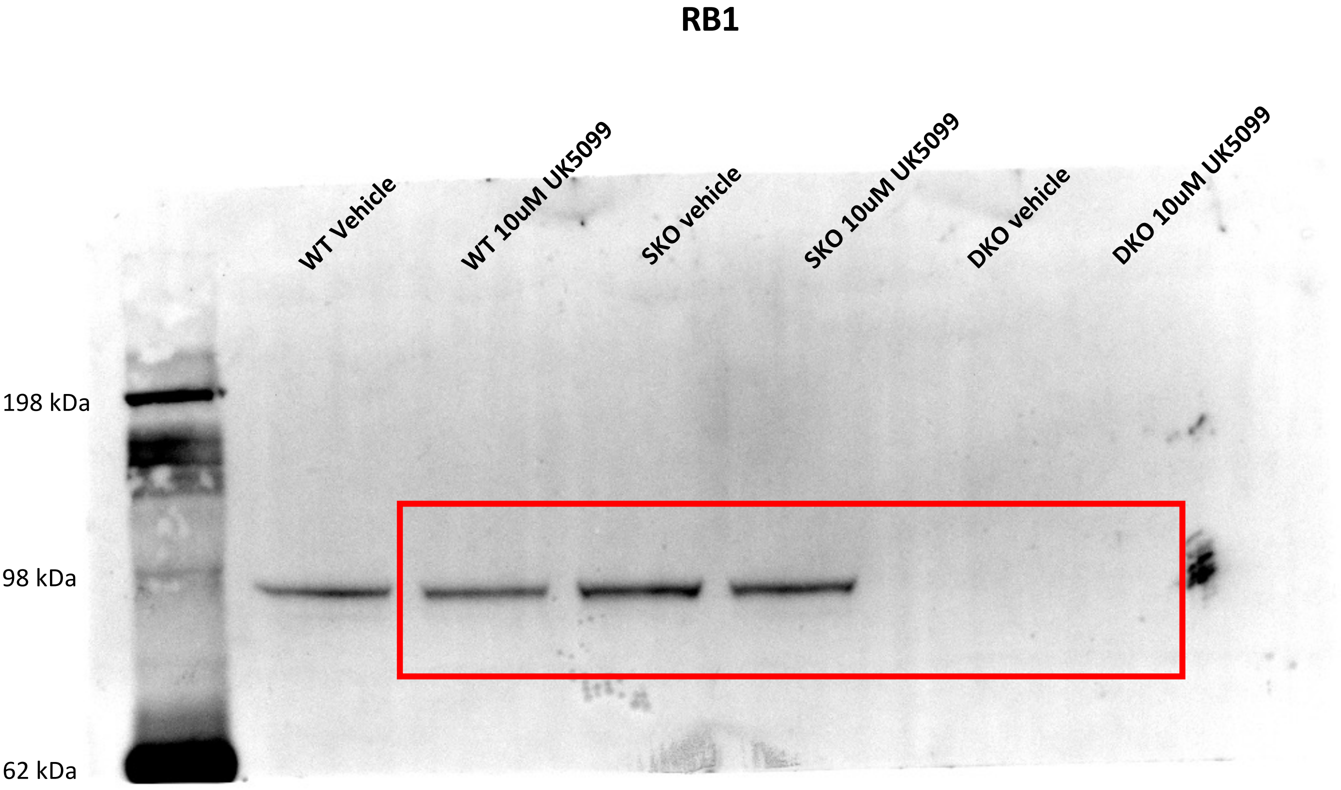

Extended Data Figure 6a

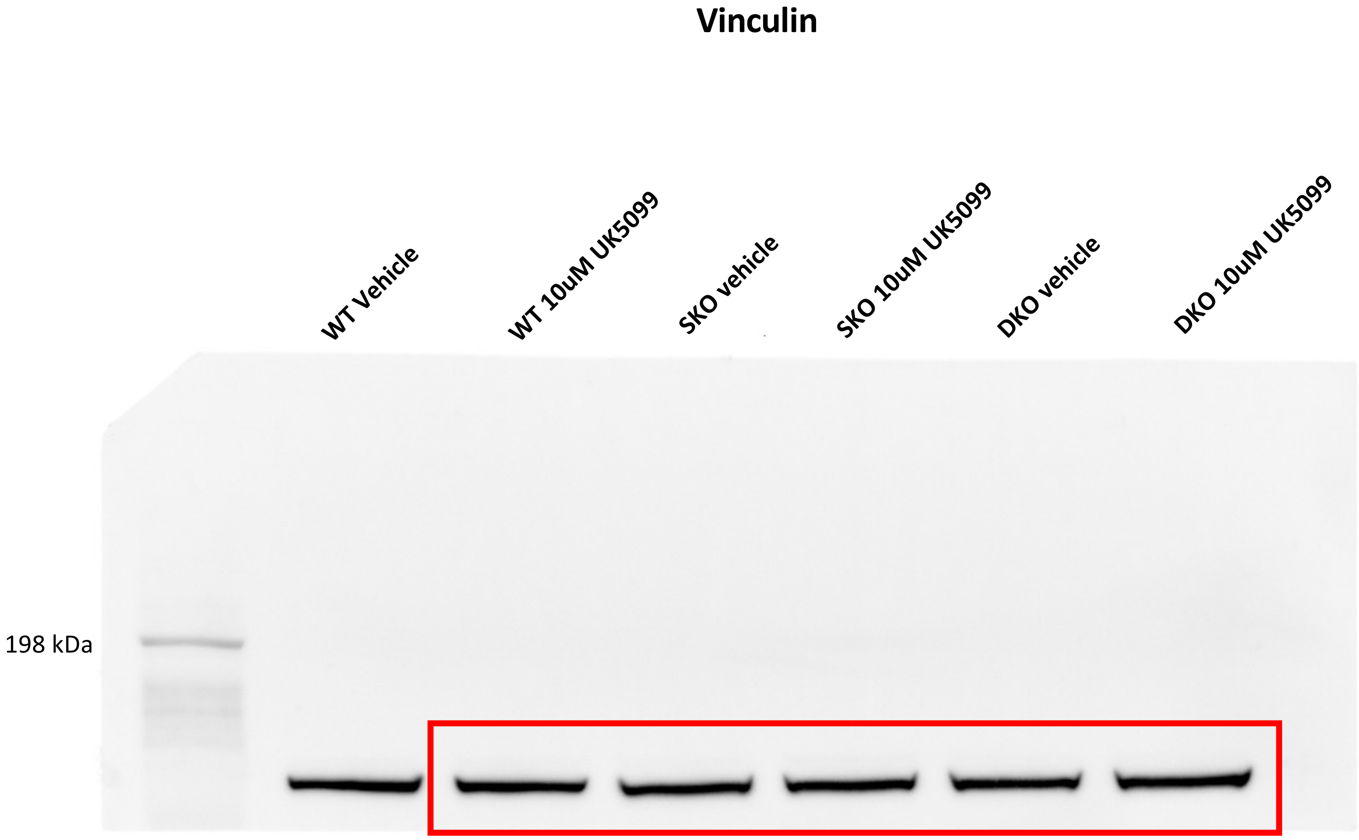

Extended Data Figure 6e

AR

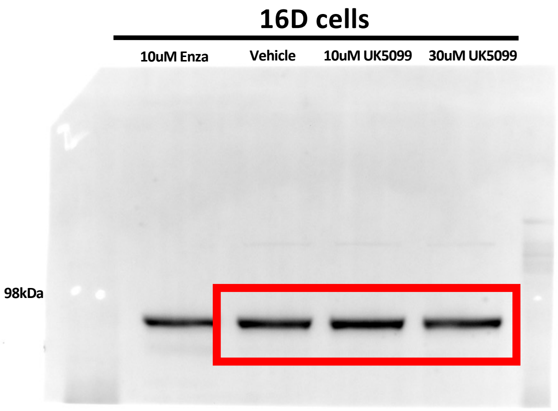

Extended Data Figure 6e

PSA

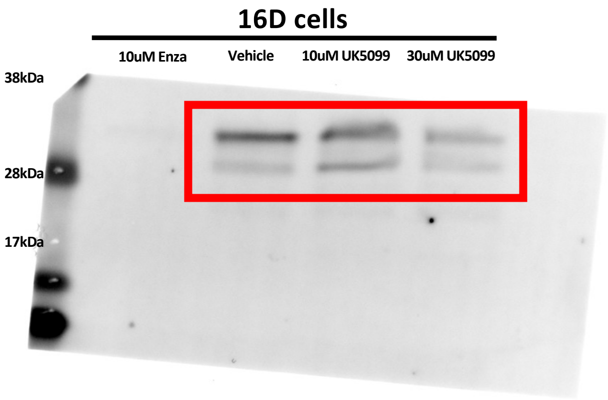

Extended Data Figure 6e

K8

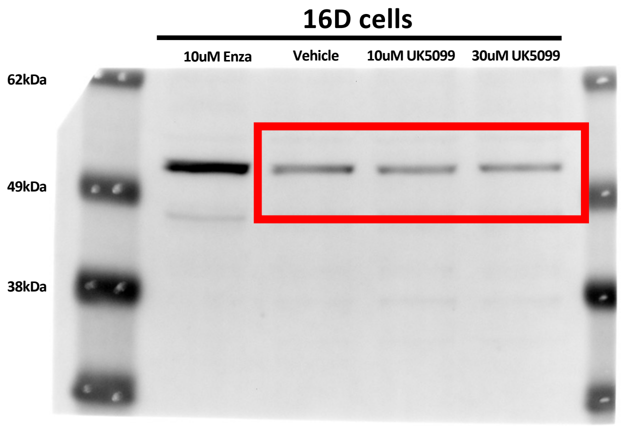

Extended Data Figure 6e

K18

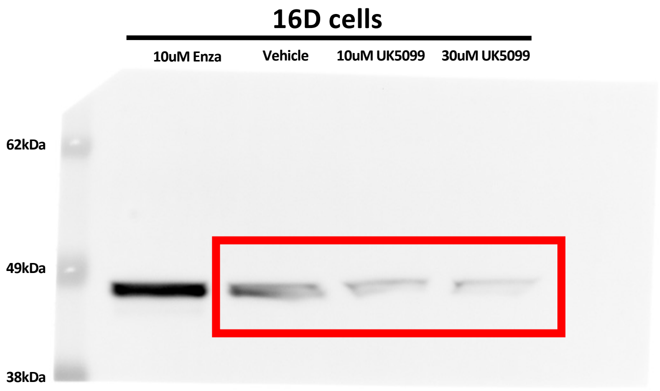

Extended Data Figure 6e

NSE

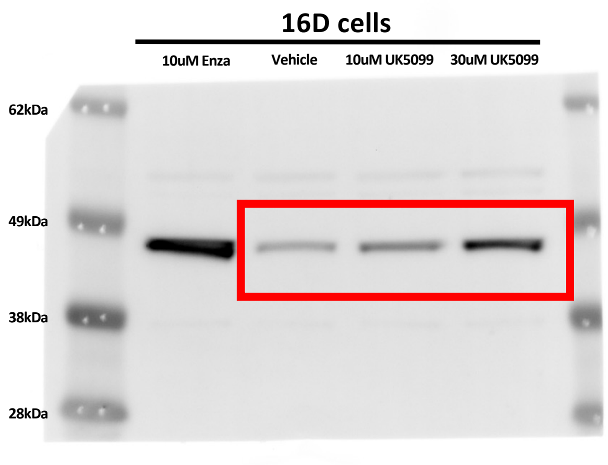

Extended Data Figure 6e

SYP

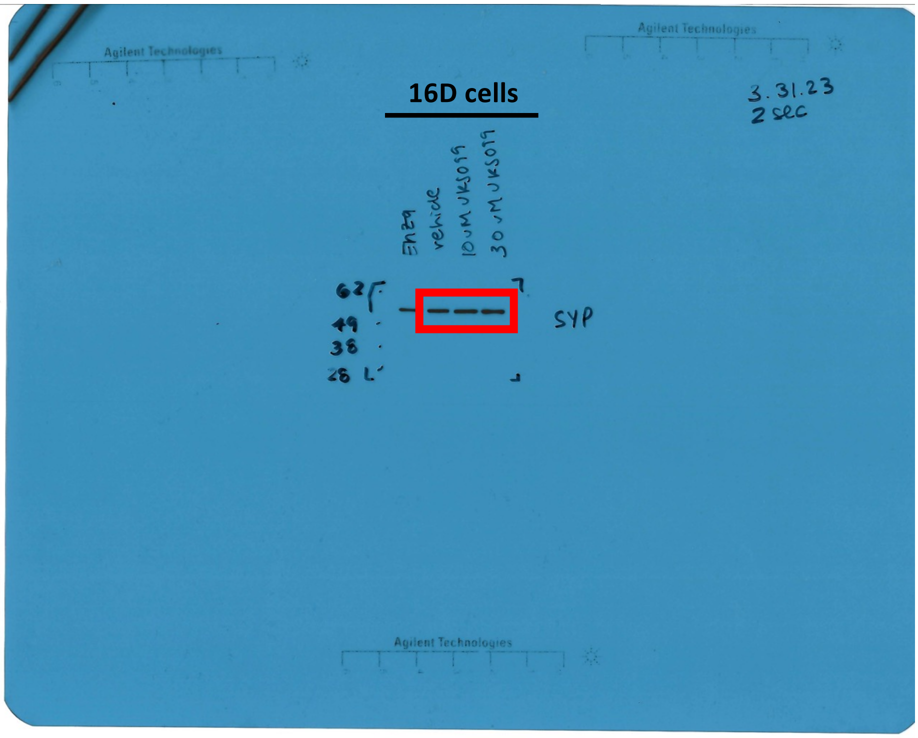

Extended Data Figure 6e

SOX2

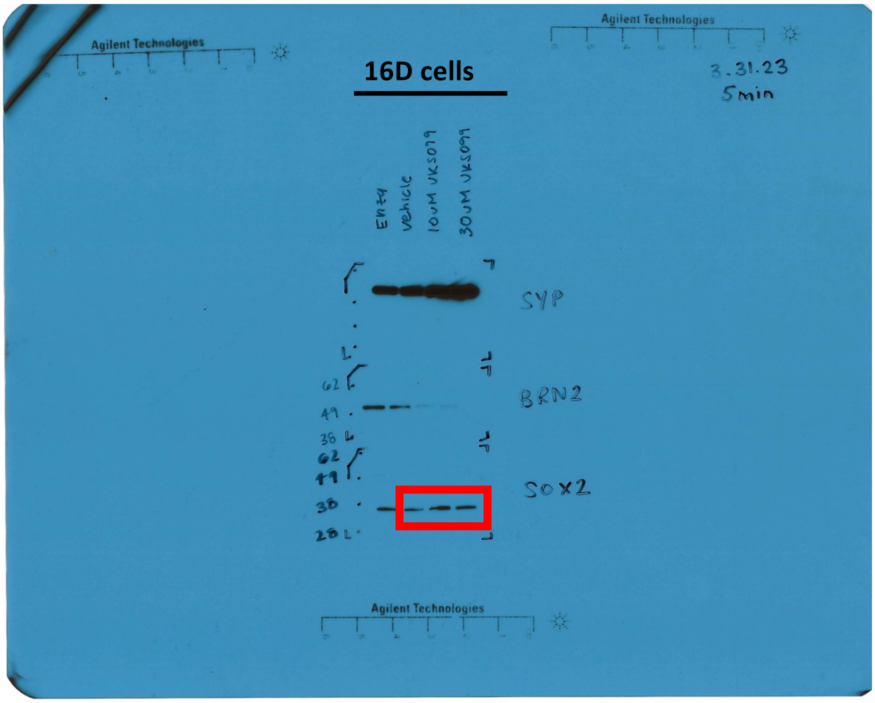

Extended Data Figure 6e

Actin

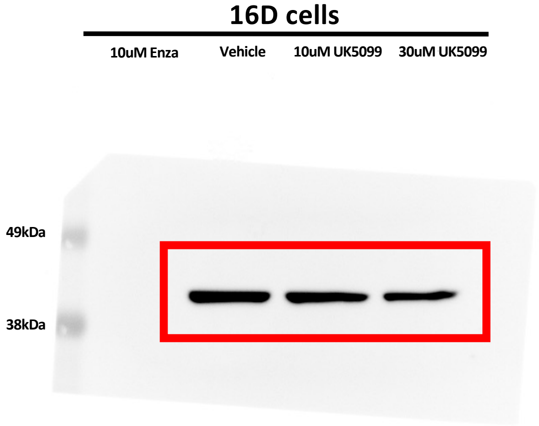

Extended Data Figure 6f

AR

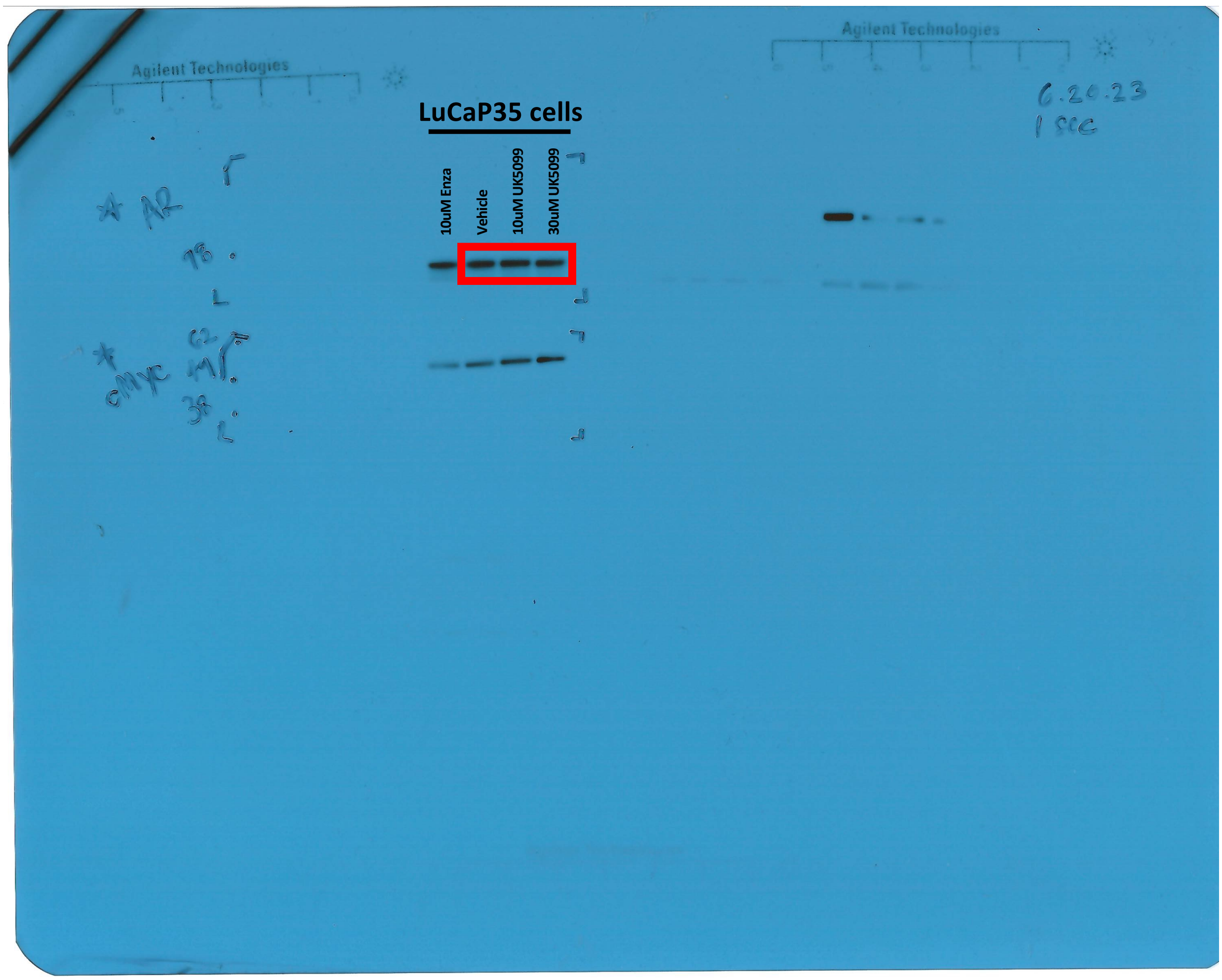

Extended Data Figure 6f

KRT8

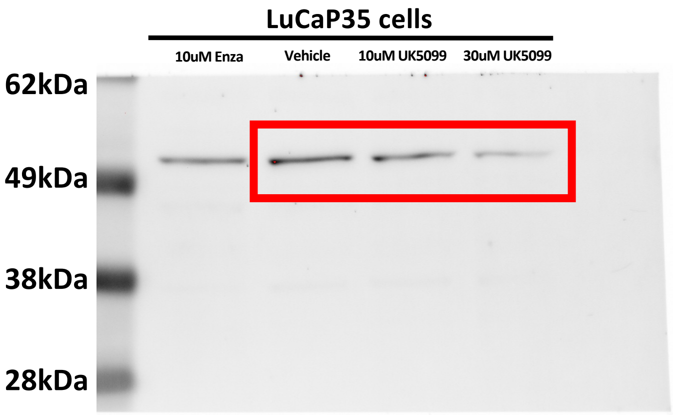

Extended Data Figure 6f

SOX2

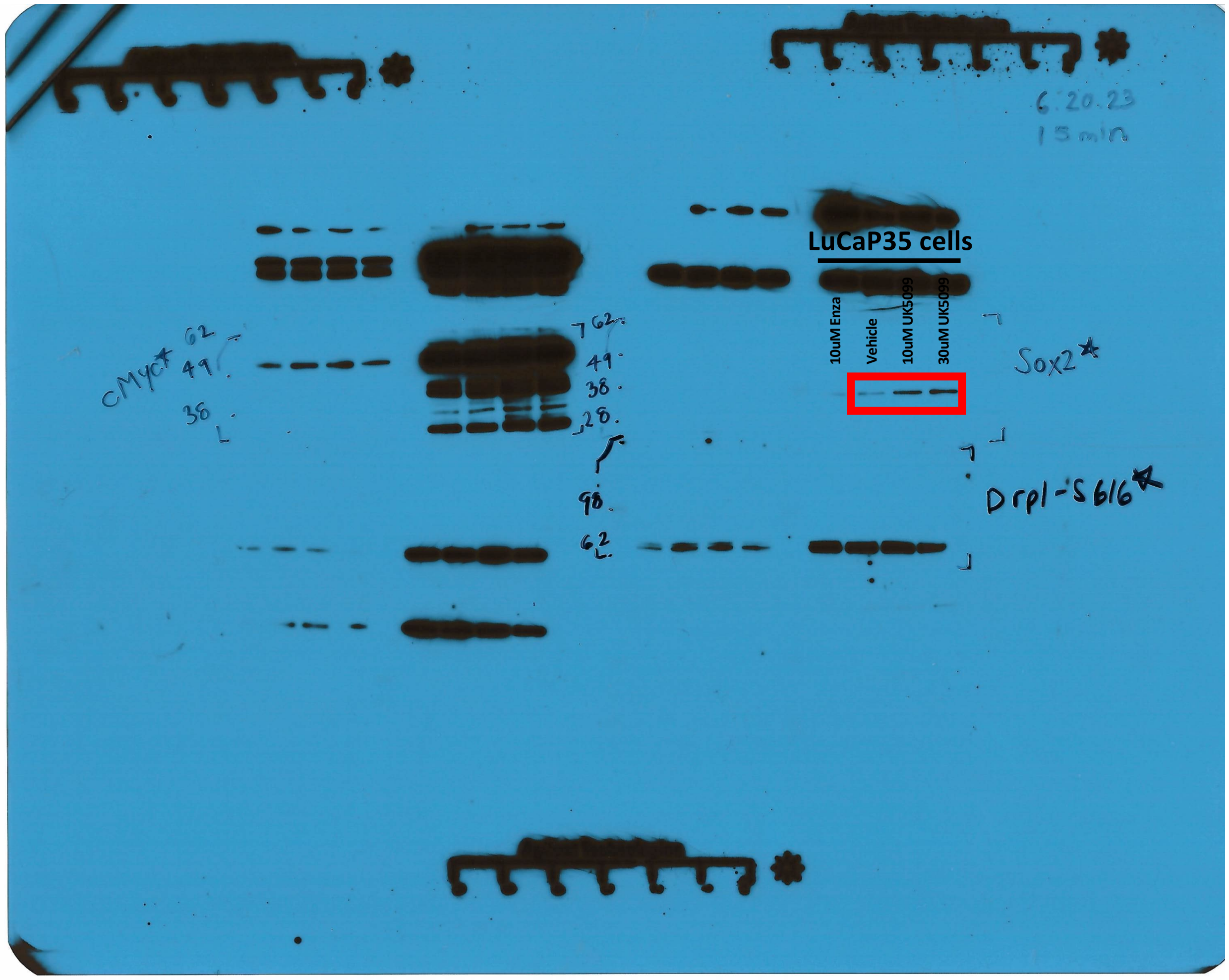

Extended Data Figure 6f

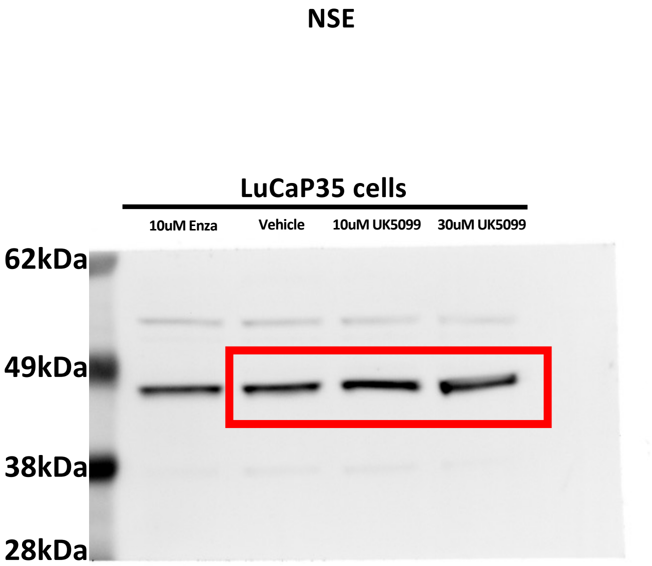

Extended Data Figure 6f

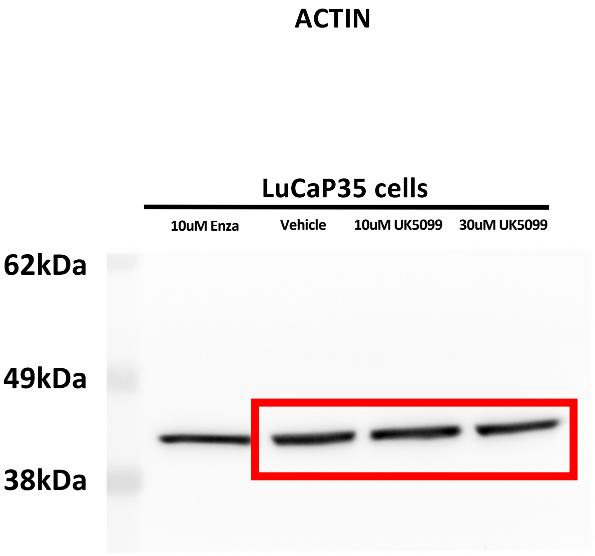

Extended Data Figure 6g

AR

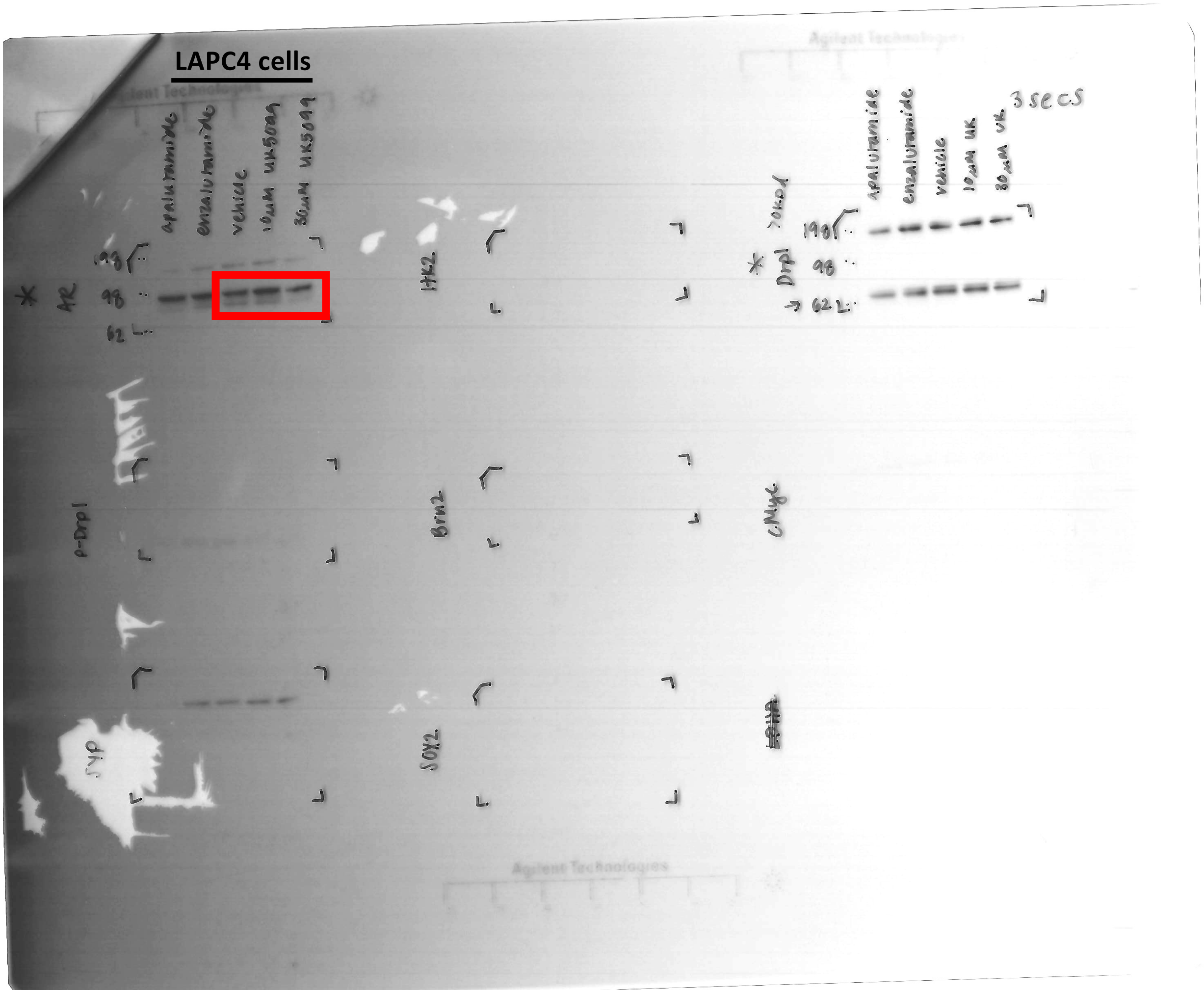

Extended Data Figure 6g

PSA

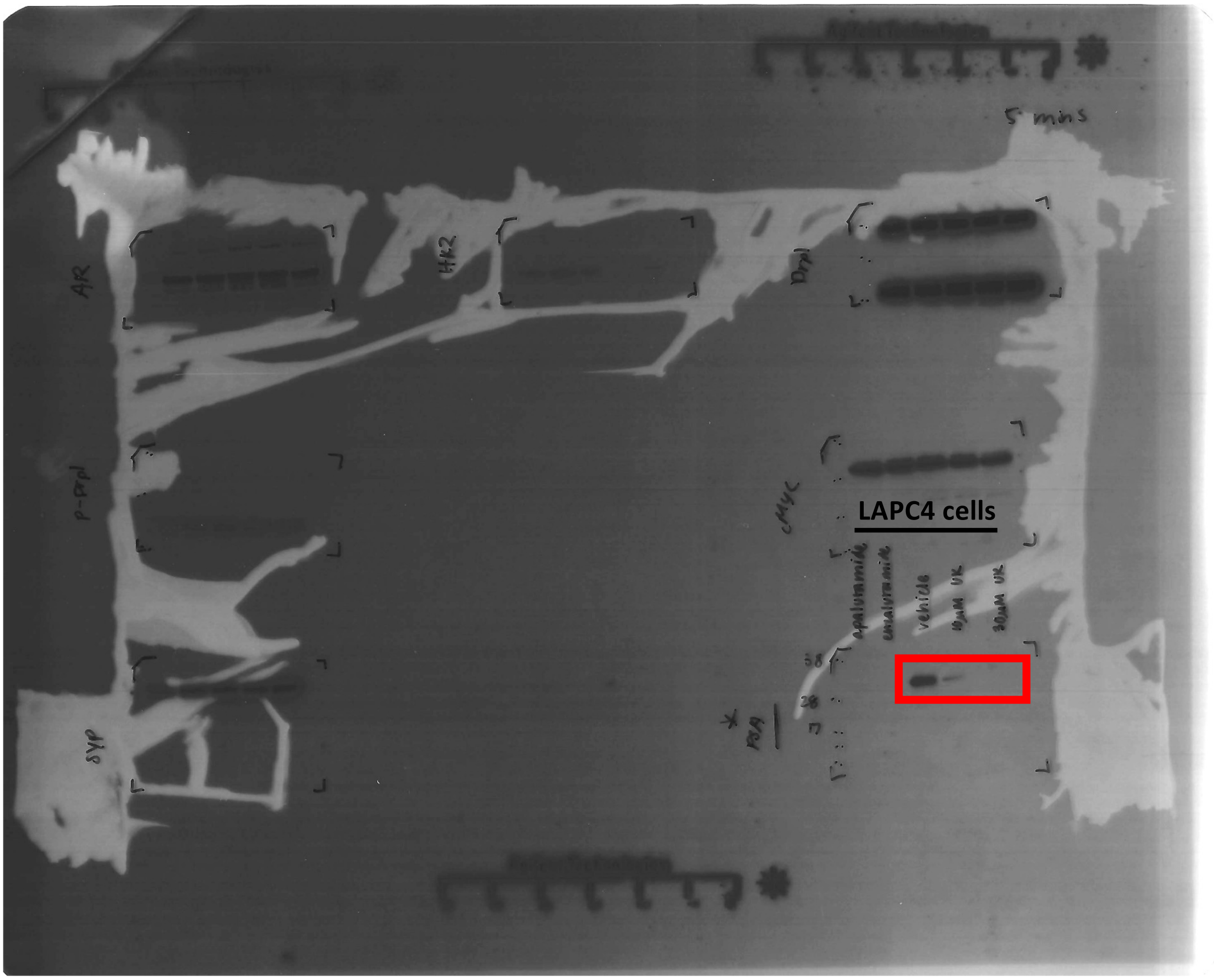

Extended Data Figure 6g

SYP

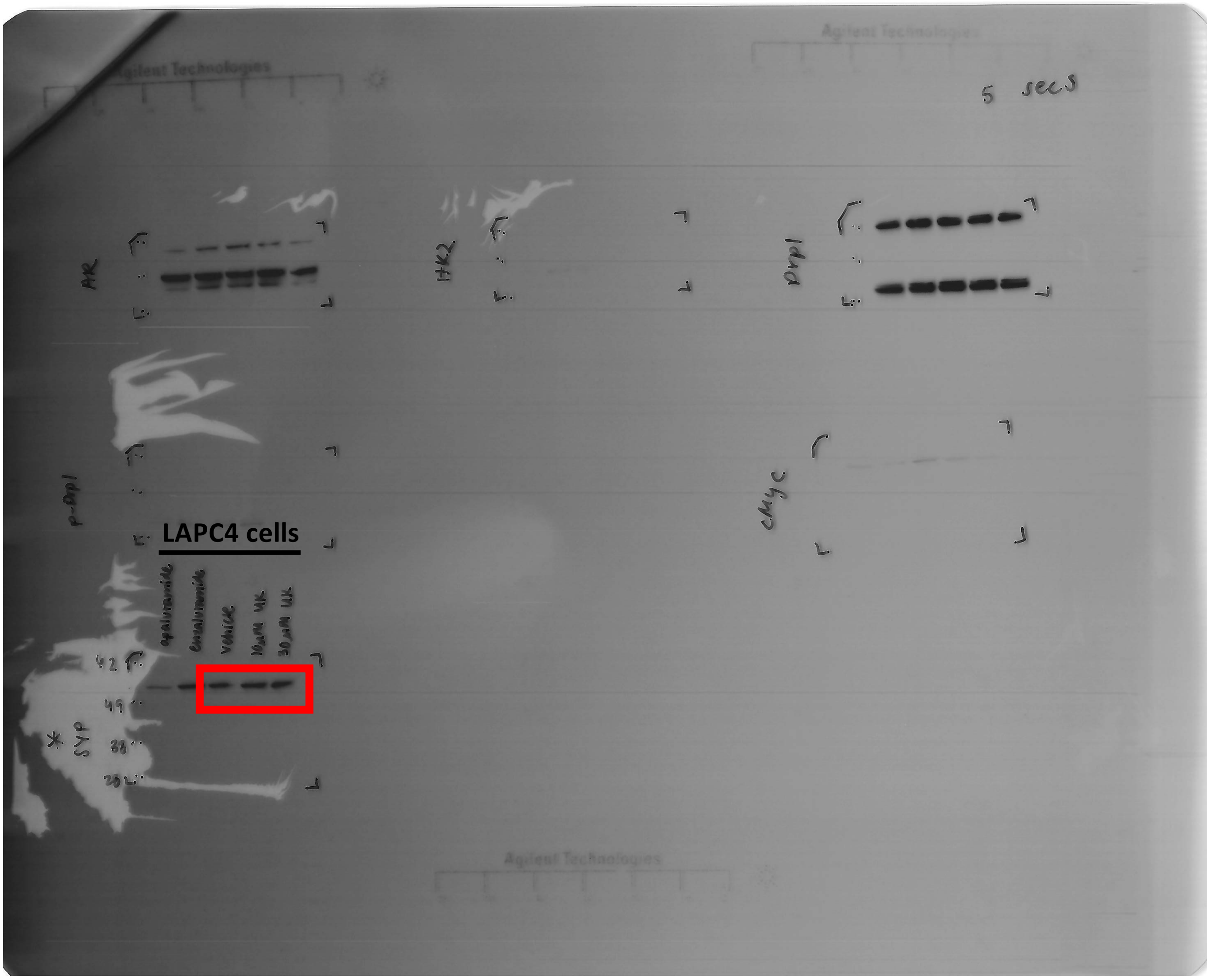

Extended Data Figure 6g

ACTIN

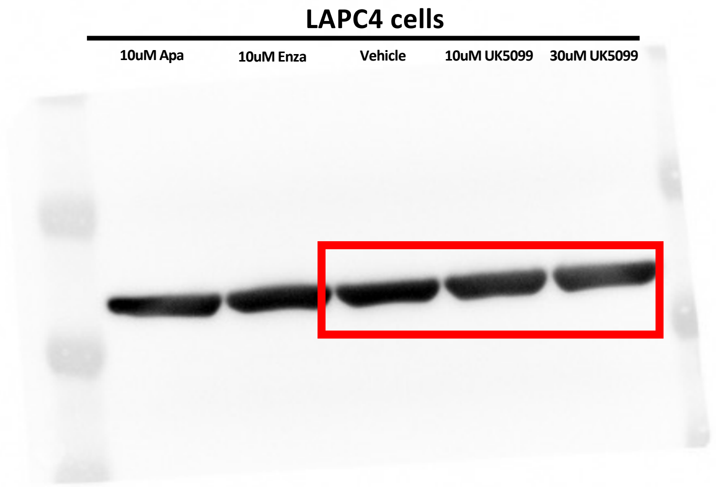

Extended Data Figure 6h

AR

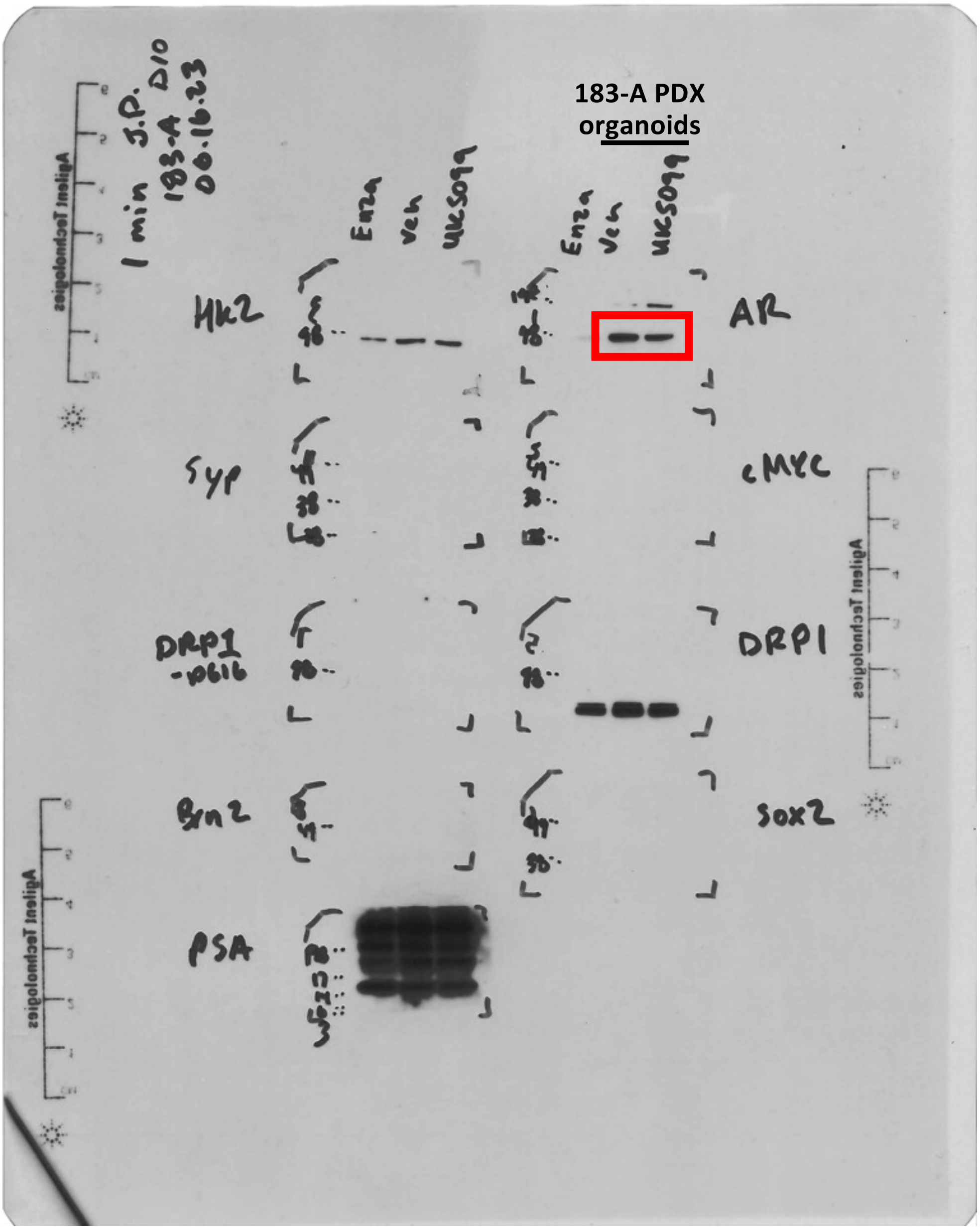

Extended Data Figure 6h

KRT8

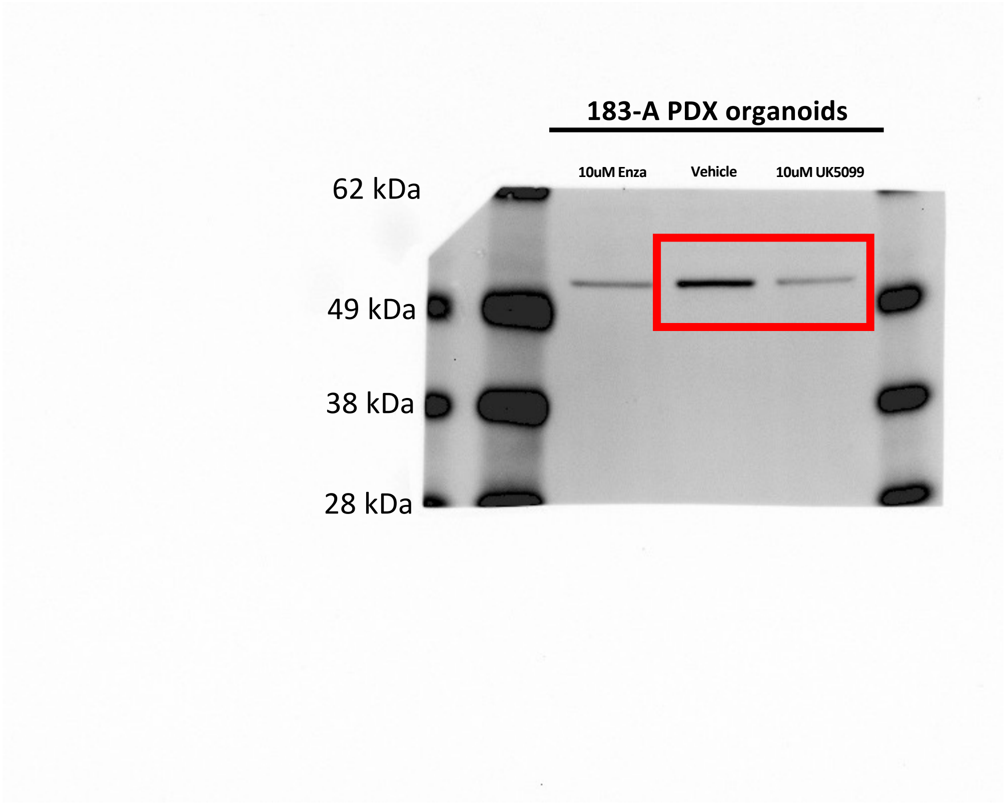

Extended Data Figure 6h

KRT18

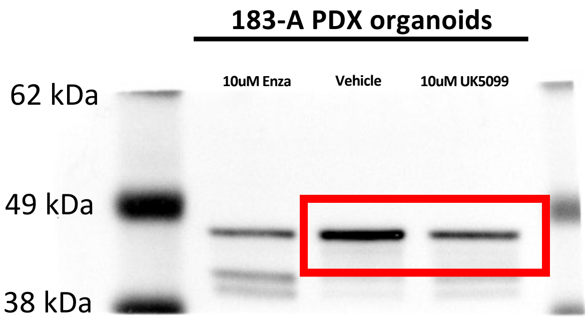

Extended Data Figure 6h

ACTIN

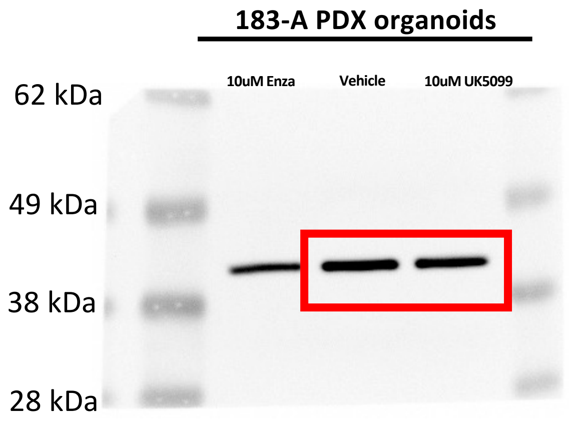

Extended Data Figure 6i

AR

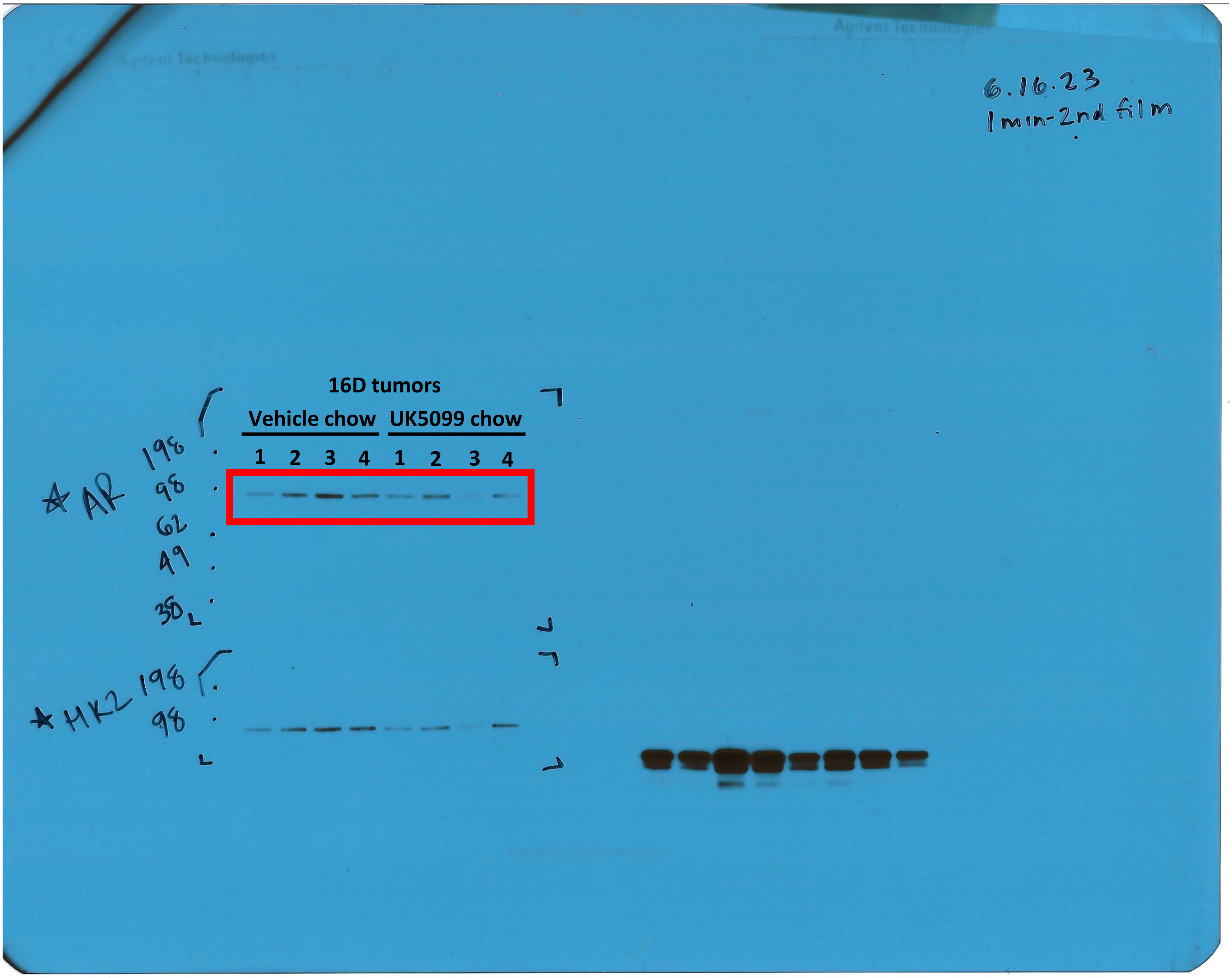

Extended Data Figure 6i

PSA

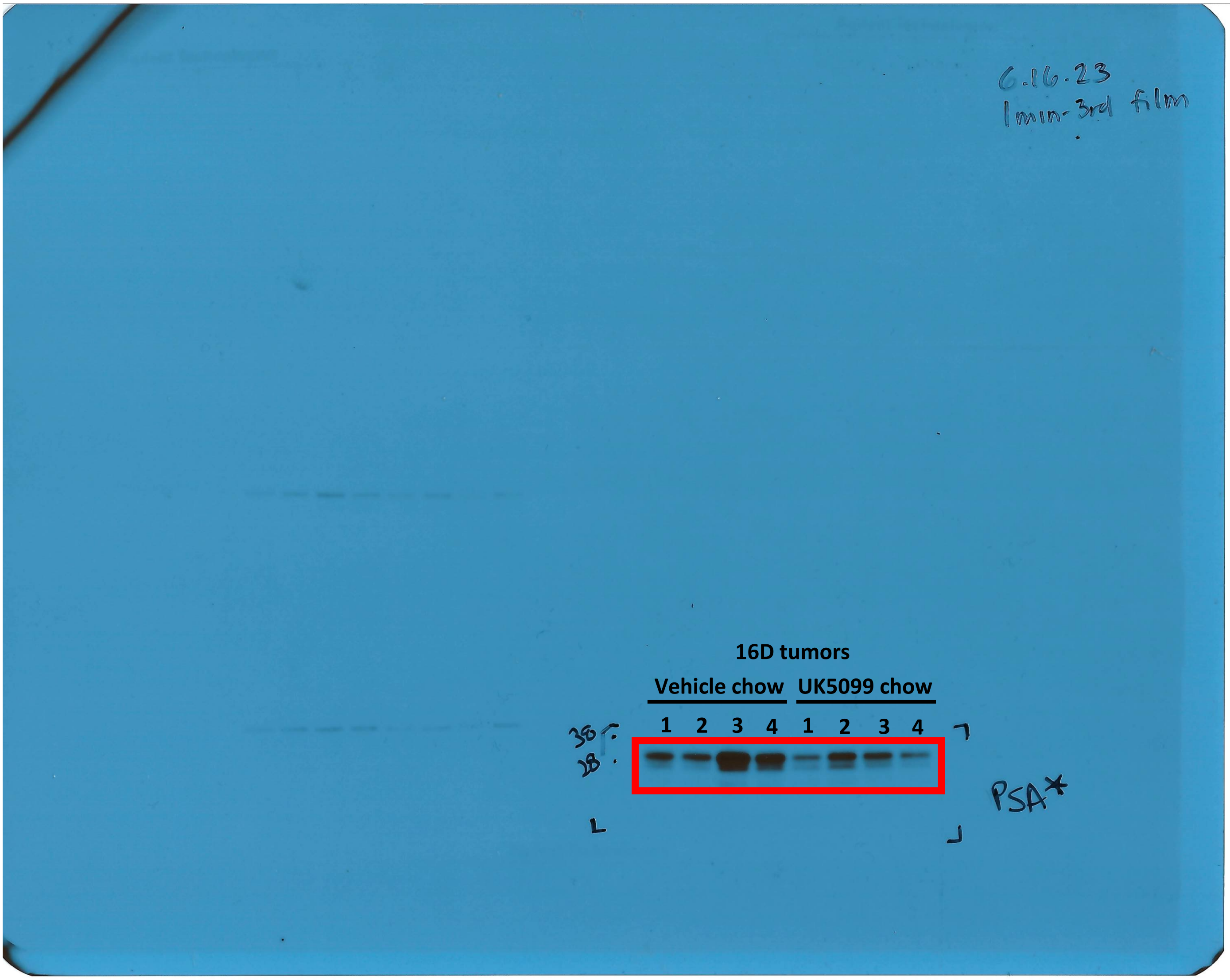

Extended Data Figure 6i

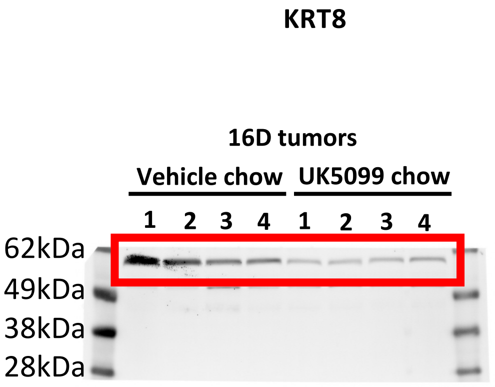

Extended Data Figure 6i

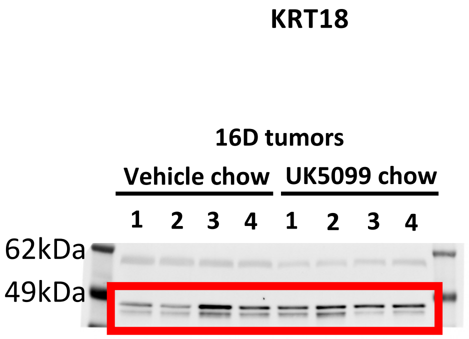

Extended Data Figure 6i

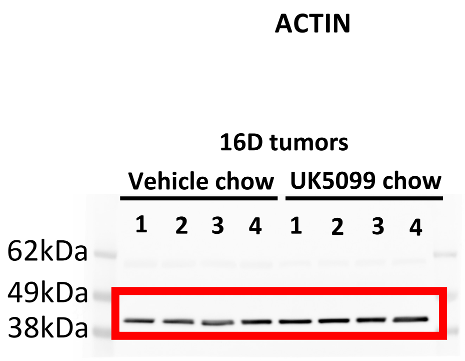

Extended Data Figure 7f

KRT8

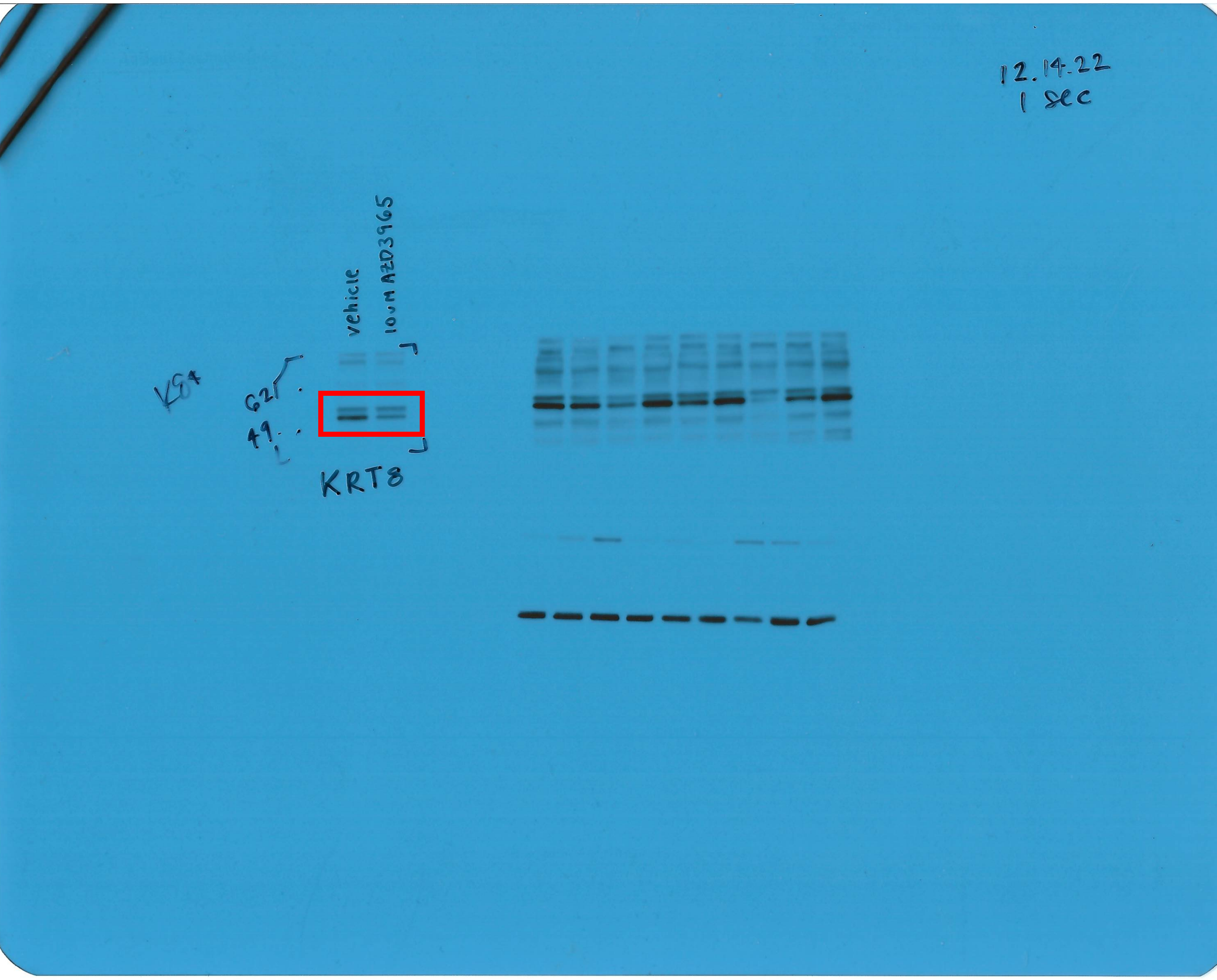

Extended Data Figure 7f

p63

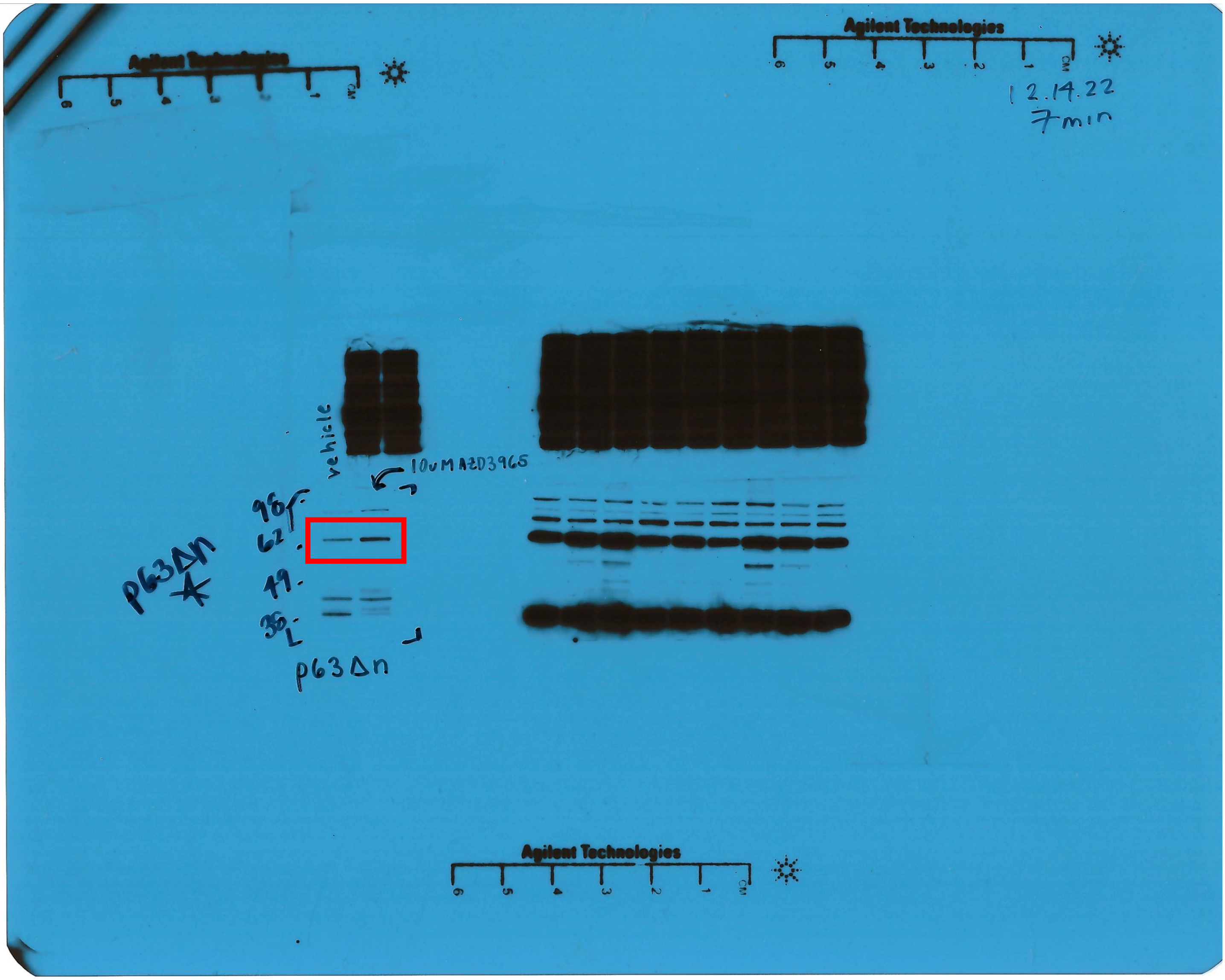

Extended Data Figure 7f

Actin

Vehicle **10uM AZD3965**

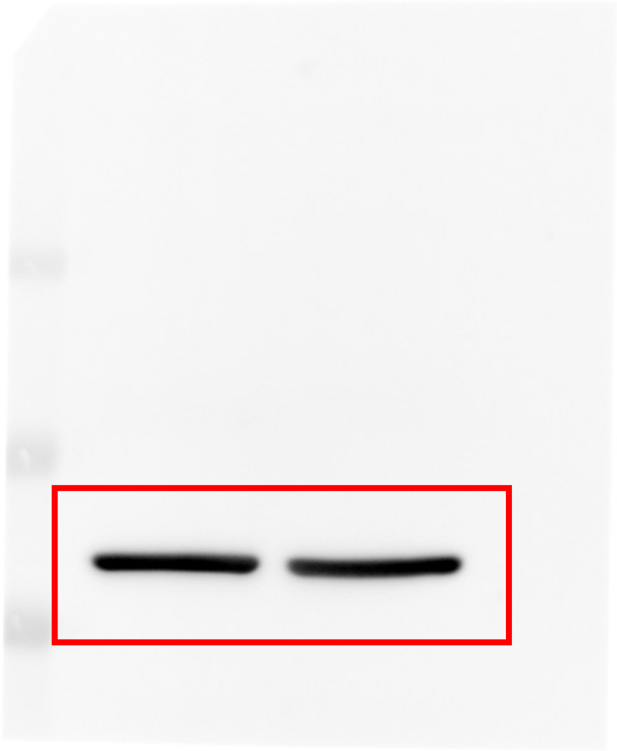

62 kDa

49 kDa

38 kDa

Extended Data Figure 7i

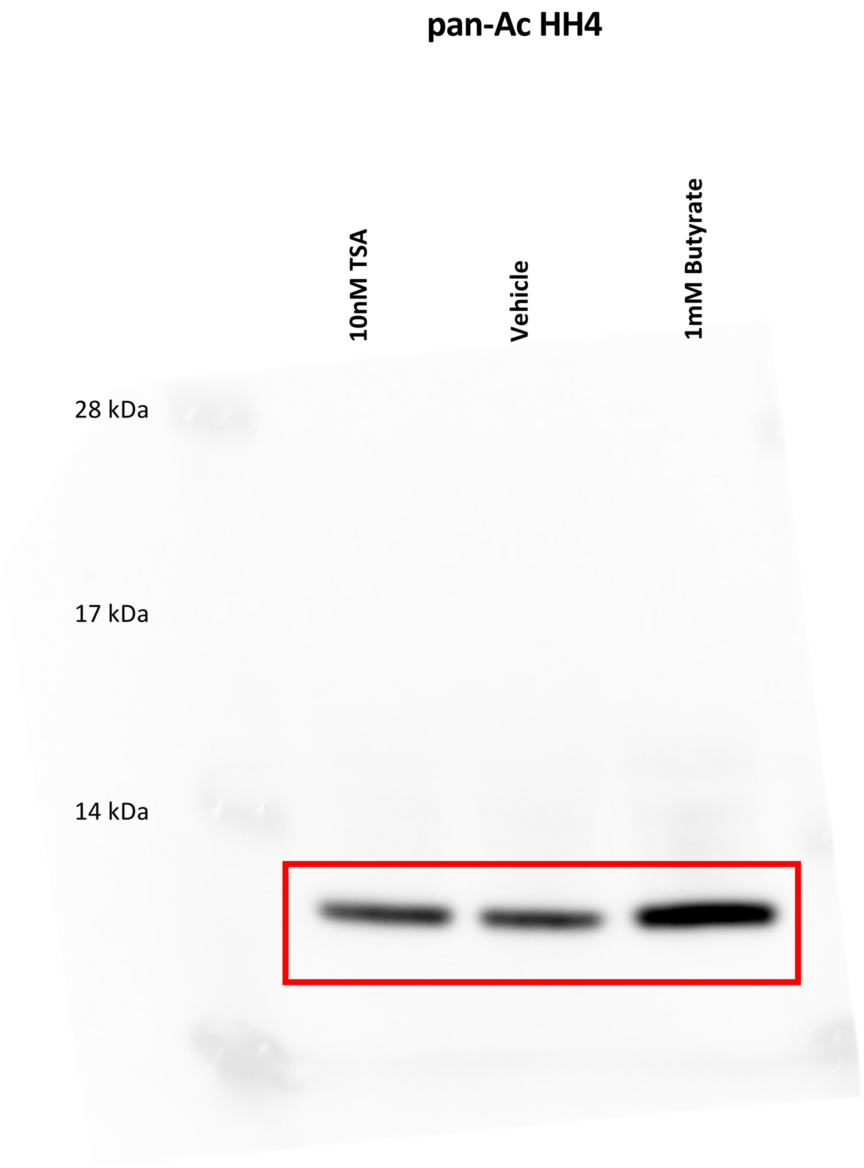

Extended Data Figure 7i

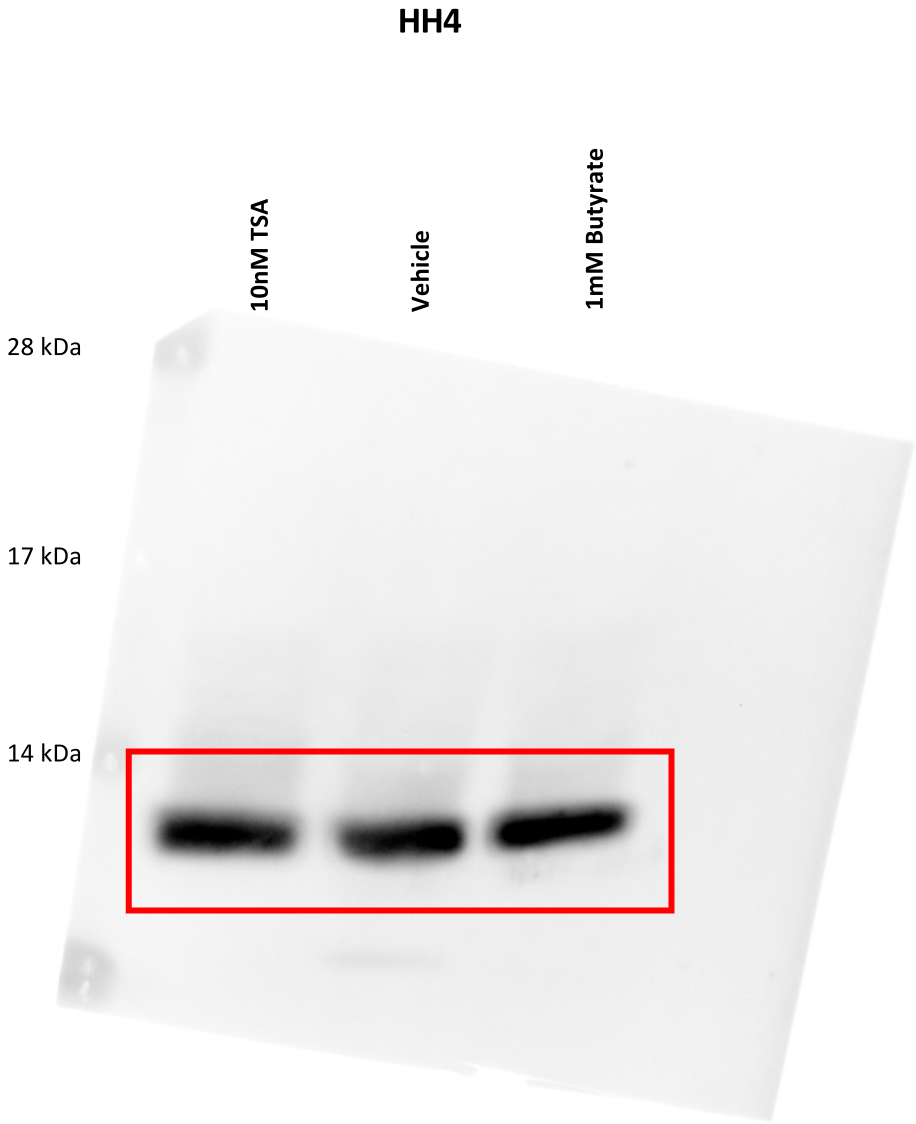

Extended Data Figure 7i

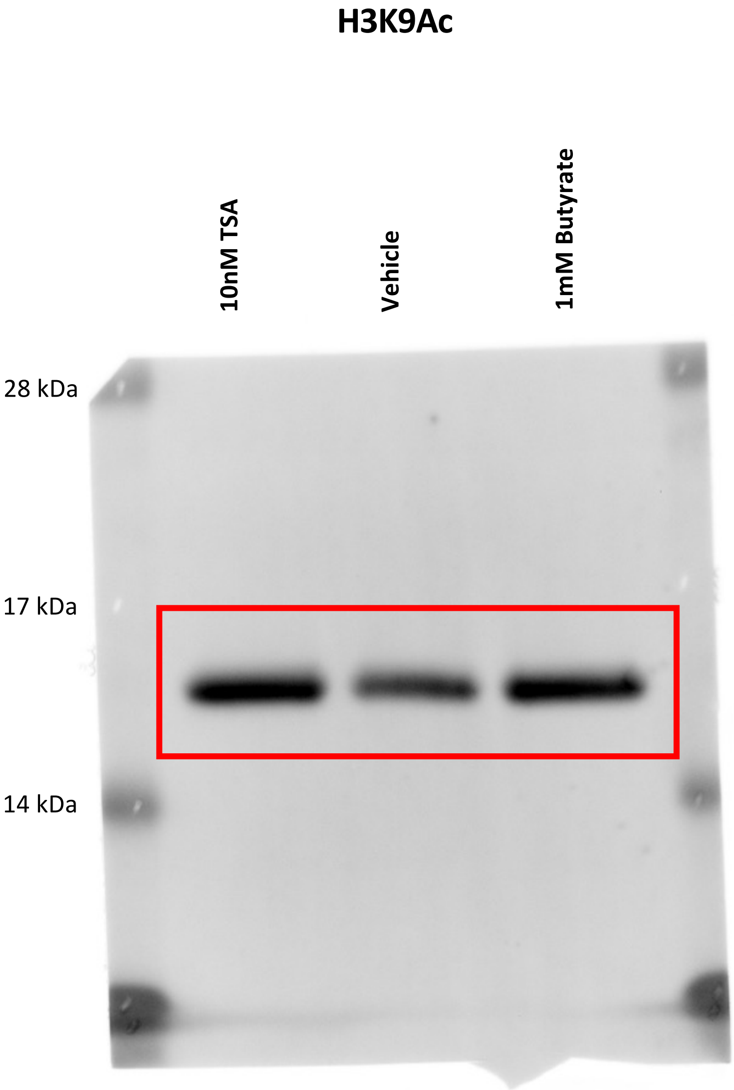

Extended Data Figure 7i

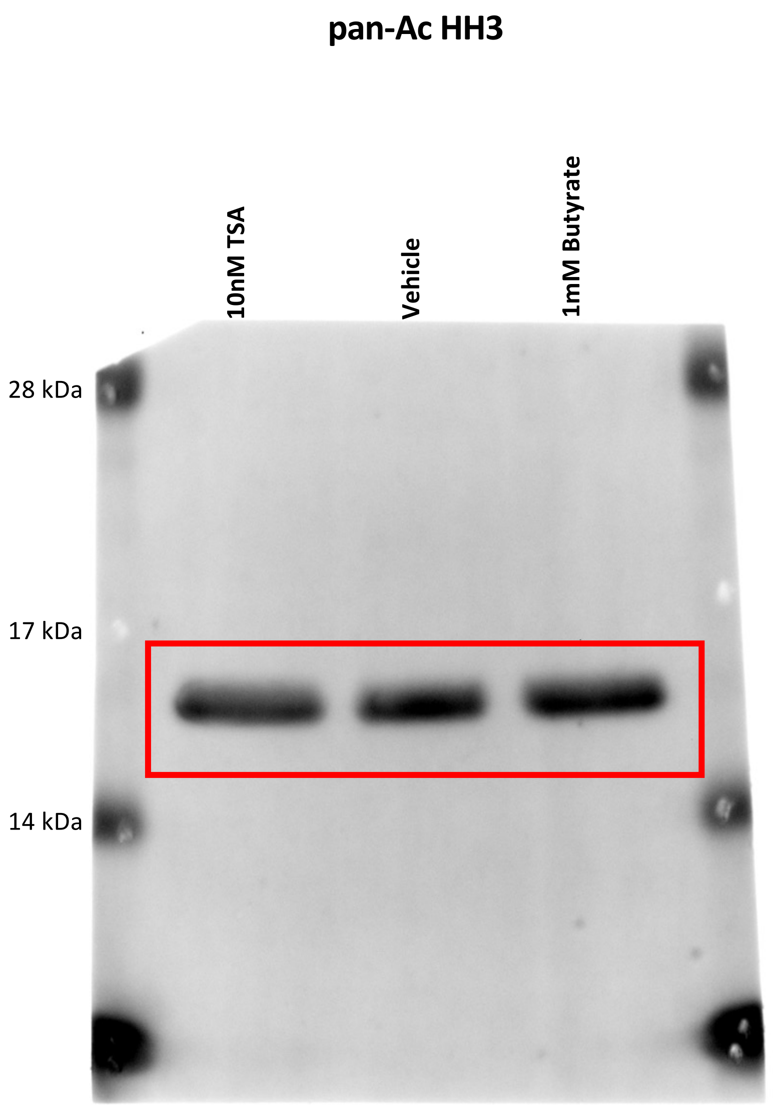

Extended Data Figure 7i

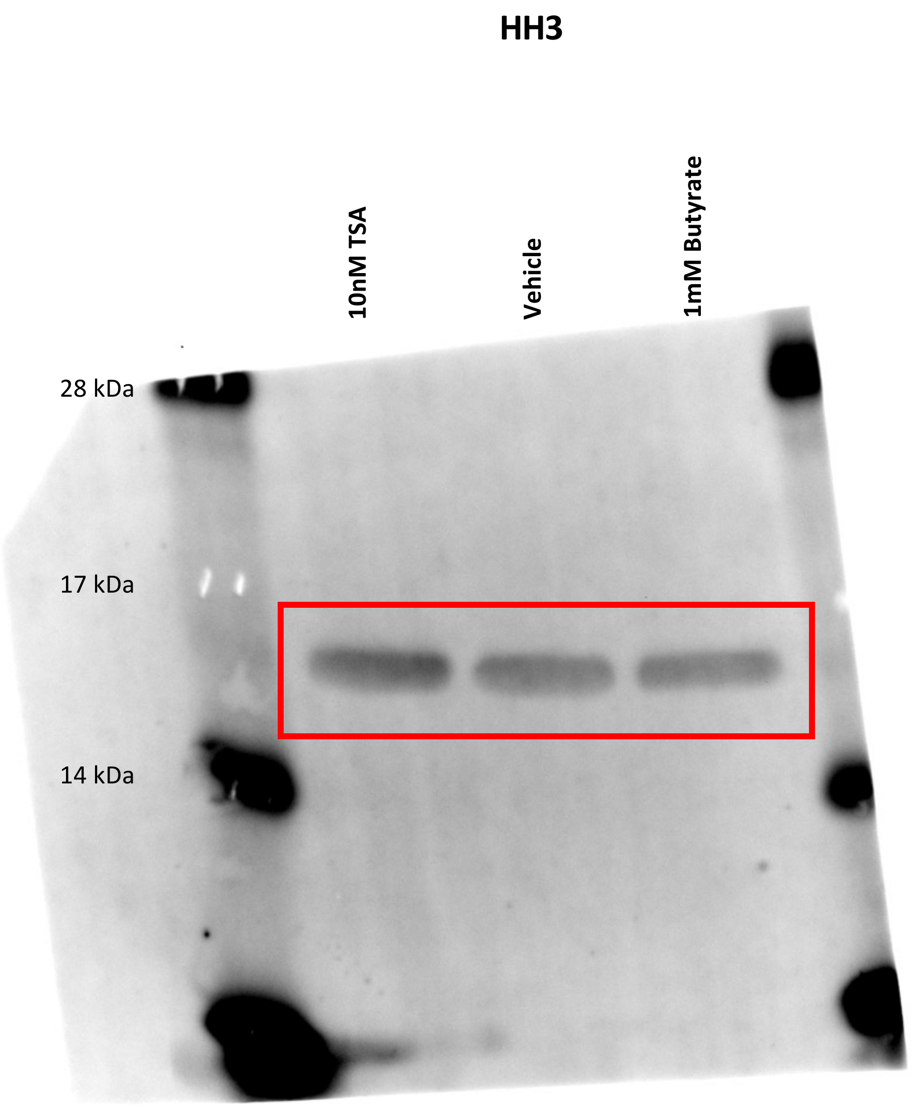

Extended Data Figure 10j

PSA

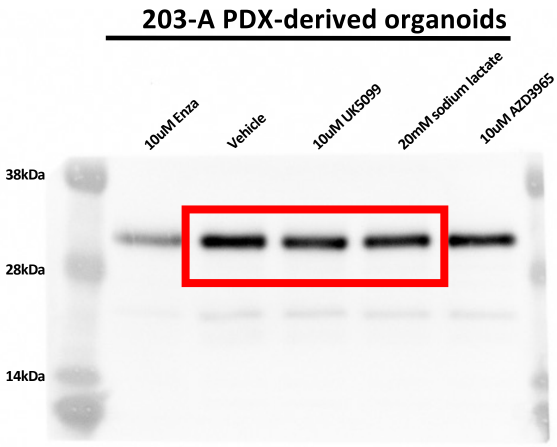

Extended Data Figure 10j

SYP

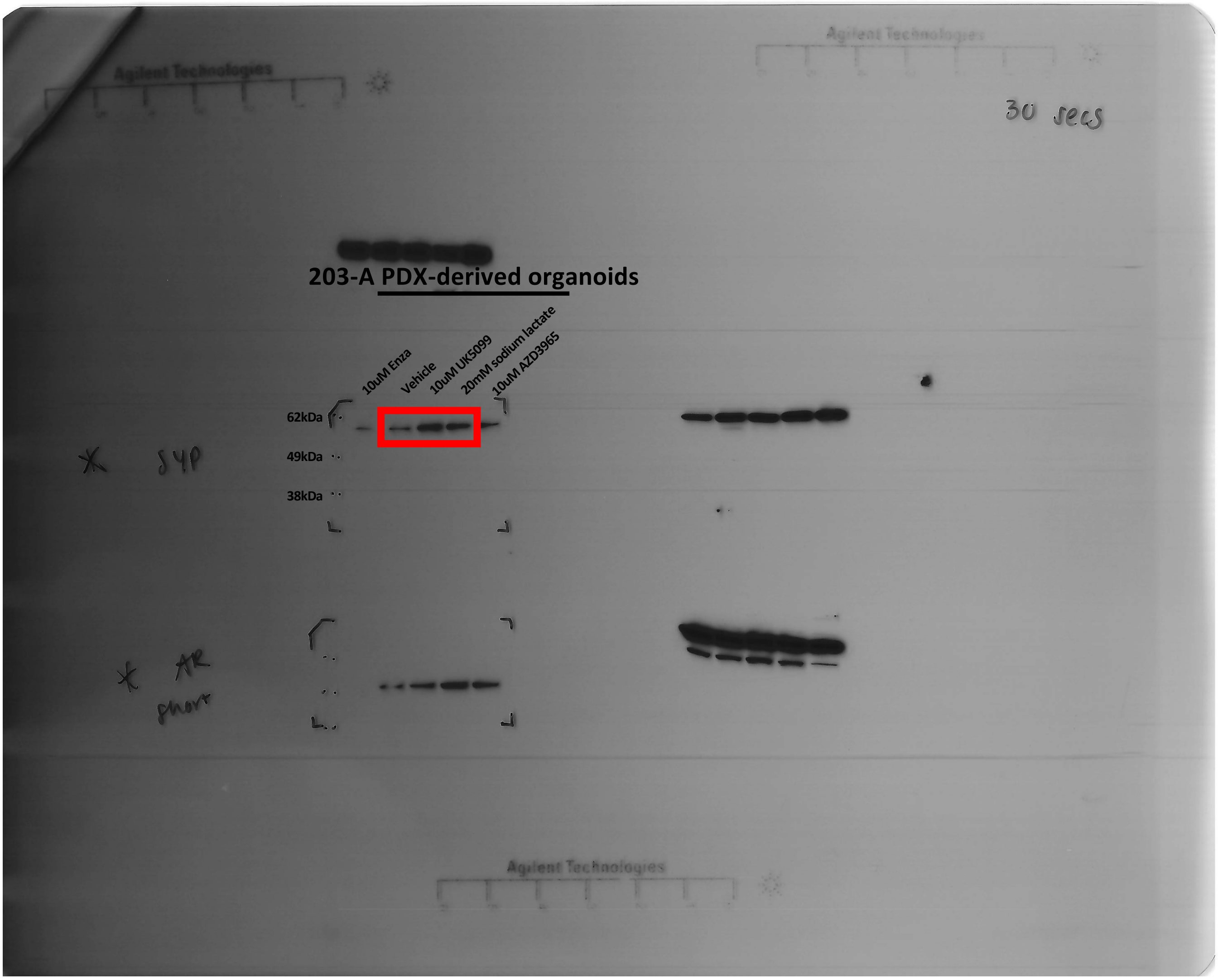

Extended Data Figure 10j

Actin

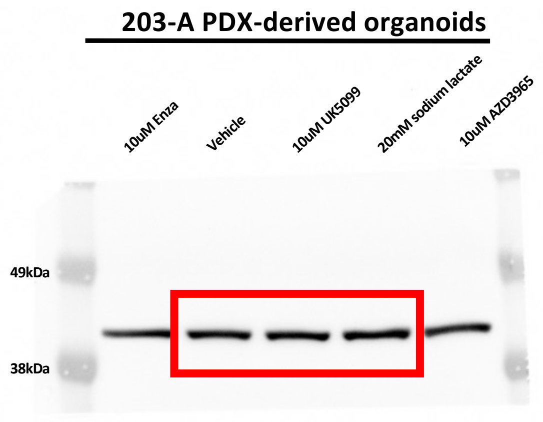

Supplement: Supplementary file 4 — All unprocessed western blots. [file 41556_2023_1274_MOESM4_ESM.pdf]
